# DNA breakpoint assay reveals a majority of gross duplications occur in tandem reducing VUS classifications in breast cancer predisposition genes

**DOI:** 10.1038/s41436-018-0092-7

**Published:** 2018-07-28

**Authors:** Marcy E. Richardson, Hansook Chong, Wenbo Mu, Blair R. Conner, Vickie Hsuan, Sara Willett, Stephanie Lam, Pei Tsai, Tina Pesaran, Adam C. Chamberlin, Min-Sun Park, Phillip Gray, Rachid Karam, Aaron Elliott

**Affiliations:** Department of Clinical Genomics, Ambry Genetics, 15 Argonaut Drive, Aliso Viejo, California 92656 USA

**Keywords:** Tandem duplication, Breakpoint, VUS, HBOC, Alu

## Abstract

**Purpose:**

Gross duplications are ambiguous in terms of clinical interpretation due to the limitations of the detection methods that cannot infer their context, namely, whether they occur in tandem or are duplicated and inserted elsewhere in the genome. We investigated the proportion of gross duplications occurring in tandem in breast cancer predisposition genes with the intent of informing their classifications.

**Methods:**

The DNA breakpoint assay (DBA) is a custom, paired-end, next-generation sequencing (NGS) method designed to capture and detect deep-intronic DNA breakpoints in gross duplications in *BRCA1*, *BRCA2*, *ATM*, *CDH1*, *PALB2*, and *CHEK2*.

**Results:**

DBA allowed us to ascertain breakpoints for 44 unique gross duplications from 147 probands. We determined that the duplications occurred in tandem in 114 (78%) carriers from this cohort, while the remainder have unknown tandem status. Among the tandem gross duplications that were eligible for reclassification, 95% of them were upgraded to pathogenic.

**Conclusion:**

DBA is a novel, high-throughput, NGS-based method that informs the tandem status, and thereby the classification of, gross duplications. This method revealed that most gross duplications in the investigated genes occurred in tandem and resulted in a pathogenic classification, which helps to secure the necessary treatment options for their carriers.

## Introduction

Pathogenic alterations in *BRCA1, BRCA2, ATM*, *CDH1*, *CHEK2*, and *PALB2* contribute to hereditary breast and ovarian cancer (among others cancers) with various risks and rates of penetrance. The identification of such pathogenic alterations informs a patient’s medical management in terms of prevention, screening, and treatment for these cancers, ultimately leading to improved clinical outcome.^1^ A majority of pathogenic alterations arise from nucleotide substitutions or small insertions/deletions that cause nonsense, frameshift, and splicing alterations leading to either nonsense-mediated RNA decay or prematurely truncated proteins;^2^ or missense alterations resulting in structural/functional impairment.^3^

A minority of pathogenic alterations arise from gross rearrangements of the gene including deletions and duplications. While gross deletions are straightforward in terms of clinical interpretation, gross duplications are ambiguous due to the nature of the high-throughput methods that detect them: target capture next-generation sequencing (NGS), array comparative genomic hybridization (aCGH), and multiplex ligation-dependent probe amplification (MLPA). These methods can detect copy number increases; however, they do not impart whether the additional copies are in tandem or translocated to another part of the genome. The current American College of Medical Genetics and Genomics (ACMG) guidelines do not specifically address the interpretation of gross duplications^4^ and in the absence of additional lines of evidence, gross duplications are typically classified as variants of unknown significance (VUS). The aim of this study was to discern the tandemness of gross duplications in *ATM*, *BRCA1*, *BRCA2*, *CDH1*, *CHEK2*, and *PALB2* using a custom, high-throughput, paired-end NGS method. Results revealed a vast majority of the assayed gross duplications found to be in tandem are largely Alu-mediated. VUS rates for gross duplications have dropped from 86 to 5% for reclassification-eligible alterations directly impacting the clinical management of the carriers of these duplications.

## Materials and methods

### DNA samples

At least 6~7 µg of genomic DNA was extracted from whole blood or saliva using the QiaSymphony instrument (Qiagen, Hilden, Germany) according to the manufacturer’s instructions. Isolated DNA was quantified using a NanoDrop UV spectrophotometer (Thermo Fisher Scientific, Waltham, MA, USA) and/or Qubit Fluorometer (Thermo Fisher Scientific) with quality metrics of A260/280 = 1.8–2.0. and A260/230 > 1.6. Breakpoint analysis was conducted on previously characterized, archived, genomic DNA samples and clinical samples sent for testing at Ambry Genetics (Ambry Genetics, Aliso Viejo, CA, USA). All samples selected for this study had a previously identified duplication in *ATM*, *BRCA*1, *BRCA*2, *CDH*1, *CHEK*2, or *PALB2*. Samples were subjected to DNA breakpoint assay (DBA) if DNA samples were still available in accordance with physical and statutory storage limitations for genetic testing institutions and if they met quality standards.

### IRB review

This study was approved and carried out in accordance with the recommendations of the Western Institutional Review Board (WIRB), as part of the protocol titled “Sharing the results of genetic functional assessments performed for subjects previously submitted for clinical genomic testing.” Appropriate informed consent was obtained prior to the collection of study data and the use of these data in analysis and the resulting publication.

### Capture library design

A custom DNA target capture pool was designed to include approximate target breakpoint regions identified based on the VUS duplication data for *ATM, BRCA1, BRCA2, CDH1, CHEK2*, or *PALB2*, from previously run aCGH microarrays (Table 1). A custom aCGH microarray, containing highly tiled probes in exons and less dense probes spaced every 2 kb in introns, was used to detect approximate breakpoints in gross duplication events in the target genes. The sequences of exons, introns or untranslated regions (UTRs) surrounding the identified duplication event were tiled with capture probes to increase the chances of successfully identifying the breakpoints. The DNA capture pool consisted of 3760 biotinylated xGen Lockdown probes synthesized by Integrated DNA Technologies (IDT, Coralville, IA), spanning 426 kb of the human genome (Build37/hg19).

**Table 1:** Tandem duplications identified in breast cancer predisposition genes as identified by breakpoint analysis

Gene	Gross duplication (coding exons)	Effect	Classification before	Classification after	# of tandem/total tested	5’ breakpoint element^d^ (intron)	3’ breakpoint element^d^ (intron)
*ATM (NM_000051)*	EX22_25dup	Frameshift	VUS	Pathogenic	1/1	LINE L1P3	AluSx/+(25)
*BRCA1* (NM_007294)	5’UTRdup	N/A	VUS	VUS	1/1	None (intron 1 of AK311131)	None (5’UTR)
	5’UTR_EX1dup	N/A	VUS	VUS	3/4	None (intron 3 of AK093551)	None (1)
	5’UTR_EX6dup	N/A	VUS	VUS	2/2	None (intron 1 of AK311131)	None (exon 7)
	5’UTR_EX9dup	N/A	VUS	VUS	1/2	AluSc8 (intron 3 of *AK093551)*	None (9)
	5’UTR_EX19dup	N/A	VUS	VUS	1/1	None (intron 3 of AK093551)	AluSc8/− (19)
	5’UTR_EX20dup	N/A	VUS	VUS	2/2	None (intron 2 of MPP2)	AluSc/+(20)
	EX2dup	In-frame	VUS	Pathogenic	1/1	AluSg/+(1)	None (2)
	EX3_11dup	Frameshift	VUS	Pathogenic	2/2	None (2)	None (11)
	EX6dup	Frameshift	VUS	Pathogenic	1/1	AluSc5/+(5)	AluSp/+(6)
	EX11dup	Frameshift	Pathogenic^a^	Pathogenic	21/21	AluSx-AluY-AluSx/+(10)	FLAMC-AluSx1-FLAMC/+(11)
	EX11_12dup	Frameshift	VUS	Pathogenic	2/2	AluSx-AluY-AluSx/+(10)	AluSx1/+(12)
	EX11_14dup	In-Frame	VUS^b^	VUS	1/1	AluSx-AluY-AluSx/+(10)	AluSp/+(14)
	EX12_14dup	Frameshift	VUS	Pathogenic	1/2	FLAMC-AluSx1-FLAMC/+(11)	AluSp/+(14)
	EX16_17dup	Frameshift	VLP	Pathogenic	5/5	AluSg/+(15)	AluSz/+(17)
	EX16_18dup	Frameshift	VUS	Pathogenic	5/5	AluSg/+(15)	AluSq2/+(18)
	EX19_20dup	In-frame	VLP	Pathogenic	4/4	AluSg/+(18)	AluSz/+(20)
	EX21_3’UTRdup	N/A	VUS	VUS	1/1	AluSz/+(20)	MIR1_Amn (3’ downstream)
*BRCA2* (NM_000059)	EX3dup	Frameshift	VUS	Pathogenic	1/1	AluY/− (2)	AluY/- (3)
	EX4_10dup(Partial)	Frameshift	VUS	Pathogenic	1/1	AluY/− (3)	None (exon 10)
	EX11_12dup	Frameshift	VUS	Pathogenic	2/2	LINE L4 (10)	AluSz6/+(12)
	EX11_17dup	Frameshift	VUS	Pathogenic	1/1	None (11)	AluSg4/- (17)
	EX12dup	Frameshift	VUS	Pathogenic	1/1	AluSq10/− (11)	AluSc8/- (12)
	EX13_23dup/trip	Frameshift	VUS	Pathogenic	2/2	LINE L2a (12)	LINE L2a (23)
	EX14_17dup	Frameshift	VUS	Pathogenic	2/2	AluSx /+(13)	AluSz/+(17)
	EX19dup	Frameshift	VUS	Pathogenic	1/1	None (exon 18)	None (19)
*CDH1* (NM_004360)	IN2dup (a)	N/A	VUS	VUS	2/2	None (2)	None (2)
	IN2dup (b)			VUS	10/10	AluSp/− (2)	AluSz/- (2)
	EX3_3’UTRdup	N/A	VUS	VUS	1/2	LINE L2a (2)	MIRc (intron 7 of *TANG06*)
*CHEK2* (NM_007194)	5’UTR_EX1dup	N/A	VUS	VUS	1/1	None (intron 1 of ZNRF3)	MER51A [LTR] (1)
	EX2_3dup (a)			VLP	2/2	AluSc8/+(1)	AluSp/+(3)
	EX2_3dup (b)	In-frame	VUS	VLP	1/1	None (1)	None (3)
	EX2_3’UTRdup	N/A	VUS	VUS	34/35	AluSx-AluSp-AluSx/+(1)	AluSx/+ (intron 1 of *TTC28*)
	EX5_6dup	Frameshift	VUS	Pathogenic	1/1	AluY/+(4)	AluY/+(6)
*PALB2* (NM_024675)	EX11dup (a)	Frameshift	VUS	Pathogenic	3/3	AluYa5/+(11)	AluY/+(12)
	EX11dup (b)			Pathogenic	1/1	AluSz/+(11)	AluSx/+(12)
	EX13_3’UTRdup	Splicing^c^	VUS	Pathogenic	1/1	AluY/+(12)−Estimated w/i 300nt	None (intron 2 of NDUFAB1)

*VUS* variant of unknown significance>, *VLP* variant likely pathogenic, *UTR* untranslated region

^a^Previously established as a well-known founder pathogenic variant

^b^Small in-frame gross duplication in an unstructured region of *BRCA1* leading to uncertain structural impact

^c^Tandem finding in concert with a publication at the time of assay led to reclassification of this variant due to the activation of a cryptic donor in the last exon leading to frameshift

^d^Element orientation relative to the gene is indicated when possible (elements located in other genes do not have orientation indicated): (+) same orientation as the gene; (−) opposite orientation as the gene

### Target enrichment and NGS library preparation

1 µg of genomic DNA per sample was processed using the Kapa Hyper Prep Kit following manufacturer’s recommended protocol (Kapa Biosystems, Inc., Wilmington, MA, USA). Briefly, samples were enzymatically sheared, A-tailed, and ligated to standard Illumina dual indexed adapters. Libraries were purified using AMPure XP beads (Beckman Coulter, Brea, CA, USA) and amplified in a Bio-Rad T100 Thermocycler (Bio-Rad, Hercules, CA, USA). Libraries were again purified using AMPure XP beads and validated with the TapeStation D1000 ScreenTape (Agilent Technologies, Santa Clara, CA, USA). Libraries were then hybridized to biotinylated target probes for the DBA following manufacturer’s recommended protocol (IDT). After hybridization, the captured DNA was captured with magnetic streptavidin beads and purified with several wash buffers in order of decreasing stringency. Libraries were again briefly amplified and purified using AMPure XP beads before final validation on with the TapeStation D1000 ScreenTape (Agilent Technologies). Up to 96 samples were normalized and pooled together for sequencing. Sequencing was conducted on the Illumina NextSeq 500 using 150-bp paired-end reads (Illumina Inc, San Diego, CA, USA).

### Next-generation sequencing data analysis and interpretation

Initial data processing and base calling, including extraction of cluster intensities, was done using RTA 1.17.21.3 (Real Time Analysis, HiSeq Control Software version 2.0.10 Illumina Inc., San Diego, CA, USA). Barcode de-multiplexing was done with the bcl2fastq Conversion Software v1.8 or CASAVA v.1.8.2 (Illumina Inc., San Diego, CA, USA). DNA samples passed sequencing quality control (QC) criteria (percentage of Q30 bases >75%, mean base quality >30 and percentage of perfect index >85%) were used for downstream analysis. Sequence reads were aligned to the reference human genome (GRCh37) using Novoalign v.3.02.07 (Novocraft Technologies, Selangor, Malaysia). Downstream data processing which includes removal of duplicated reads, indel realignment and base quality recalibration was done with Picard v1.1.1 and Genome Analysis Toolkit (GATK) v3.2.2 (Broad Institute, Cambridge, MA, USA) following GATK Best Practice recommendation. Pindel v0.2.5 (ref.^5^) and Delly v0.6.1 (ref.^6^) were used to call duplication breakpoints. Breakpoints were further examined with Integrative Genomics Viewers (Broad Institute, Cambridge, MA, USA) and confirmed by experienced analysts. Variants are then classified following the American College of Medical Genetics and Genomics and International Agency for Research on Cancer variant classification recommendations.^4,7^

## Results

### Gross duplications tested in the DBA

**Fig. 1:**
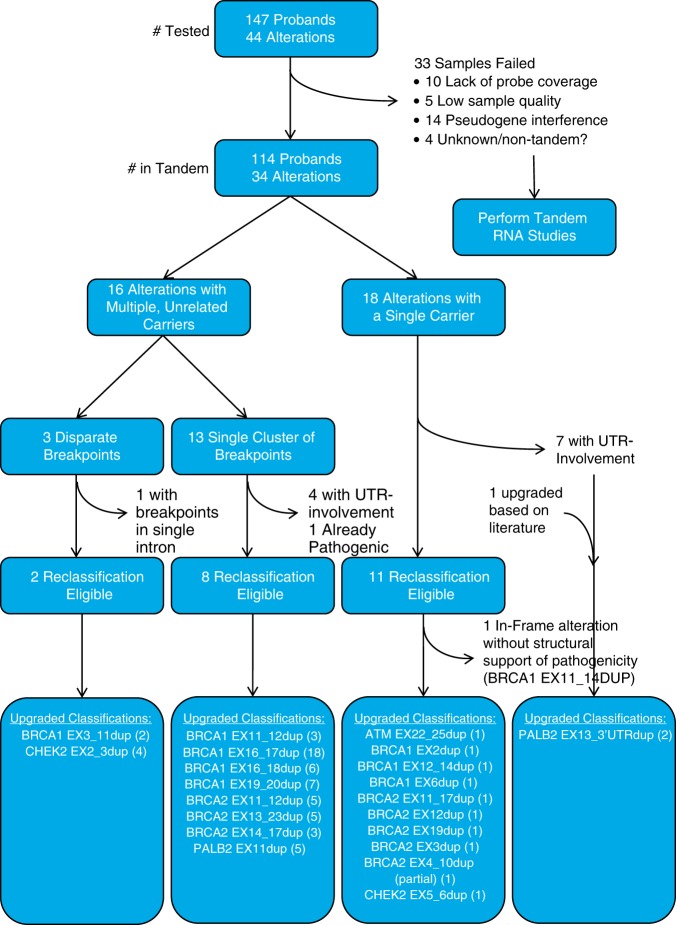
Flow chart of DNA breakpoint assay (DBA) from sample entry to variant classification. The number of samples, families, and unique alterations entering the DBA (level 1) filtered by successful DBA and tandem finding (level 2); multiple, unrelated carriers versus a single carrier (level 3); breakpoint clustering versus disparate breakpoints (level 4); variants eligible for reclassification (level 5); and finally, variants that were assigned a pathogenic classification with number of individuals receiving a positive test result represented in parentheses (level 6). UTR untranslated region

**Fig. 2:**
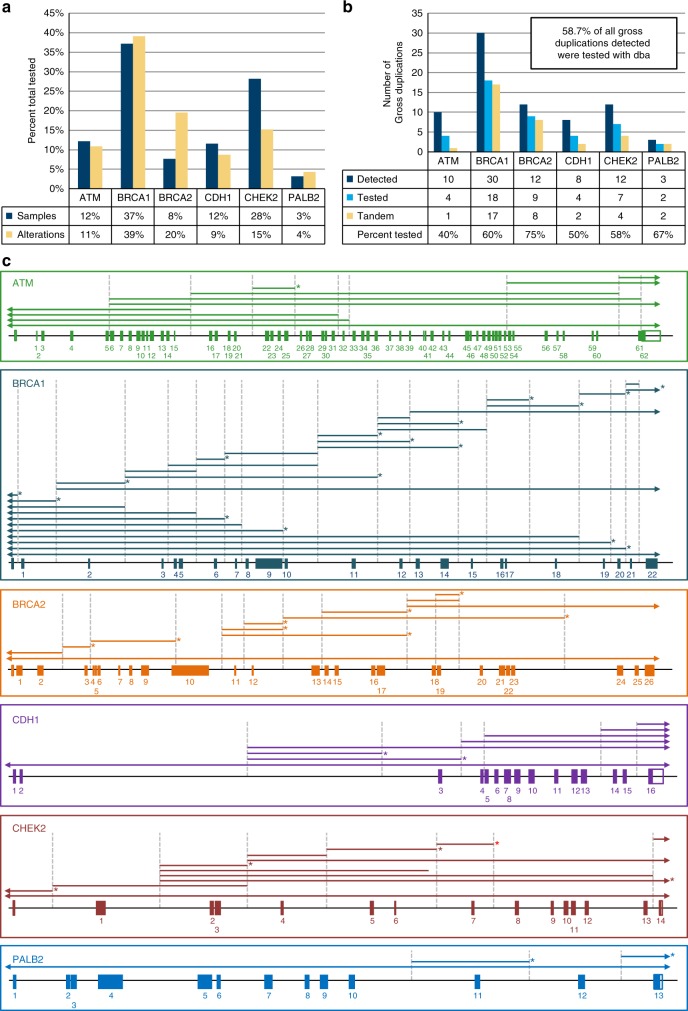
Tested cohort of samples and alterations per gene. **a** Percent of samples (blue) and alterations (red) of the gross duplications assayed in this cohort per gene. **b** Number of duplications detected (blue), tested (red), and identified as tandem (green) in this clinical cohort per gene. The percent of gross duplications relative to the number of gross duplications ever detected in this clinical cohort are indicated as Percent Tested per gene at the bottom of the chart. The percent of all gross duplications tested relative to gross duplications detected in the six genes combined is 58.7% (top right box in the graph). **c** All gross duplications identified in the clinical cohort are depicted roughly to scale for the six genes assayed in the DNA breakpoint assay (DBA). Duplications that had at least one proband with a tandem finding are indicated by * after the line representing that duplication. The red * indicates *CHEK2* Ex7dup, which was identified as a tandem duplication after RNA analysis, not DBA. Note that this depiction does not imply to scale the location of identified breakpoints; rather breakpoints are randomly placed near the middle of the involved intron. Dashed vertical lines are placed as a visual aide

One hundred forty-seven probands with previously identified gross duplications underwent DBA: 19 in *ATM* (NM_000051), 58 in *BRCA1* (NM_007294), 12 in *BRCA2* (NM_000059), 18 in *CDH1* (NM_004360), 44 in *CHEK2* (NM_007194), and 5 in *PALB2* (NM_024675) (Fig. S1; Table S1; Fig. 1; Fig. 2). These 147 probands harbored 44 unique gross duplications: 4 in *ATM*, 18 in *BRCA1*, 9 in *BRCA2*, 4 in *CDH1*, 7 in *CHEK2*, and 2 in *PALB2* (Fig. 2b; Table 1; Table S1). All gross duplications are reported according to Human Genome Variation Society (HGVS) nomenclature and are reported using coding exon nomenclature. These samples were tested based on their availability due to storage and statutory limitations of clinical diagnostic laboratories but still represent a substantial subset (58.7%) of all gross duplications detected in this clinical cohort (Fig. 2b). This cohort of unique gross duplications represents 40% of all *ATM*, 60% of all *BRCA1*, 75% of all *BRCA2*, 50% of all *CDH1*, 58% of all *CHEK2*, and 67% of all *PALB2* reportable unique gross duplications identified in this clinical cohort (Fig. 2b, c). The carriers of these gross duplications were most frequently affected with unilateral breast cancer and the only genotype–phenotype correlation was the presence of a larger proportion of triple-negative breast cancer in the cohort of *BRCA1* gross duplication carriers (Table 2).

**Table 2:** Personal cancer history (127 cancer diagnoses among 147 carriers and 9 carrier family members)

	*Unilateral breast*	*Multiple breast primaries*	*% TNBC* ^*a*^	*Ovarian*	*Pancreatic*	*Other* ^*b*^	*Total cancer diagnoses*	*Cases with no cancer diagnoses*
*ATM*	11	2	0%	2	2	4	23	2
*BRCA1*	28	2	33/59%	3	1	4	40	23
*BRCA2*	9	1^c^	0%	0	0	1	13	2
*CDH1*	8	4	9/13%	1	0	2	19	4
*CHEK2*	17	5	13/24%	4	0	11	42	14
*PALB2*	2	1	0%	0	0	2	6	1

In six cases, a co-occurring pathogenic variant was also identified in an individual with a gross duplication; however each gross duplication was UTR-involved and remains a variant of uncertain significance (VUS) after the conclusion of this study. Two of these co-occurrences account for two of the TNBC diagnoses: one each for the *BRCA1* and *CHEK2* categories

^a^Percent of individuals with a triple-negative breast cancer (TNBC) diagnosis relative to all breast cancer diagnoses (first percentage) and relative to all breast cancer diagnoses with reported hormone status (second percentage)

^b^Other cancers include basal cell carcinoma, lung, medulloblastoma, thyroid, melanoma, colorectal, endometrial, kidney, bladder, appendiceal, and lymphoma

^c^This individual had three primary breast cancers

### Tandem findings

Of 147 probands, 114 (78%) had a tandem finding after DBA in one of 34 unique gross duplications (Table 1; Table S1, Fig. S2). Six families had multiple carrier family members tested and each had identical breakpoints identified (Table S1, foonote a). Tandem breakpoints were not identified in the remaining 33 samples due to a suspected technical failure (see Table S1; Table S2) or an unknown reason including the possibility that the duplication is not in tandem. It is important to note that a failure to produce an NGS read cannot distinguish between a suspected technical failure and a nontandem insertion because both events will have the same result.

Of note, any alteration that is reclassification-eligible (see below) that fails DBA is eligible for tandem reverse transcription polymerase chain reaction (RT-PCR) investigation. *CHEK2* EX7dup was one such gross duplication that failed the DBA due to a suspected lack of probe coverage (Table S2). Follow-up tandem RT-PCR analysis showed this alteration was in tandem and that lack of probe coverage, not a nontandem insertion, was the likely cause of the DBA failure. Tandem RT-PCR results show the tandem insertion encodes an in-frame stop codon after the normally encoded exon 7 and is expected to have a loss-of-function (LoF) effect (Fig. S3).

Only 4/147 (2.7%) tested samples failed DBA for an unknown reason (Table S2). However, two of these failed samples had an additional proband with the “same” alteration identified in tandem increasing the likelihood that these unknown sample failures are due to an unknown sample issue, rather than a nontandem event. Overall, excluding samples whose failure was due to suspected technical reasons, 114/118 (97%) of total tested samples were identified as tandem duplications.

### Breakpoint characterization

#### Repeat elements

Analysis of the surrounding genomic structure was conducted on alterations where breakpoints were identified. Three alterations had two sets of disparate breakpoints: *CDH1* IN2dup, *CHEK2* EX2_3dup, and *PALB2* EX11dup and these will be considered as six *different* alterations. Of 37 tandem alterations 27 (73%) have at least one breakpoint within an Alu element and 17/37 (46%) had *both* breakpoints within Alu elements (Table 1, Fig. S4) highlighting the importance of Alu elements in the gross duplication mechanism. The Alu elements were always oriented in the same direction as each other, and in all but three cases, they were also oriented in the same direction as the transcription of the gene that harbored them. A majority of these Alu-involved tandem duplications occurred in *BRCA1*, which is unusually enriched in Alu elements.^8^ A number of the Alu elements involved in *BRCA1* gross duplications identified in this study have not, to our knowledge, been previously reported, while others are recurrent (Table 1; Fig. S5) (refs.^9^–^14^).

Of 37 tandem alterations 10 (27%) had no identifiable repeat elements at either breakpoint and 3/37 (8%) had either one or both breakpoints within a non-Alu repeat element (e.g., LINE, MIR, LTR). Interestingly, among these three, a single case—*BRCA2* EX13_23dup/trip—showed multiple probands with both breakpoints occurring in LINE L2a elements. Of note, depending on the detection method, this alteration cannot be confidently distinguished as a duplication or a triplication and the breakpoint assay will also not make this distinction; however, the interpretation of this variant is the same whether it occurs as a duplication or a triplication.

#### Exonic breakpoints

In three cases, one breakpoint was found to be exonic. *BRCA1* 5’UTR_EX6dup had a 3’ breakpoint in exon 7 near *BRCA1* c.558. Of note, aCGH was the original detection method for both probands and in this clinical diagnostic laboratory, the probe coverage for this assay does not have sufficient resolution to detect the inclusion of this part of exon 7 in this gross duplication. *BRCA2* EX19dup had a 5′ breakpoint in exon 18 near *BRCA2* c.8433. This individual originally had the gross duplication detected by MLPA, which also lacks sufficient resolution to detect the inclusion of part of exon 18 in this gross duplication event. Lastly, *BRCA2* EX4_10 (partial) was confirmed to have the 3′ breakpoint within exon 10. This alteration was originally detected with aCGH and because exon 10 is so large, there are multiple probes within this exon allowing it to detect a partial exon 10 duplication event. After breakpoint analysis, the actual breakpoint was found to occur around *BRCA2* c.4780 between BRC repeats 4 and 5 (Table S1).

#### Nonrecurrent breakpoints

Of 37 tandem duplications, 3 had probands with *different* sets of breakpoints: *CDH1* IN2dup, *CHEK2* EX2_3dup, and *PALB2* EX11dup, indicating that these alterations are the product of at least two independent duplication events. For *CDH1* IN2dup, 12 unrelated individuals were tested and found to have two different sets of recurrent breakpoints, perhaps owing to the unusually large size of this intron, which is approximately 63 Kb. Of 12 *CDH1* IN2dup carriers, 2 had 5’ breakpoints around *CDH1* c.163 + 4806 and 3′ breakpoints around *CDH1* c.164−16867, neither of which overlapped with any identifiable repeat elements (Table 1; Table S1, demarcated by [a]). Of 12 unrelated carriers, 10 had 5′ breakpoints near *CDH1* c.163+29696 (AluSp) and 3′ breakpoints near *CDH1* c.164−9281 (AluSz) (Table 1; Table S1, demarcated by [b]). For *CHEK2* EX2_3dup, three unrelated individuals were tested. Each proband had 5′ breakpoints within an AluSc8 in intron 1, however, one proband had a disparate 3′ breakpoint (intron 3–no repeat element) that was around 3963 nucleotides downstream from the 3′ breakpoints of the other two probands (intron 3–AluSp) (Table 1, Table S1). For *PALB2* EX11dup, two sets of breakpoints were identified. One family had breakpoints in an AluYa5 and AluY element (5′: intron 10 and 3′: intron 11, respectively) while another proband had both breakpoints upstream of each of those breakpoints in an AluSz and AluSx element, respectively (Table 1, Table S1).

The remaining 31 tandem gross duplications with multiple tested probands showed identical breakpoints or breakpoint clustering. In most cases where breakpoints were Alu-associated, the clustered breakpoints fell within the same Alu element. In general such clustered breakpoints were within 100 nucleotides or less of each other.

#### Classification of tandem gross duplications

**Fig. 3:**
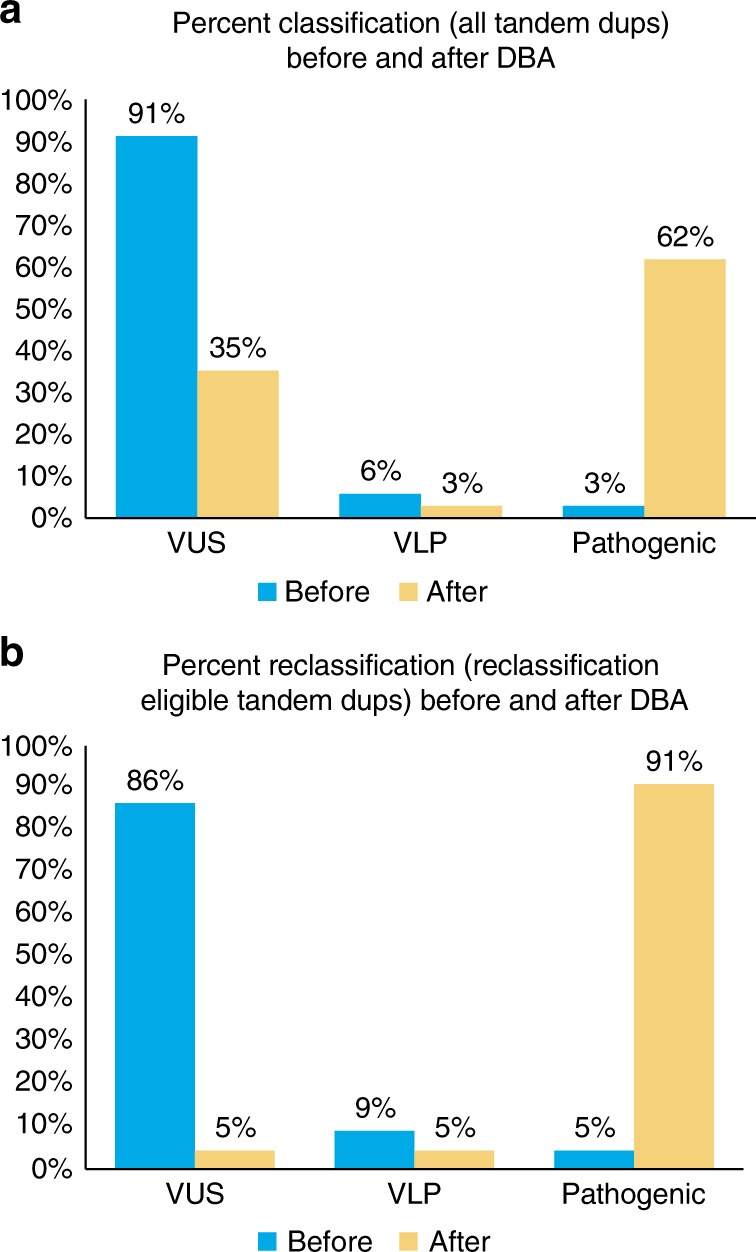
Classification rates for gross duplications before and after DNA breakpoint assay (DBA). **a** Percent classification ascribed to each tandem gross duplication before and after DBA tandem finding. **b** Percent classification ascribed to each reclassification-eligible gross duplication before and after DBA tandem finding. Eligible gross duplications must have been identified in tandem, must not have involved a 5′ or 3′ UTR/up- or downstream region, and must have not been contained within a single intron. This does not include PALB2 Ex13_3′UTRdup, which was reclassified as pathogenic in light of 3′-UTR involvement (see text). VUS variant of uncertain significance; VLP very likely pathogenic

Before DBA, 31/34 gross duplications were classified as VUS (Table 1, Fig. 3). All variants were reviewed for reclassification based on tandem findings. Alterations were eligible for a pathogenic/variant likely pathogenic (VLP) classification if they met the following criteria: (1) they were identified in tandem; (2) they did not involve an untranslated region (UTR); (3) they were not completely contained within a single intron; and (4) they were not already classified as pathogenic based on other evidence (Fig. S6). Based on these criteria, 21 gross duplications were reclassification-eligible. Of those that did not satisfy these criteria, one was already pathogenic (*BRCA1* EX11dup); one was completely intronic (*CDH1* IN2dup); and 11 were UTR-involved (Table 1).

Of 21 reclassification-eligible gross duplications, 20 (95%) were ultimately reclassified as pathogenic/VLP due to a predicted frameshift and alternate stop codon^3^ or based on structural evaluation due to predicted interference with clinically important functional domains as detailed below and in Fig. S7.

For BRCA1 EX2dup, the duplicated portion is part of the N-terminal RING finger domain, which contains two separated Zn^+2^ binding sites.^15^ The 3D X-ray structure of the RING domain complexed with BARD1 is solved^16^ and this region is known to interact with other proteins^17^ indicating that alterations here can potentially disrupt protein–protein interactions (Fig. S7). The structural model of the tandem duplication of exon 2 was constructed and compared with wildtype (Fig. S7). The duplicated fragment is in close contact with the second Zn^2+^ loop, in which multiple pathogenic RING domain variants are found. This suggests that the tandem duplication of exon 2 would be similarly structurally deleterious by disrupting the structure and function of the second Zn^2+^ loop.

For BRCA1 EX19_20dup, the C-terminal portion of BRCA1 contains two BRCT repeats that together form a surface, which is required for interacting with phosphorylated proteins such as a BACH1 (ref.^18^). Disruption of the BACH1 binding site (Fig. S7b; cyan cartoon) or BRCT repeats is a known mechanism of pathogenicity.^19,20^ BRCA1 EX19_20dup repeats a significant portion of the BRCT2 domain (BRCA1 C-terminus repeat 2). The boundaries of the duplication indicate it would be folded along with the normal BRCT domains. Sequence alignment positioning and structural modeling resulted in the duplication occurring at the first alpha helix of BRCT2 domain (Fig. S7b; red surface). This addition interferes with normal BRCT1/2 folding and is anticipated to cause a malformed phospho-binding cleft. Alternative structured positions result in the insertion occurring in the N-terminal region (Fig. S7b; green surface) and this position is anticipated to distort the overall structure of the folded protein. Although there are several plausible orientations, structural modeling of the duplicated BRCT2 domain is anticipated to be pathogenic through a misfolded BRCT repeat region with a malformed phospho-binding cleft.

For CHEK2 EX2_3dup, the amino-terminal forkhead-associated (FHA) domain is needed for the initial steps of protein activation, which include dimerization of inactive monomers, leading to autophosphorylation, and activation.^21^ CHEK2 EX2_3dup results in a duplication of a significant portion of the FHA domain. Structural modeling of the duplicated region positions it near the center of the FHA domain (Fig. S7c). Insertions inside or at either end of the FHA domain are anticipated to either directly disrupt binding between CHEK2 monomers or significantly distort the structure of the FHA domain. Insertion of the protein at the N-terminal end (Fig. S7c; red surface) distorts the structure of the folded protein and likely impacts the oligomerization between FHA domains, whereas positioning the insertion at the C-terminal end (green surface) directly interferes with binding between the two FHA domains of the dimer. Disruptions of the oligomerization of the FHA domain or alterations at key phosphorylation sites (p.T68) in CHEK2 are known mechanisms CHEK2 impairment.^22,23^ CHEK2 EX2_3dup is, thus, expected to disrupt the protein’s normal mode of oligomerization and activation, which is ultimately predicted to lead to an inactive protein.

*BRCA1* EX11_14dup was the only reclassification-eligible alteration that was not classified as pathogenic/VLP because it is a tandem, in-frame duplication in a region where no clinically important domains are known and the structural impact of this alteration could not be predicted (Fig. 1; Table 1; Fig. S6).

*PALB2* EX13_3′UTRdup was also reclassified from VUS to pathogenic, although it would normally be considered ineligible for reclassification due to UTR involvement. DBA identified this alteration as a tandem duplication prompting a variant review that identified new literature. Yang et al. also identified this alteration as tandem and showed that it produced an abnormal transcript.^24^ Although not identified by Yang et al., a strong, inactive splice donor is predicted in the last coding exon of *PALB2* (refs.^25^–^27^). This cryptic donor is activated in tandem duplication setting, and is predicted to produce the exact RNA transcript observed by Yang et al. Thus, in conjunction with multiple tandem findings, observed aberrant RNA, and a clear mechanism, the *PALB2* EX13_3′UTRdup alteration was reclassified as pathogenic.

In total, DBA reduced the VUS classification rate of gross duplications from 91 to 35% (Fig. 3a). Of 22 reclassification-eligible gross duplications, 21 (95%) were upgraded to a clinically actionable classification reducing VUS rates from 86% to just 5% for these alterations (Fig. 3b). The DBA directly impacted 70 individuals who can now be confident in positive genetic test results and will now be eligible to receive informed clinical care.

## Discussion

This study examines gross duplications in six LoF cancer predisposition genes to determine tandemness based on an easily implemented NGS-based follow-up method to clarify the clinical interpretation of a gross duplication finding. A vast majority of the gross duplications that were tested that did not have known technical failures were tandem duplications (97%). In most cases, breakpoints were identified in the same genomic location, providing evidence of either a common ancestral origin of a single duplication event or of a DNA region that is sensitive to recurrent breakpoint acquisition.

For clinical purposes, exact breakpoints do not need to be determined to inform the classification of gross duplications based on tandem finding. However, knowing that breakpoints are recurrent among probands provides evidence of a mechanism that allows clinical laboratories to have more confidence that all cases are highly likely to be similar and to apply this knowledge to the classification of future cases of unknown tandem status. This confidence is further bolstered when breakpoints both occur within Alu repeats of the same family. Alu elements, which comprise 11% of the human genome,^28^ provide ample opportunity for Alu-mediated gross rearrangements when initial breakpoints occur at, or near such elements. One model that explains the involvement of Alu sequences in gross alterations is called intrastrand slipped mispairing (ISMP), a nonallelic homologous recombination event that was first postulated in 1985 by Roth et al.^29^ An analogous model called break-induced repair (BIR) is a *mitotic* model that involves RecA/Rad51-mediated repair during DNA replication.^30^ These models start with a single breakpoint that is repaired when the nicked DNA anneals to an extended region with high sequence identity distal from the initial breakpoint followed by DNA synthesis either through repair pathways (ISMP) or DNA replication (BIR). A stretch of 8–30 nt of 100% sequence identity overlapping the patient’s breakpoint junction is evidence for this model.^29,31^ Although this DBA did not make use of direct-sequencing across the breakpoints, in all cases where both breakpoints occurred within an Alu element, sequence alignment of these Alu elements revealed a stretch of 100% sequence identity of between 16 and 48 nucleotides, providing evidence that ISMP and BIR are possible mechanisms of recombination for these alterations (Fig. S8). Although *BRCA1* 5′UTR_EX1dup had neither breakpoint in an Alu element, alignment of the breakpoint regions shows extensive sequence identity (Fig. S8). In every case where both breakpoints occurred in Alu elements, the Alu elements were oriented in the same direction as each other and most cases, they were also oriented in the same direction as gene transcription (Table 1, Figs. S4, S5), a finding that is consistent with previous observations in *BRCA1* (refs.^9^, ^32^). The importance of Alu orientation can now be expanded from *BRCA1* to gross duplications in other genes as well.

Alu-mediated recombination is a recurrent event in *BRCA1*, in particular. In our clinical cohort, *BRCA1* harbors a disproportionate amount of unique gross duplications compared with the other genes in this study (Fig. 2c). There is also an unusually high frequency of Alu elements in this gene relative to the rest of the genome (~41.5%) perhaps making it more prone to gross rearrangements.^33^ The current study adds to the list of Alu elements that are involved in gross rearrangements in *BRCA1* (Fig. S5) (ref.^9^). In some cases, different duplications have exactly the same breakpoints (e.g., *BRCA1* EX16_17dup and *BRCA1* EX16_18dup: intron 15-AluSg). Conversely, some alterations have different breakpoints but within the same Alu element (e.g., *BRCA1* EX11dup, *BRCA1* EX11_12dup, and *BRCA1* EX11_14dup: intron 10-AluSx-AluY-AluSx, but each breakpoint seems to fall within a different part of this element). Further still, some alterations have breakpoints that lie in different Alu elements within the same intron. For example, a complex rearrangement leading to *BRCA1* EX15_18del, *BRCA1* EX16_17del, *BRCA1* EX7_17del, and *BRCA1* EX16_17dup each have breakpoints in one of several different Alu elements in intron 17: two different AluSz elements, an AluSx-AluSp-AluSx, and an AluSz, respectively.^34,35^

In one alteration, *BRCA2* EX13_23dup/trip, both breakpoints were found to cluster in LINE-L2 elements at both ends. LINE-mediated rearrangements are an underrepresented mechanism of copy-number variation.^36^ LINE elements are evolutionarily much older than Alu elements and, thus, show a greater degree of sequence divergence due to the acquisition of numerous insertions and deletions.^37^ As such, with the exception of LINE L1-HS elements, which are exceedingly rare, the majority of LINE elements present in the human genome are remnants of ancient retrotransposition events and are no longer able to “jump” in or out of their native locations. However, these remnants can still provide a substrate for ISMP or BIR if they have homology at the involved breakpoint regions. Surprisingly, the two LINE-L2 elements involved in *BRCA2* EX13_23dup/trip did not have any identifiable sequence identity with each other (Fig. S8) indicating either a different mechanism for duplication with recurrent breakpoints or a different mechanism of a single duplication event that occurred in a common ancestor.

Some less well-established models for such recombination events leading to gross duplications include nonhomologous or microhomology-mediated (also known as “alternative”) end joining (NEHJ and MMEJ);^38,39^ microhomology-mediated break-induced replication (MMBIR);^30^ and fork stalling and template switching (FoSTeS).^40^ MMBIR and FoSTeS, like the homology-mediated model BIR, are considered *mitotic* in that they rely on DNA replication to take place. The basis for these models is that non-B DNA structures cause replication fork stalling, which, in turn, leads to DNA lesions that are repaired by these mechanisms. Sequences that are predicted to form non-B DNA structures were not identified near the breakpoints for *BRCA2* EX13_23dup/trip (data not shown).

This DBA provides a high-throughput method for discerning the tandem nature of gross duplications identified in a clinical diagnostic laboratory. In total, 95% of reclassification-eligible tandem gross duplications were reclassified from VUS to clinically actionable impacting 70 individuals (Fig. 3). These impacted individuals are now eligible to receive earlier cancer screening, risk-reducing medicine, and/or prophylactic surgeries, and the ability to empower their relatives to seek genetic counseling and testing to further reduce the impact of cancer in their families.

## Electronic supplementary material

Supplementary Figure S1

Supplementary Table S2

SUPPLEMENTARY FIGURE LEGENDS

SUPPLEMENTARY MATERIALS AND METHODS

Supplementary Figure S2

Supplementary Figure S3

Supplementary Figure S4

Supplementary Figure S5

Supplementary Figure S6

Supplementary Figure S7

Supplementary Figure S8

Supplementary Table S1
