# Correction: DNA breakpoint assay reveals a majority of gross duplications occur in tandem reducing VUS classifications in breast cancer predisposition genes

**DOI:** 10.1038/s41436-018-0276-1

**Published:** 2018-08-20

**Authors:** Marcy E. Richardson, Hansook Chong, Wenbo Mu, Blair R. Conner, Vickie Hsuan, Sara Willett, Stephanie Lam, Pei Tsai, Tina Pesaran, Adam C. Chamberlin, Min-Sun Park, Phillip Gray, Rachid Karam, Aaron Elliott

**Affiliations:** Department of Clinical Genomics, Ambry Genetics, 15 Argonaut Drive, Aliso Viejo, California 92656 USA

Correction to: *Genetics in Medicine*; 10.1038/s41436-018-0092-7; published online 28 July 2018.

The PDF and HTML versions of the article have been updated to include the Creative Commons Attribution 4.0 International License information.

