# Supplementary figures and images for: DNA breakpoint assay reveals a majority of gross duplications occur in tandem reducing VUS classifications in breast cancer predisposition genes
Source: Genet Med. 2018 Jul 28;21(3):683–93. doi: 10.1038/s41436-018-0092-7 (PMC6752314; doi:10.1038/s41436-018-0092-7)

Figure S1

a.

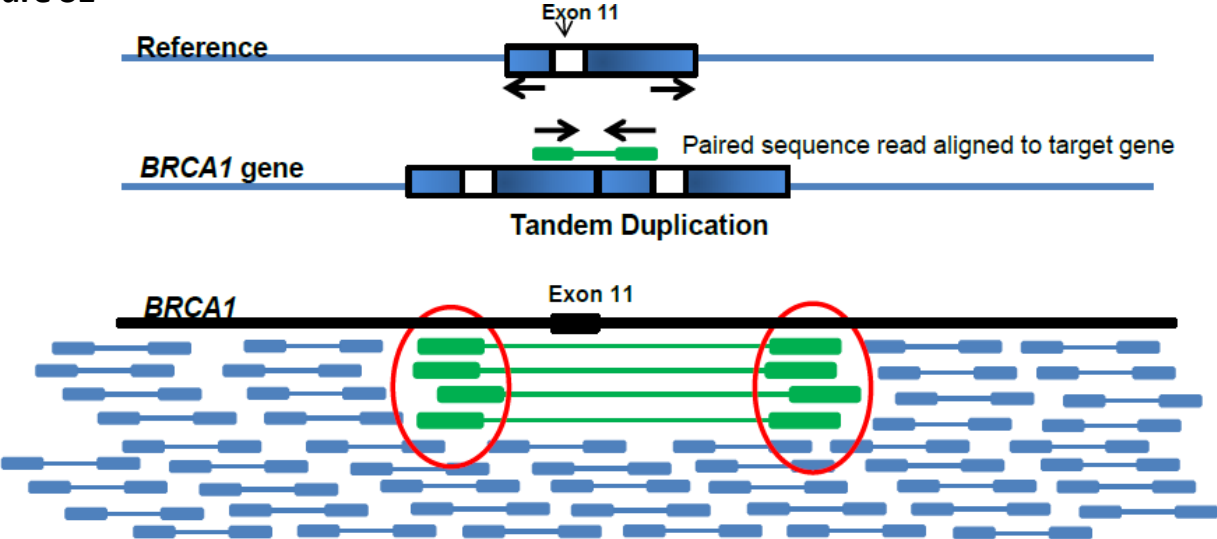

b.

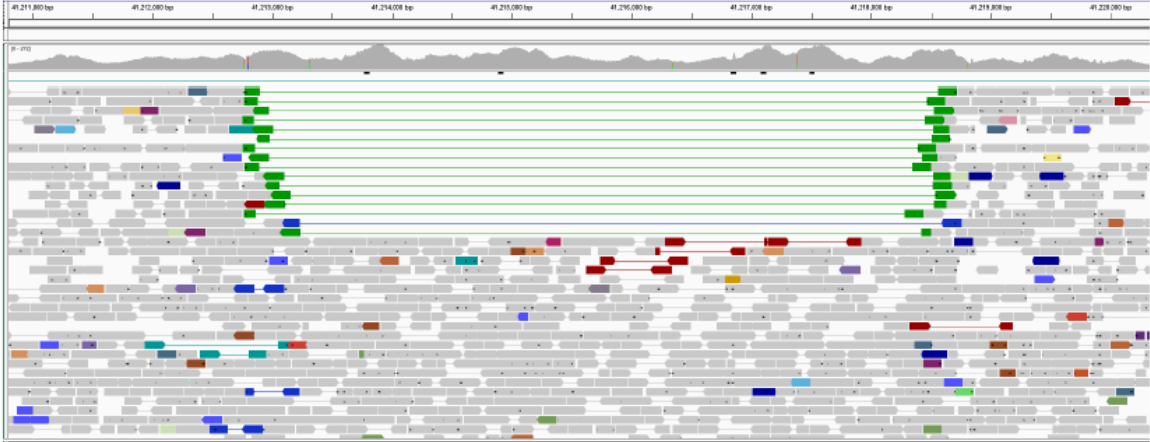

Supplement: Supplementary file 1 — Supplementary Figure S1 [file 41436_2018_92_MOESM1_ESM.pdf]

Figure S2

a.

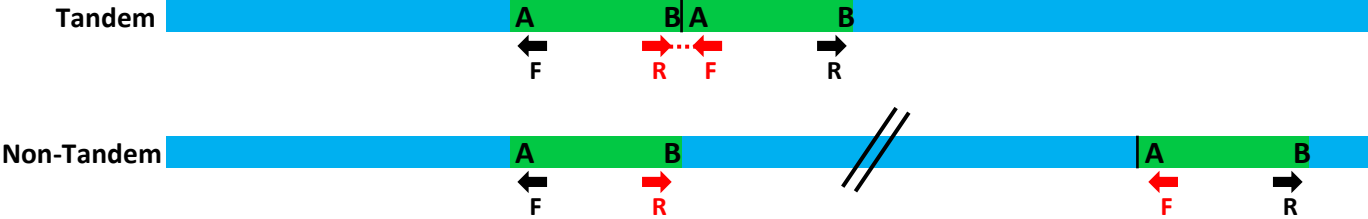

b.

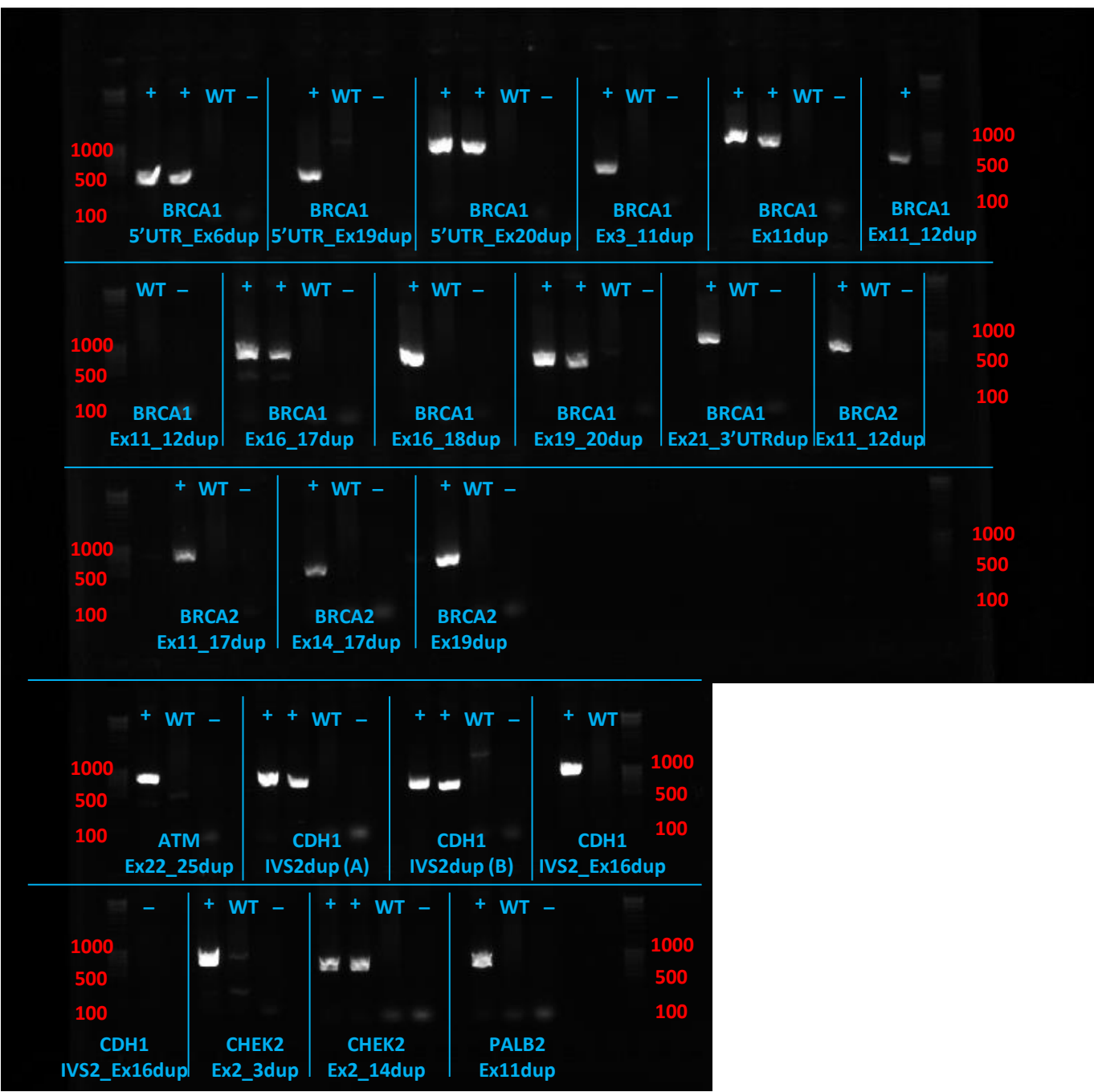

Supplement: Supplementary file 5 — Supplementary Figure S2 [file 41436_2018_92_MOESM5_ESM.pdf]

Figure S3

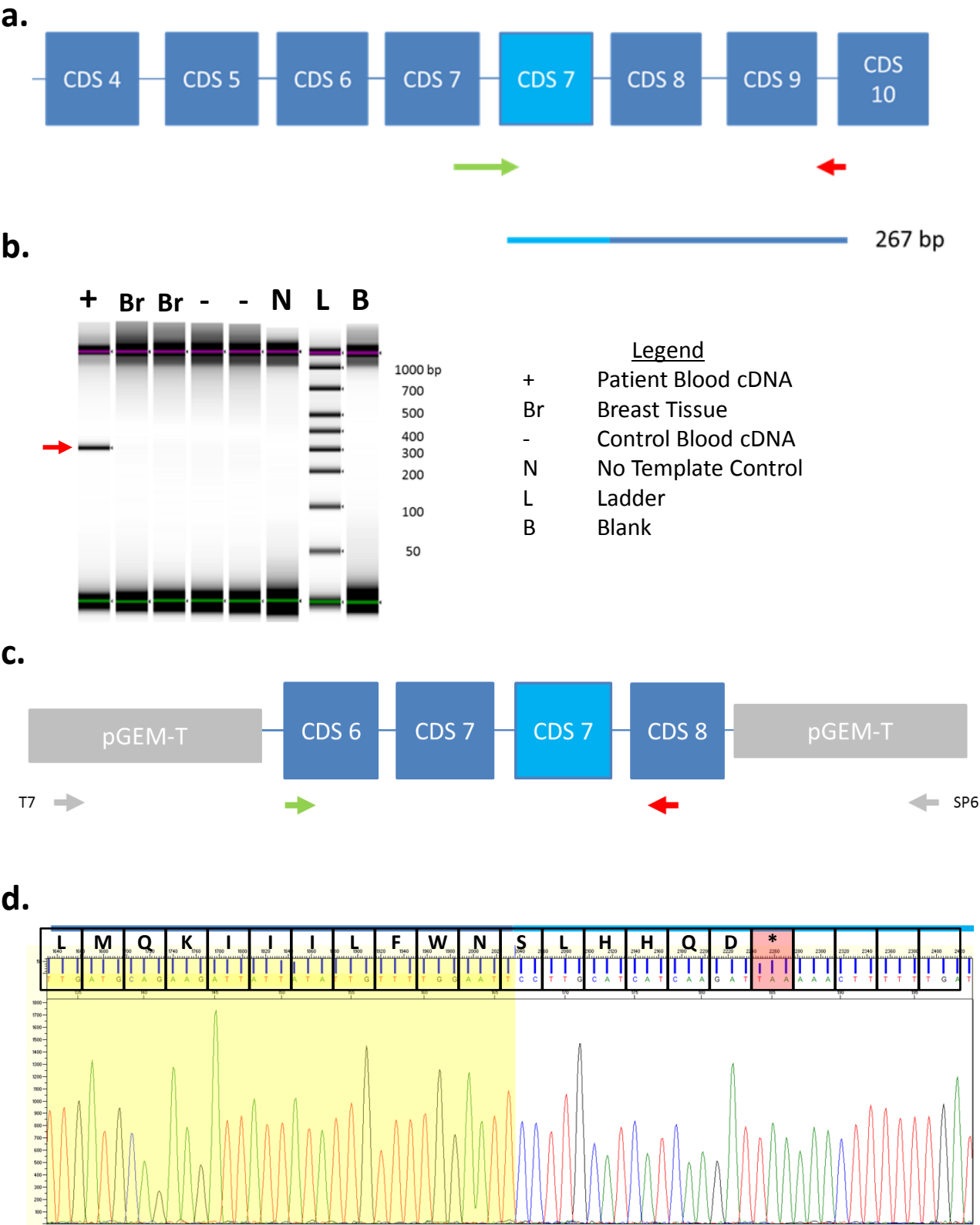

Supplement: Supplementary file 6 — Supplementary Figure S3 [file 41436_2018_92_MOESM6_ESM.pdf]

**Figure S4**

**5' Alu**

**3' Alu**

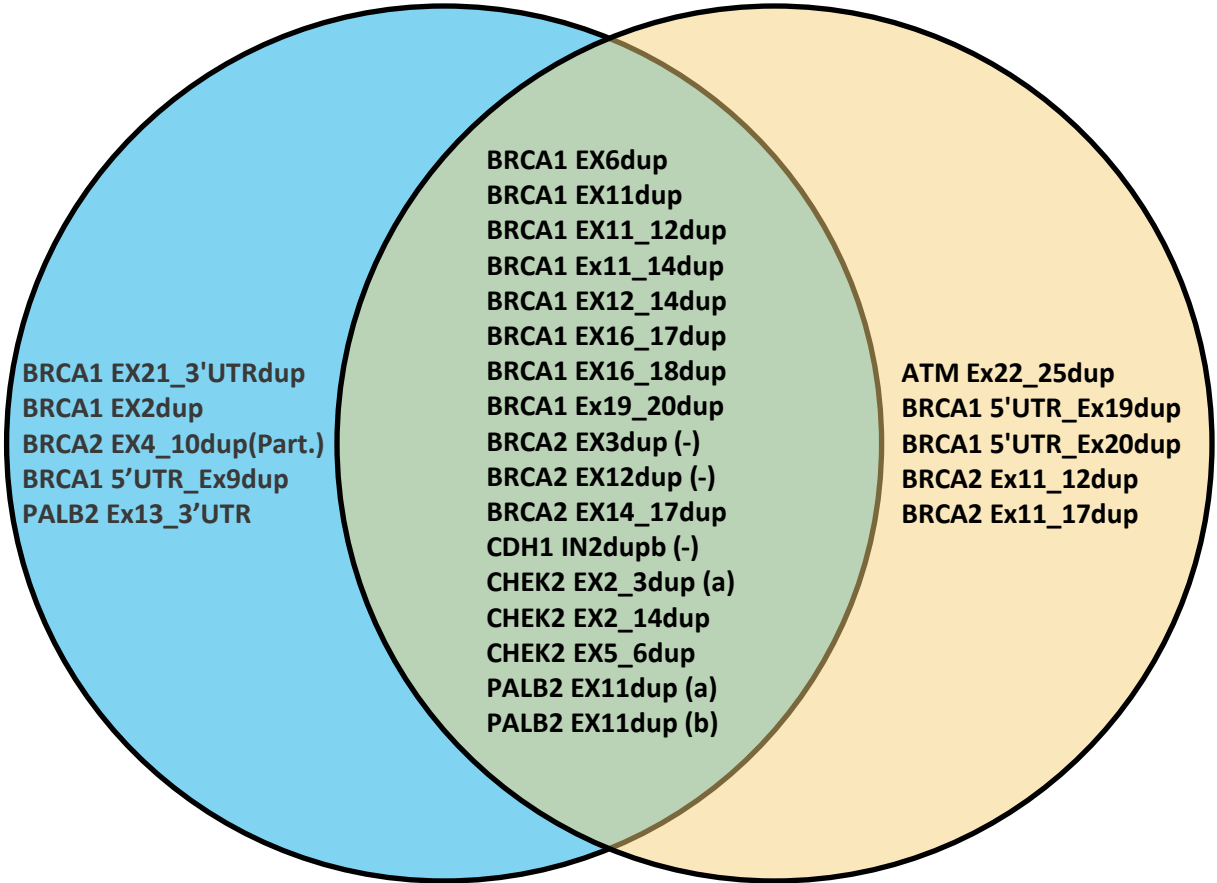

Supplement: Supplementary file 7 — Supplementary Figure S4 [file 41436_2018_92_MOESM7_ESM.pdf]

5'UTR 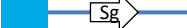

CDS1 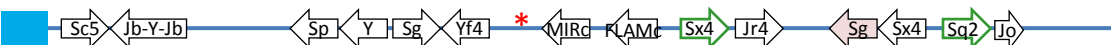

CDS2 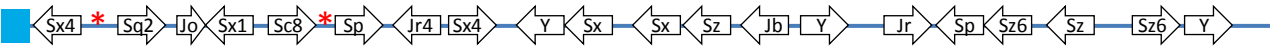

CDS3 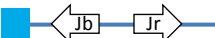

CDS4 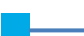

CDS5 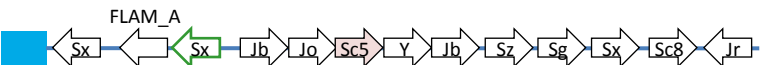

CDS6 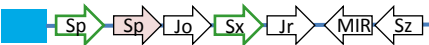

CDS7 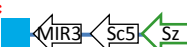

CDS8 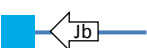

CDS9 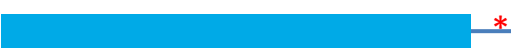

CDS10 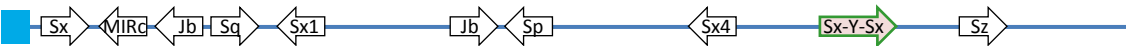

CDS11 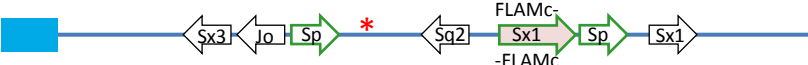

CDS12 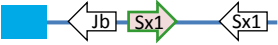

CDS13 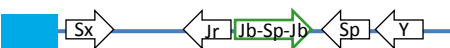

CDS14 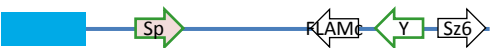

CDS15 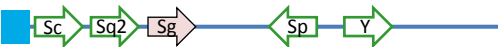

CDS16 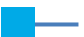

CDS17 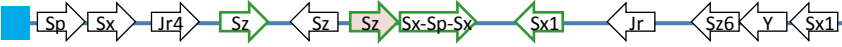

CDS18 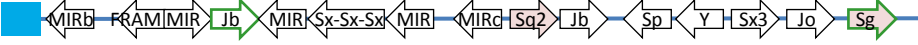

CDS19 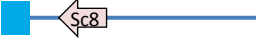

CDS20 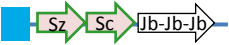

CDS21 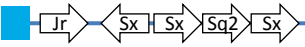

CDS22/  
3'UTR 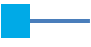

CDS22/  
3'UTR

Supplement: Supplementary file 8 — Supplementary Figure S5 [file 41436_2018_92_MOESM8_ESM.pdf]

Figure S7

a. *BRCA1* EX2dup

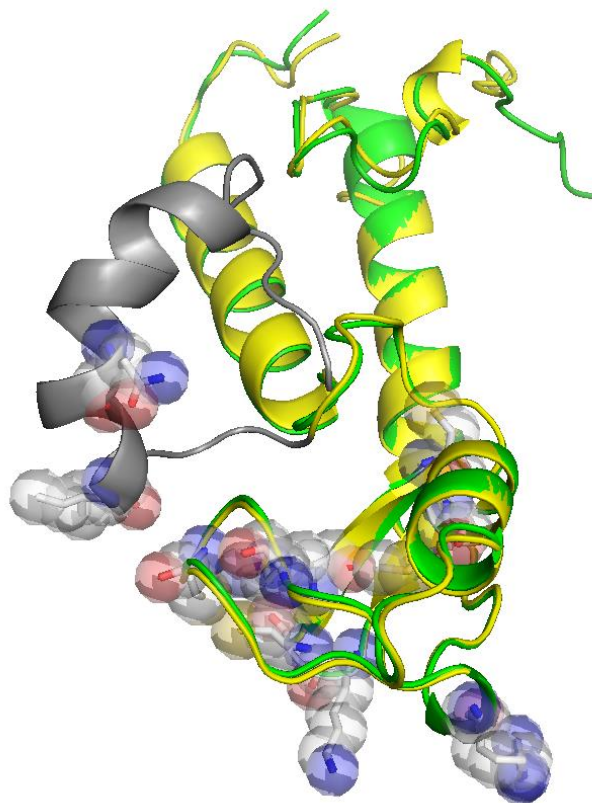

b. *BRCA1* EX19\_20dup

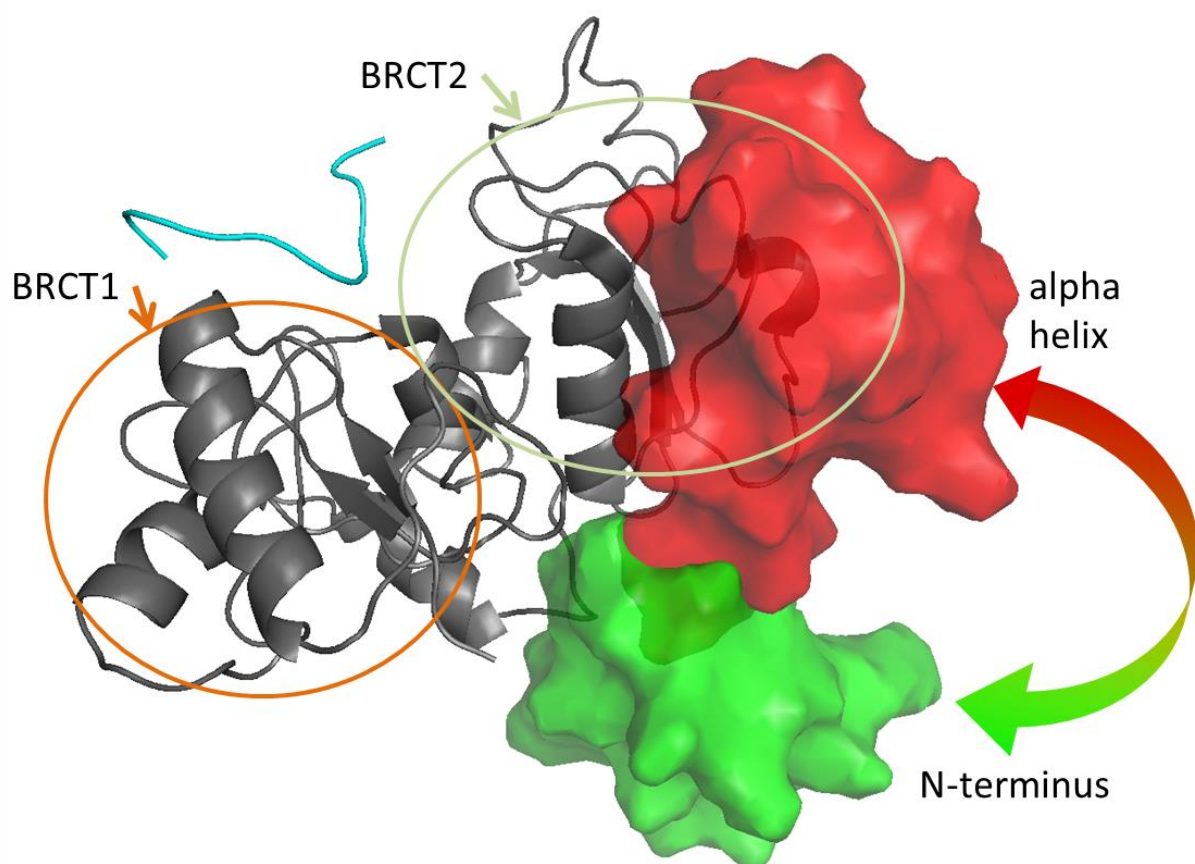

c. *CHEK2* EX2\_3dup

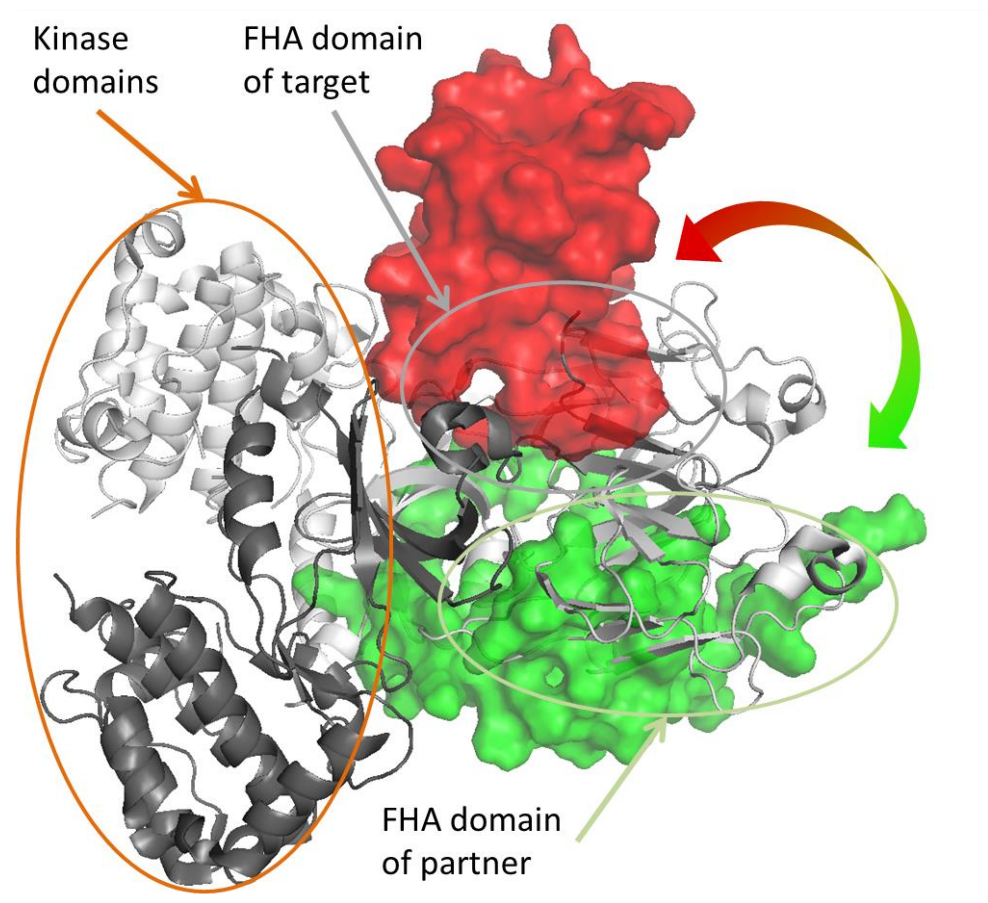

Supplement: Supplementary file 10 — Supplementary Figure S7 [file 41436_2018_92_MOESM10_ESM.pdf]
