# Supplementary material for: DNA breakpoint assay reveals a majority of gross duplications occur in tandem reducing VUS classifications in breast cancer predisposition genes
Source: Genet Med. 2018 Jul 28;21(3):683–93. doi: 10.1038/s41436-018-0092-7 (PMC6752314; doi:10.1038/s41436-018-0092-7)
Supplement: Supplementary file 2 — Supplementary Table S2 [file 41436_2018_92_MOESM2_ESM.pdf]

Table S2: Sample Failures

Gene	Coding Exon Nomenclature	Number of Samples Tested	Number of Samples Failed	Failure Reasons
ATM				
	EX16_60dup	3	3	Lack of Probe Coverage
	EX6_62dup	1	1	Low Quality Sample
	EX61_62dup	1	1	Pseudogene Interference
	EX61_3'UTRdup	13	13	Pseudogene Interference
BRCA1				
	5'UTR_EX1dup	4	1	Low Quality Sample
	5'UTR_EX9dup	2	1	Unknown
	EX12_14dup	2	1	Unknown
	EX12_15dup	1	1	Unknown
BRCA2				
	5'UTR_3'UTRdup	1	1	Lack of Probe Coverage
CDH1				
	EX16dup	1	1	Unknown
	EX3_EX16dup	2	1	Low Quality Sample
	5'UTR_3'UTRdup	3	3	Lack of Probe Coverage
CHEK2				
	EX2_14dup	35	1	Low Quality Sample
	5'UTR_3'UTRdup	1	1	Lack of Probe Coverage
	EX4dup	1	1	Low Quality Sample
	EX7dup	2	2	Lack of Probe Coverage
