# Supplementary material for: DNA breakpoint assay reveals a majority of gross duplications occur in tandem reducing VUS classifications in breast cancer predisposition genes
Source: Genet Med. 2018 Jul 28;21(3):683–93. doi: 10.1038/s41436-018-0092-7 (PMC6752314; doi:10.1038/s41436-018-0092-7)
Supplement: Supplementary file 3 — SUPPLEMENTARY FIGURE LEGENDS [file 41436_2018_92_MOESM3_ESM.docx]

**SUPPLEMENTARY FIGURE LEGENDS**

**Figure S1: DNA breakpoint assay design**. **(a)** A schematic diagram of hypothetical tandem *BRCA1* EX11dup depicting the custom designed NGS probes (black arrows) that cover deep intronic regions that are not normally covered by the standard NGS assay is shown. Paired-end reads that traverse the tandem duplication produce a read (green) that, when aligned back to the reference, show a large gap between them. **(b)**. An actual image of data produced by the DNA breakpoint assay resulting from paired-end reads (green) aligned back to the reference.

**Figure S2: PCR-based confirmation of tandem duplication. (a)** Graphic depicting logic and primer design for confirmation of tandem finding for a subset of gross duplications by PCR analysis. Forward and reverse primers are labeled with “F” and “R” and anneal to genomic locations “A” and “B”, respectively. Tandem duplications result in an amplicon that is represented by a dashed red line. Diagonal lines represent the break between the native gene and a duplication residing at a distal location. **(b)** Confirmation results from first-round, NGS-identified tandem breakpoints. WT is wildtype control; + is duplication carrier; - is no-template control.

**Figure S3**. **Structural analyses of in-frame gross duplications.** **(a)** BRCA1 EX2dup: The superimposed structures of the wildtype BRCA1 (green) and the BRCA1 EX2dup variant (yellow) are depicted. The duplicated inserted region is depicted in gray. The spheres and sticks depict the known internally classified pathogenic variants on the domain (with blue spheres representing nitrogen, red spheres representing oxygen and gray spheres representing oxygen molecules on the amino acid). **(b)** BRCA1 EX19_20dup: The tandem repeat structure of BRCT1 and BRCT2 (grey cartoon in orange and gray circles) in human BRCA1 with bound phosophopeptide (cyan cartoon). The range of plausible positions that the duplication might occupy are described by the insertion at alpha helix at the BRCT1 and BRCT2 domain interface (red surface) and at the N-terminal end of the duplicated region (green surface). **(c)** CHEK2 Ex2_3dup: The dimer structure of the CHEK2 protein with different positions of the duplicated protein is depicted. The altered monomer (dark grey cartoon) and its oligomer (light grey cartoon) are shown to emphasize the duplication’s overlap. The ranges of possible insertions of the duplicated region are shown for the N-terminal end (red surface) and the C-terminal end (green surface).

**Figure S4: Breakpoints that overlap with Alu elements**. A Venn diagram depicting alterations with at least one or both breakpoints in an Alu element. Alterations where breakpoints were disparate in multiple probands are designated with (a) or (b) after the designated gross duplication nomenclature to distinguish them from each other according to Table 1 and Table S1. For those in the overlapping region, both involved Alu elements were in the same orientation as gene transcription unless otherwise marked with (-). In all cases, the involved Alu elements were in the same orientation as each other.

**Figure S5:** **BRCA1 Alu elements**. Exon and intron structure of *BRCA1* is depicted roughly to scale. Alu elements are depicted by arrows corresponding to the orientation of the element with respect to *BRCA1*. Name, location and orientation of Alu elements were derived from UCSC Genome Browser build hg19 as imported by Repeat Masker (https://genome.ucsc.edu/cgi-bin/hgGateway accessed 8/11/17). Elements in which one or more breakpoints have been identified are shaded pink. Elements that were previously identified as being involved in gross duplication breakpoint are outlined in green^9^. Red asterisks depict the location of breakpoints that do not fall within an Alu element. Breakpoints that were in the 5’ or 3’ UTR are not depicted in this figure. Exon number is by coding exon rather than genomic exon. Asterisks indicate breakpoints identified that were not in an Alu repeat.

**Figure S6: Suggested flow chart for gross duplication classification.** Flow chart that considers the following information for classification of gross duplications: 1) tandem status; 2) classifiable (the alteration must be predicted to interrupt the coding sequence); 3) reading frame. Care must be taken to consider phenotype and the effect on clinically important functional domains which may involve a detailed structural analysis and evaluation of existing pathogenic mutations within the affected coding regions.

**Figure S7. Tandem RT-PCR identifies CHEK2 EX7dup as a tandem gross duplication. (a)** Schematic diagram of the cDNA from patient blood harboring tandem duplicated CHEK2 EX7dup with coding exons (CDS) 4-10 labeled in boxes. Duplicated CDS 7 is light blue. Primers designed to amplify the region of interest are indicated by arrows: green is forward and red is reverse. The expected amplicon is drawn beneath and the predicted size is labeled accordingly if the exon were inserted as a tandem duplication. **(b)** RT-PCR results from the design in part (a) show an amplicon of predicted size for a tandem duplication of CDS 7 migrating near the 300bp marker (red arrow) in the patient sample, only. **(c)** An 219 bp PCR product amplified from the patient cDNA using primers annealing to CDS 6 (green) and CDS 8 (red) was subcloned using pGEM-T vector and colonies carrying the same-sized insert were sequenced as depicted in this schematic. Both the vector-specific sequencing primers T7 and SP6 are shown with gray arrows. (gray) and the primers used for RT-PCR (forward CDS 6) and red (reverse-CDS 8) were used for sequencing. **(d)** Sanger sequencing results of part (c) using T7 primer show the junction of CDS 7 (yellow background) with tandem inserted duplicated CDS 7 (white background). The normal translational reading frame is indicated in black boxes around the codons and the encoded amino acid abbreviation is given within the codon box. The novel, in-frame stop codon is demarcated by * and the box is shaded red to emphasize the premature nature of this termination codon encoded by this tandem duplication. All other sequencing primers showed the same result (data not shown).

**Figure S8: Multiple sequence alignments of identified breakpoints.** The results of multiple sequence alignment by T-Coffee (http://www.ebi.ac.uk/Tools/msa/tcoffee/ Accessed 9/26/2017). Sequences where breakpoints fell within an Alu element (as reported by Repeat Masker) had the entire element selected for alignment. When sequences did not fall within an Alu element, sequences 100 nucleotides before and after the breakpoint were selected for alignment. The largest stretches of 100% sequence identity are outlined in red boxes. Red boxes with an arrowhead at one side have the 100% sequence identity carried-over from the line before or after.
