# Supplementary material for: DNA breakpoint assay reveals a majority of gross duplications occur in tandem reducing VUS classifications in breast cancer predisposition genes
Source: Genet Med. 2018 Jul 28;21(3):683–93. doi: 10.1038/s41436-018-0092-7 (PMC6752314; doi:10.1038/s41436-018-0092-7)
Supplement: Supplementary file 4 — SUPPLEMENTARY MATERIALS AND METHODS [file 41436_2018_92_MOESM4_ESM.docx]

**SUPPLEMENTARY MATERIALS AND METHODS**

**Breakpoint PCR Confirmation**

PCR Primers were designed around the breakpoint to amplify the breakpoint junction in the presence of a duplication event. Breakpoint junctions were amplified under the following conditions: 35 cycles of 95°C for 30 seconds, 61° for 30 seconds, and 72°C for 45 seconds, followed by a final extension step at 72°C for 10 minutes, 0.5uM forward and reverse primer, HotstarTaq (Qiagen) with 1.5mM MgCl_2_. PCR products were assessed by gel electrophoresis on a 1.5% agarose gel and visualized by GelRed staining (Biotium, CA).

**Structural Analysis**

Visualizations were performed using Pymol (The PyMOL Molecular Graphics System, Version 1.8 Schrödinger, LLC.). The models were constructed using Rosetta with comparative modeling or remodel protocols^1, 2^, templating a known x-ray crystal structure of the BRCA1 RING domain for BRCA1 EX2dup (PDB id: 1JM7)^3^, or a homology model off of known crystal structures (BRCA1:4JLU^4^ for BRCA1 EX19_20dup; and CHEK2:3I6U^5^ for CHEK2 EX2_3dup) of the relevant region with an inserted loop representing the region of the duplication that is misfolded. The loop region was independently folded using AbinitioRelax,^6^ was subsequently stitched on the homology model at the point of insertion and finally relaxed using kinematic closure (KIC)^7^. The region of insertion to be treated as a loop was initially guessed using TCoffee alignment program with the structural comparison flag turned on^8^. In both CHEK2 and BRCA1 the alignment inserted the loop region in the middle of the duplicated region, causing the disordered loop to appear as part of a structural feature in the domain (alpha helix 2 in the BRCT2 domain of BRCA1 and the XX hairpin turn of CHEK2). Alternatively the 1^st^ instance (N-terminal insertion) or the 2^nd^ instance (C-terminal insertion) of the duplication was treated as the loop region.

**RT PCR**

PAXgene tubes containing whole blood were submitted via mail at room temperature then incubated at -20°C for 24 hours before being transferred to long-term storage at -80°C for a maximum of 6 months. Total RNA was isolated following manufacturer’s guidelines (PAXgene, Qiagen). Isolated RNA quality and quantity was determined using TapeStation HSRNA (Agilent). Sense and antisense primers were designed on refseq cDNA (*CHEK2* NM_007194). Primers were designed so that the sense primer spanned the abnormal exon junction, thereby requiring a tandem duplication to create an amplicon (Tandem RT-PCR). Primers were also designed in exons flanking the duplicated region (RT-PCR). All primers were designed with similar thermodynamic properties. Total RNA was used to synthesize cDNA with a 500ng input using OligodT (SuperScript IV, Invitrogen). Both primer sets were run under the same conditions (500ng cDNA, 0.5uM primer, HotstarTaq with 1.5mM MgCl_2_, 35 cycles with 45s elongation). Annealing temperature was optimized to the Tandem RT-PCR amplicon. The patient, three WT blood controls, two normal breast tissue controls, and a non-template control were all assayed using the same master mix. Amplicons were visualized and quantified using TapeStation D1000 (Aglient). RT-PCR product from the patient was cleaned and concentrated to remove enzymes with exonuclease activity, then A-tailed and ligated to pGEM-T plasmid (Promega). Subsequently, competent *E.Coli* cells were transformed using 12ng of ligated RT-PCR product and plated using Ampicillin resistance for selection (Promega). Colonies were screened for the insert of interest using RT-PCR primers under same PCR conditions as above using 1ul of plated bacteria as template (Colony PCR). Sanger sequencing was performed on colonies containing the insert of interest to confirm presence of abnormal exon junctions using T7 and SP6 primers specific to pGEM-T (Genewiz). Colony PCR product was also Sanger sequenced using the RT-PCR primers (20ng Colony PCR product, 2.5uM primer; Genewiz). Resulting electropherograms were visualized using Sequence Scanner (Applied Biosciences).

**SUPPLEMENTARY REFERENCES**

**1.** Chivian D, Baker D. Homology modeling using parametric alignment ensemble generation with consensus and energy-based model selection. *Nucleic Acids Res* 2006;34(17):e112.

**2.** Huang PS, Ban YE, Richter F, et al. RosettaRemodel: a generalized framework for flexible backbone protein design. *PLoS One* 2011;6(8):e24109.

**3.** Brzovic PS, Rajagopal P, Hoyt DW, King MC, Klevit RE. Structure of a BRCA1-BARD1 heterodimeric RING-RING complex. *Nat Struct Biol* Oct 2001;8(10):833-837.

**4.** Badgujar D, Varma AK. *Crystal structure of BRCA1 BRCT with doubly phosphorylated Abraxas*: <http://www.rcsb.org/structure/4JLU>; 2013.

**5.** Cai Z, Chehab NH, Pavletich NP. Structure and activation mechanism of the CHK2 DNA damage checkpoint kinase. *Mol Cell* Sep 24 2009;35(6):818-829.

**6.** Raman S, Vernon R, Thompson J, et al. Structure prediction for CASP8 with all-atom refinement using Rosetta. *Proteins* 2009;77 Suppl 9:89-99.

**7.** Mandell DJ, Coutsias EA, Kortemme T. Sub-angstrom accuracy in protein loop reconstruction by robotics-inspired conformational sampling. *Nat Methods* Aug 2009;6(8):551-552.

**8.** Notredame C, Higgins DG, Heringa J. T-Coffee: A novel method for fast and accurate multiple sequence alignment. *J Mol Biol* Sep 8 2000;302(1):205-217.
