# Supplementary material for: DNA breakpoint assay reveals a majority of gross duplications occur in tandem reducing VUS classifications in breast cancer predisposition genes
Source: Genet Med. 2018 Jul 28;21(3):683–93. doi: 10.1038/s41436-018-0092-7 (PMC6752314; doi:10.1038/s41436-018-0092-7)
Supplement: Supplementary file 9 — Supplementary Figure S6 [file 41436_2018_92_MOESM9_ESM.pdf]

Figure S6

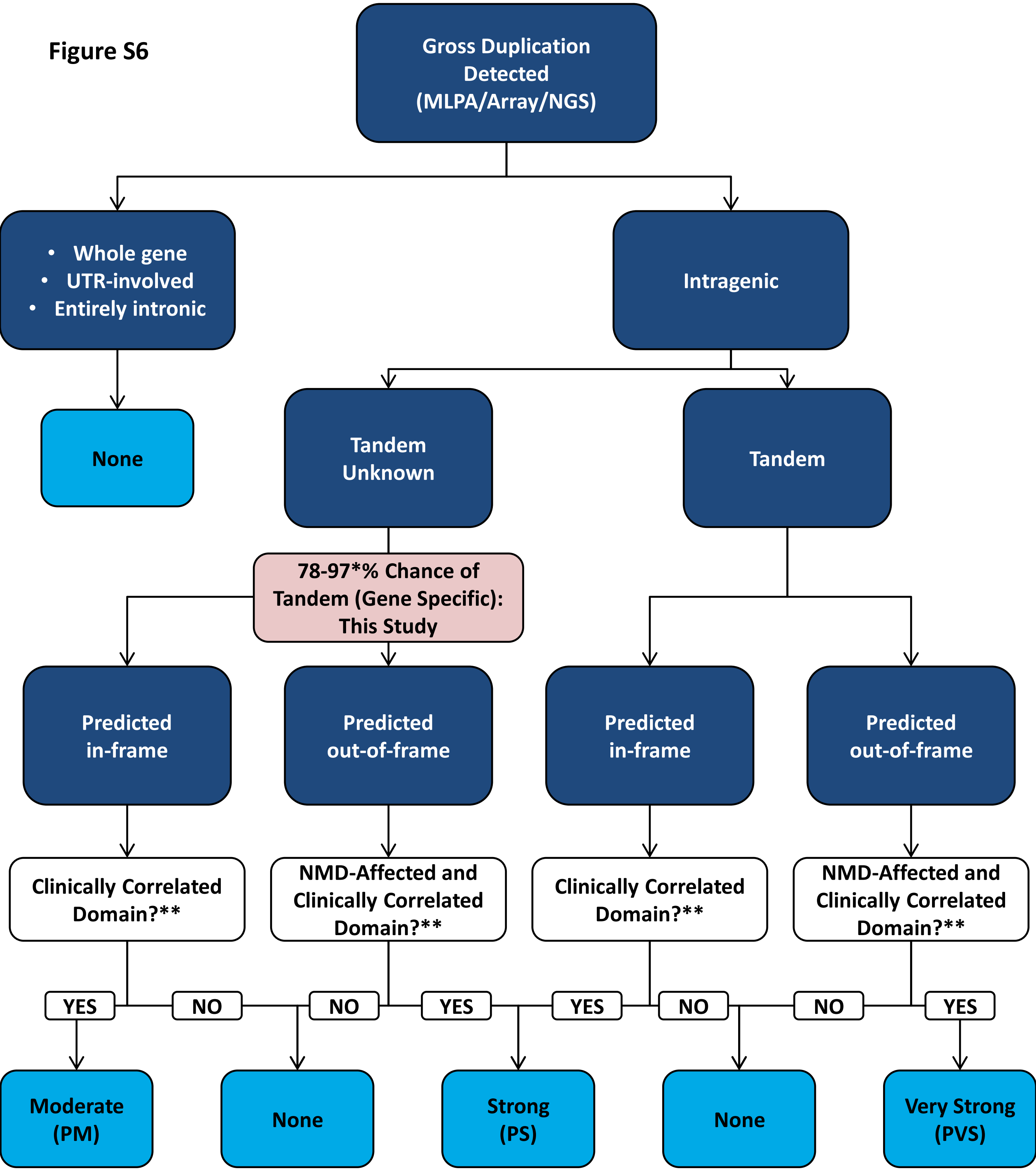

* Percent chance of tandem: lower and upper % is based on respective inclusion or exclusion of samples with known technical (versus unknown) failures

** Clinically Correlated Domains:

- Disease specific functional importance
- Structural importance
- Harbors known missense pathogenic mutations
- Not contained within a naturally occurring isoform
