# Supplementary material for: DNA breakpoint assay reveals a majority of gross duplications occur in tandem reducing VUS classifications in breast cancer predisposition genes
Source: Genet Med. 2018 Jul 28;21(3):683–93. doi: 10.1038/s41436-018-0092-7 (PMC6752314; doi:10.1038/s41436-018-0092-7)
Supplement: Supplementary file 11 — Supplementary Figure S8 [file 41436_2018_92_MOESM11_ESM.pdf]

Figure S8

BRCA1

```
CLUSTAL W (1.83) multiple sequence alignment

ATM-Ex22_25dup-3_  -------TTTTGGTTAAAGATGTAAGTTAAATTCTTGGCCGGGTGCGGTG
ATM-Ex22_25dup-5_  AGAGGCAGTCTGGCTACAGT--------GGCTTTGTGGCACTATGTTGGG
                           * *** ** **            **  ****    **  * *

ATM-Ex22_25dup-3_  GCTAACG---CCTGTAATCCCAGCACTTTGGGAG---GC--CAAGGCGGG
ATM-Ex22_25dup-5_  CTCCACCCAGTTCGAACTTCCTACTGGCTGTGTTTATACTGTGAGGGGAA
                       **       * * * **  *    ** *      *    *** *

ATM-Ex22_25dup-3_  TGGATC-ACCTGAG--ATCAGGAGTTCAAGACCAACCTGGCCAACATGGT
ATM-Ex22_25dup-5_  AACATCCTACTGAAGCCTCAGTAATTGCAGACGCCCCTCCCC-CCAC---
                      ***   ****    **** * **  ****   ***  **  **

ATM-Ex22_25dup-3_  GAAACCCCATCTCTACTGGGTGTGGTGGTGTGTGCCTGTAATCCCAGCTG
ATM-Ex22_25dup-5_  CAAGCTTCAG-TGTCCCAGGTC----GACTTCAGACTGTTCTGCTGGCAG
                    ** *  **  * * *  ***     *   *  * ****  * *  ** *

ATM-Ex22_25dup-3_  TTTGGGAAGCT-GAGTCAG
ATM-Ex22_25dup-5_  C--GAGAATTTCAAGCCAG
                      * ***  *  ** ***
```

```
CLUSTAL W (1.83) multiple sequence alignment

ATM-Ex22_25dup-3__RC_  CTGACTCAGCTTCCCAAACAGCTGGGATTACAGGCACA-CA--CCACCAC
ATM-Ex22_25dup-5_      -AGAGGCAGTCTGGCT-ACAG-TGGC-TTTGTGGCACTATGTTGGGCTCC
                         **  ***  *  *  **** ***  **   *****         *  *

ATM-Ex22_25dup-3__RC_  ACCCAGTA-GAGATGGGGTTTCACCATGTTGGCCAGGTTGGTCTTGAA--
ATM-Ex22_25dup-5_      ACCCAGTTCGAACTTCCTACTGGCTGTGTTTATACTGTGAGGGGAAAACA
                       *******  **  *      *  *  ****      **  *     **

ATM-Ex22_25dup-3__RC_  --CTCCTGA--TCTCAGGTGATCCACCCGCCTTG-GCCTCCCAAAGTGCT
ATM-Ex22_25dup-5_      TCCTACTGAAGCCTCAGTAATTGCAGACGCCCCTCCCCCCACCAAGCTTC
                         ** ****   *****    * **  ****     ** * * ***

ATM-Ex22_25dup-3__RC_  GGGATTACAGGCGTTAGCCACCGCACCCGGCCAAGAATTTAACTTACATC
ATM-Ex22_25dup-5_      AGTGTCCCAGGT-----CGACTTCAGACTGTTCTGCTGGCAGCG-AGAAT
                        *  *  ****      * **  **  * *    *     * *  * *

ATM-Ex22_25dup-3__RC_  TTTAACCAAAA
ATM-Ex22_25dup-5_      TTCAAGCC-AG
                       ** ** *  *
```

```
CLUSTAL W (1.83) multiple sequence alignment

BRCA1-5_UTRdup-3_  ----CTGGGAAAGGTTA-G--GGGCGGGGAGG-CCTTGATTGGTGTTGG-
BRCA1-5_UTRdup-5_  GCTATTGAGAAAGCGCAAGAGGGAAGTAGAGGAGCGTCAGTAGTAACAGA
                        ** *****   * *  **  *  ****  * * * * **    *

BRCA1-5_UTRdup-3_  TTTGGTCGTTGTTGATTTTGGTTTTATGCAAGAAAAAGAAAACAACCAGA
BRCA1-5_UTRdup-5_  TGCTGCCGGCAGGGATG-TG-CTTGA-GGGGGATCCTGAGATGAGAGTGG
                   *   * **     ***  **  ** * *   **    ** *  *    *

BRCA1-5_UTRdup-3_  AACATTGGAGAAAGCTAAGGCTACCACCACCT-ACCCGG--TCAGTCACT
BRCA1-5_UTRdup-5_  GTCGCTGGGAAAGGCTAGGGGCAGGGAGGCCTTGATTGGTGTTGGTTTGG
                     *  ***  ** **** **  *      ***     **  *  **

BRCA1-5_UTRdup-3_  CCTCTGTAGCTTTCTCTTTCTTGGAGAAAGGAAAAGACCCAAGGGGTTGG
BRCA1-5_UTRdup-5_  TCGTTGTTGATTTTGGTTTTATGC---AAGAAAAAGA---AAACA---GC
                    *  *** * ***   ***  **    *** ******   **      *

BRCA1-5_UTRdup-3_  CAGCAATATGTG
BRCA1-5_UTRdup-5_  CAGAAGCATTGG
                   *** *  **  *
```

```
CLUSTAL W (1.83) multiple sequence alignment

BRCA1-5_UTRdup-3_-RC  CACATATTGCTGCCAACCCCT-TGGGTCTTTTCCTTTCTCCA--AGAAAG
BRCA1-5_UTRdup-5_     --GCTATTGA-GAAAGCGCAAGAGGGAAGTAGAGGAGCGTCAGTAGTAAC
                          *****  *  * * *    ***   *       *  **  ** **

BRCA1-5_UTRdup-3_-RC  AGAAAGCT----ACAGAGGAGTGACTGACCGGGTAGGTGGTGGTAGCCTT
BRCA1-5_UTRdup-5_     AGAT-GCTGCCGGCAGGGATGTGCTTGAGGGGGATCCTGA-GATGAGAGT
                      ***  ***     *** *  ***  ***  ***    **  * *     *

BRCA1-5_UTRdup-3_-RC  AGCTTTCTC--CAATGTTTCTGG-----TTGTTTTC-TTTTTCTTGCATA
BRCA1-5_UTRdup-5_     GGGTCGCTGGGAAAGGCTAGGGGCAGGGAGGCCTTGATTGGTGTTGGTTT
                       * *  **    ** * *   **       *  **  **  * ***  *

BRCA1-5_UTRdup-3_-RC  AAACCAAAATCAACAACGACCAAACCAACACCAATCAAGGCCTCCCCGCC
BRCA1-5_UTRdup-5_     GGTCGTTGTTGATTTTGGTTTTATGCAAGAAAAAGAAA-A-----CAGC-
                         *     * *     *    *  *** *  **  **       * **

BRCA1-5_UTRdup-3_-RC  CCTAACCTTTCCCAG
BRCA1-5_UTRdup-5_     CAGAAGCATT---GG
                      *  ** * **    *
```

```
CLUSTAL W (1.83) multiple sequence alignment

BRCA1-5_UTR_Ex1dup-3_  TTTACATATCGGAGTTTTCATCTGTTCAAAGTTTGTGTTTACAGTGTGTT
BRCA1-5_UTR_Ex1dup-5_  -----------------------------------------------GTT
                                                                      ***

BRCA1-5_UTR_Ex1dup-3_  TATATAGTTTAGATTATAATTACCATACTGAAATATAATTGTTTCAGAAT
BRCA1-5_UTR_Ex1dup-5_  TATATAGTGTAGATTATAATTACCATACTGAAATATAATTATTTCAGAAT
                       ******** ******************************* *********

BRCA1-5_UTR_Ex1dup-3_  TGAGTCAGTGGTGAGAATGAAAGCCATCTGGTATGATAACTGAATCCAAT
BRCA1-5_UTR_Ex1dup-5_  TGAGTCAGTGGTGAGAATGAAAGCCATCTGGTATGATAACTGAATCCAAT
                       **************************************************

BRCA1-5_UTR_Ex1dup-3_  TTTTCTTTTACGGAGAATTTCTTTGAAATGTAGCTTATCTCAGAAATAGG
BRCA1-5_UTR_Ex1dup-5_  TTTTCTTTTACGGAGAATTTCTTTGAAATGTAGCTTATCTCAGAAATAGG
                       **************************************************

BRCA1-5_UTR_Ex1dup-3_  GATTTAGTAACCAATCAGAGTTTTCTTTGTCAAGGTTGTTTTTCTTTTTA
BRCA1-5_UTR_Ex1dup-5_  GATTTAGTAACCAATCAGAGTTTTCTTTGTCAAGGTTGTTTTTCTTTTTA
                       **************************************************

BRCA1-5_UTR_Ex1dup-3_  AAGTCACA----T
BRCA1-5_UTR_Ex1dup-5_  AAGTCACATTTGG
                       ********
```

Figure S8

BRCA1

```
CLUSTAL W (1.83) multiple sequence alignment

BRCA1-5_UTR_Ex6dup-3_   GAATATCTGTTTTTCAACAAGTACA---TTTTTTTAACCCTTTTAATTAA
BRCA1-5_UTR_Ex6dup-5_   -----TGCGT---TGAAGAAGTACAAAATGTCCTTAATGCTAT---GCAG
                             *  **   * ** *******   * *  ****  ** *     *

BRCA1-5_UTR_Ex6dup-3_   GAAAACTTTTATTGATTTATTTTTTGGG----GGGAA--ATTTTTTAGGA
BRCA1-5_UTR_Ex6dup-5_   AAAATCTTACAGTGTCCAATCTGGTAAGTCACCAGAAGAGGGTATTAATT
                         *** ***  * **    ** *  *  *      ***      * ***

BRCA1-5_UTR_Ex6dup-3_   TCTGATTCTTC--TGAAGATACCGTTAATAAGG-----CAACTTAT--TG
BRCA1-5_UTR_Ex6dup-5_   TGGGATTCCTATATGATTATCTCCTATGCAAATGAACAGAATTGACCTTA
                        *  ***** *   ***  **  * *    **         ** * *   *

BRCA1-5_UTR_Ex6dup-3_   CAGGTGAGTCAAAGAGAACCTTTGTCTATGAAGCTGGTATTTTCCTATTT
BRCA1-5_UTR_Ex6dup-5_   CATA-GAAGGGAGGAAAAGACATGTCTAATAAGATTAGGCTATTGTAATT
                        **   **    * ** **    ******  *** *     * *  ** **

BRCA1-5_UTR_Ex6dup-3_   AGT--TAATATTAAGGATTG
BRCA1-5_UTR_Ex6dup-5_   GCTGATTTTCTTAACTGAAG
                          *  *  * ****     *
```

```
CLUSTAL W (1.83) multiple sequence alignment

BRCA1-5_UTR_Ex6dup-3_-RC  CAATCCTTAATATTAACTAAATAG-GAAAATACCAGCTTCATAGAC--AA
BRCA1-5_UTR_Ex6dup-5_     -TGCGTTGAAGAAGTACAAAATGTCCTTAATGCTATGCAGAAAATCTTAC
                                * ** *   ** ****      *** * *     * *  *  *

BRCA1-5_UTR_Ex6dup-3_-RC  AG-GTTCTCTTT---GACTCACCTGCAATAAGTTGCCTTATTAACGGTAT
BRCA1-5_UTR_Ex6dup-5_     AGTGTCCAATCTGGTAAGTCACCAG-AAGAGGGTA-TTAATTTGGGATTC
                          ** ** *  * *    * ***** * ** * * *   * ***   * *

BRCA1-5_UTR_Ex6dup-3_-RC  CTTCAGAAGAATC--AGATCCTAAAA----AATTTCCCCCCAAAAAATAA
BRCA1-5_UTR_Ex6dup-5_     CTATATGATTATCTCCTATGCAAATGAACAGAATTGA-CCTTACATAGAA
                          **  *  *  ***    ** * **      * **   **  * * * **

BRCA1-5_UTR_Ex6dup-3_-RC  ATCAATAAAAGTTTTCTTAATTAAAAGGGTTAAAAAAATGTACTTGTTGA
BRCA1-5_UTR_Ex6dup-5_     GGGAGGAAAAGACATGT---CTAATAAGATTAGGCTATTGTAATTGCTGA
                             *  *****   * *    *** * * ***    * **** *** ***

BRCA1-5_UTR_Ex6dup-3_-RC  AAAACA--GATATTC
BRCA1-5_UTR_Ex6dup-5_     TTTTCTTAACTGAAG
                              *     *
```

```
CLUSTAL W (1.83) multiple sequence alignment

BRCA1-5_UTR_Ex19dup-3_   TTTTTTGAGGTCGAGTCTCTGTTGCCCAGGCCAGAGTGC--AATGGC-GC
BRCA1-5_UTR_Ex19dup-5_   AA-ATGCAAGTTGTGTTTTCAGAG-AGATACAAGATTTATAAATAACCAT
                             *  * ** * ** *     *   *  * *** *    ***   *

BRCA1-5_UTR_Ex19dup-3_   GATCTCGGCT-CACTGCAACCT-CCACCTC-CCGGGTTCAAGTGATTCTC
BRCA1-5_UTR_Ex19dup-5_   CCTCTCCCTTGCCCTTCAACATTTAGCTAAACAAAAACAAGAGGAAAAC
                           ****   * * ** **** *   * **   *     **** *     *

BRCA1-5_UTR_Ex19dup-3_   CTGCCT--CAGCCTCCCAAATAGCTGAGACTACAGGCGTGTGCCACCACG
BRCA1-5_UTR_Ex19dup-5_   AAGATTTACAATTTATCAATTCATTGAAAATCAGAGCCAGAGA-AGCAGG
                           *  *  **   *  *** *   *** * *    **  * *  * ** *

BRCA1-5_UTR_Ex19dup-3_   C-CCGACTAATTTTTTGTAT-TTTTTTTTTAGTAGAGACGGGGTTTCACC
BRCA1-5_UTR_Ex19dup-5_   AAATGAC--ATTGTAGGAAAAAACTGCTTTTGAAAAAGCACAACTTAC--
                             ***  *** *  * *    *  *** * * *  *     **

BRCA1-5_UTR_Ex19dup-3_   GTGTTAGCCA
BRCA1-5_UTR_Ex19dup-5_   ---TCATGAC
                            * *
```

```
CLUSTAL W (1.83) multiple sequence alignment

BRCA1-5_UTR_Ex19dup-3_-RC  TGGCTAACACGGTGAAACCCCGTCTCTACTAAAA-AAAA--AATACAAAA
BRCA1-5_UTR_Ex19dup-5_     ----AAATGC----AAGTTGTGTTTCAGAGAGATACAAGATTTATAAAT
                               **  *    **     ** *  *   * * * **    ** ***

BRCA1-5_UTR_Ex19dup-3_-RC  AATTAGTCGGGC-GTGGTGGCACACGCCTGTAGTCTCAGCTATTTGGGAG
BRCA1-5_UTR_Ex19dup-5_     AACCATCCTCTCCCTTGCCCTTCAAC------ATTTAGCTAAACAAAAA
                           **  *  *   *  * *    **        * * *****     *

BRCA1-5_UTR_Ex19dup-3_-RC  GCTGAGGCAG--GAGAATCAC--TTGAACCCGGGAGGTGGAGGTT----G
BRCA1-5_UTR_Ex19dup-5_     CAAGAGGAAAACAAGATTTACAATTTATC-AATTCATTGAAAATCAGAGC
                              **** *    *** * **  ** * *        ** *  *

BRCA1-5_UTR_Ex19dup-3_-RC  CAGTGAGCCGAGATCGCGCCATTGCA------CTCTGGCCTGGGCAACAG
BRCA1-5_UTR_Ex19dup-5_     CAGAGAAGCAGGA-AATGACATTGTAGGAAAAACTGCTTT-TGAAAAAG
                           *** **  *  **    * ***** *      ***    *  * ** **

BRCA1-5_UTR_Ex19dup-3_-RC  -AGACTCGACCTCAAAAAA
BRCA1-5_UTR_Ex19dup-5_     CACA-ACTTACTCA-TGAC
                            * *  *   ****   *
```

```
CLUSTAL W (1.83) multiple sequence alignment

BRCA1-5_UTR_Ex20dup-3_   GGCGAAACCCCGTCTCTACTAAAAATACAAAAATTAGCTGGGCGTAGGGG
BRCA1-5_UTR_Ex20dup-5_   CCCCA-GTGCCACGGGGGCTGCAGAGCTGGACCTGATCTT-CCTTCGAGG
                           * *    **        **  * *     *  * * **   * * * **

BRCA1-5_UTR_Ex20dup-3_   CGCACGCCTGTAGTCCCAGCTACTTGAGGGGCTGGGGCAGGAGAATCGCT
BRCA1-5_UTR_Ex20dup-5_   CAT-TATGGAAAGTCCCA--TAGTAAGATCCCTGGCCAAGGTACAGTGCT
                         *         *******  ** *        ****   ***    *  ***

BRCA1-5_UTR_Ex20dup-3_   TGAACCCCGGAGGCGGAGGTTGCAGTGAGCCAAGATCGCGCCACTGCA--
BRCA1-5_UTR_Ex20dup-5_   CCAGGGAGGTGGGCACAAG-GGCACTGTGCTTGGGGA--GGCACAGTAGG
                           *     *  ***  * *  *** ** **    *     * *** * *

BRCA1-5_UTR_Ex20dup-3_   --CTCCAGCCCGGGCGACAGAGC-----GAGACTCCGTCTCAAAAAAAAA
BRCA1-5_UTR_Ex20dup-5_   GGATGGGGTATTAGCTACAGAGGGCTTTGGGAATGGGACATGTGGCACAA
                            *   *     ** ******      * ** *  * *       * **

BRCA1-5_UTR_Ex20dup-3_   GAGAGAGAG
BRCA1-5_UTR_Ex20dup-5_   GAGGCCCCC
                         ***
```

```
CLUSTAL W (1.83) multiple sequence alignment

BRCA1-5_UTR_Ex20dup-3_-RC  CT-CTCTCTCTTTTTT-TTTGAGACGGAGTCTCGCTCTGTCGCCCGGGC-
BRCA1-5_UTR_Ex20dup-5_     CCCCAGTGCCACGGGGGCTGCAGAGCTGGACCTGATCTTCCTTCGAGGCA
                           *  *  *  *        *  ***    * *  * ***  *  *  ***

BRCA1-5_UTR_Ex20dup-3_-RC  ---TGG--AGTGCAGTGGCGCGATCTTGGCTCACTGCAACCTCCGCCTCC
BRCA1-5_UTR_Ex20dup-5_     TTATGGAAAGTCCCATAGTAAGATCCCTGGCCAAGGTACAGTGCTCCAGG
                              ***  *** *  * *   ****   *  **  * *   * * **

BRCA1-5_UTR_Ex20dup-3_-RC  G----GGGTTCAAGCGATTCTCCTGCCCCAGCCCCTCAAGTAGCTGGGAC
BRCA1-5_UTR_Ex20dup-5_     GAGGTGGGCACAAGG----------GCACTGTGCTTGGGG----AGGCAC
                           *    ***  ****           * * *  * *   *     ** **

BRCA1-5_UTR_Ex20dup-3_-RC  TACAGGC-GTGCGCCCCTACGCCCAGCTAATTTTTGTATTTTTAGTA--G
BRCA1-5_UTR_Ex20dup-5_     AGTAGGGGATGGGGTATTAGCTACAGAGGGCTTTGGGAATGGGACATGTG
                              ***   ** *    **    ***    *** * * *   *     *

BRCA1-5_UTR_Ex20dup-3_-RC  AGACGGGGTTTCGCC
BRCA1-5_UTR_Ex20dup-5_     GCACAAGAGGCCCCC
                             **  *    * **
```

Figure S8

BRCA1

```
CLUSTAL W (1.83) multiple sequence alignment

BRCA1-EX2dup-3_  -ACATCATTTTGAACAGTGTTTTGTATTTTTTGTGTCC-A-----TC-AG
BRCA1-EX2dup-5_  AACGTGCACTTCCCCGGTTC-AAGCGATTCTCCTGCCTTAGCCTCCCAAG
                  ** *    **   * **     *   ** *  ** *  *      * **

BRCA1-EX2dup-3_  TGAAAGCAAACTGCAAGCAGTTTTGAAATAAGCACATTGTGTTTGAGCCT
BRCA1-EX2dup-5_  TA-GCTGGGATTACAGGCGC-ATGCCACCATGCCCAGTTAGTTTTGGTAT
                 *        * * ** **    *   *  * ** ** *  ****  *  *

BRCA1-EX2dup-3_  TCCCAGTTTCTCCTTTCTGTTCATTTCTGCATATCCTTATGCATTCCCCC
BRCA1-EX2dup-5_  TTTTAGTAGAGACAG-CGTTTCACT-GTGTTGGCCAGGCTGGTCTCGAAA
                 *   ***     *   *  **** *  **     *    **   **

BRCA1-EX2dup-3_  TTCTAAGGGTCAGTGTTTGCCCGCTTTGTAATCA-TTGTGAAGACAGGAA
BRCA1-EX2dup-5_  TCCTGA-CCTCATGATCCGCCCGCCTCGGCCTCCCAAGTGCTG---GGAT
                 * ** *   ***   *  ****** * *   **    ***  *   ***

BRCA1-EX2dup-3_  AGGACCTGA
BRCA1-EX2dup-5_  TACAAGCAT
                    *
```

```
CLUSTAL W (1.83) multiple sequence alignment

BRCA1-EX2dup-3_-RC  -TCAGGTCCTTTCCTGTCTTCACAATGATTACAAAGCGGGCAAACACTGA
BRCA1-EX2dup-5_     AACGTGCACTTCCCCG-GTTCA-AGCGATTCTC---CTGCCTTAGCCT--
                      *  *  *** ** *  **** *  ****      * * *  *  **

BRCA1-EX2dup-3_-RC  CCCTTAGAAGGGGGAATGCATAAGGATATGCAGAAATGAACAGAAAGGA-
BRCA1-EX2dup-5_     -CCCAAGTAGCTGGGAT-TACAGGCGCATGCCACCATGCCCAGTTAGTTT
                     **  ** **  ** **  * * *   ****    ***  ***  **

BRCA1-EX2dup-3_-RC  -G--AAACTGGGAAGGCTCA--AACACAATGTGCTTATTTCAAAACTGCT
BRCA1-EX2dup-5_     TGGTATTTTTAGTAGAGACAGCGTTTCACTGTGTTG---GCCAGGCTGGT
                     *  *   *  * **   **      ** **** *     * *  *** *

BRCA1-EX2dup-3_-RC  T--GCAGT-TTGCTTTCACTGATGGACACA---AAAAATACAAAACACTG
BRCA1-EX2dup-5_     CTCGAAATCCTGACCTCA-TGATCCGCCCGCCTCGGCCTCCCAAGTGCTG
                       * * *  **   *** ****   * *         * * **   ***

BRCA1-EX2dup-3_-RC  TTCAAAATGATGT
BRCA1-EX2dup-5_     GGATTACAAGCAT
                         *      *
```

```
CLUSTAL W (1.83) multiple sequence alignment

BRCA1-EX3_11dup-3_  TCCTAAACCAGCCA--GCAAGTAGA-TCATC--ATGT-CACAAGGCTTAG
BRCA1-EX3_11dup-5_  AATACATCCAGGAAAAATAAGCTAACTTTGCATATGTGTATAGGAGTTGT
                         * ****  *    ***   * *   *  ****  * * *  **

BRCA1-EX3_11dup-3_  GTTGGGCTT----GC--TGTTCAGAGAATG-A----ATTAAGGATTAAGG
BRCA1-EX3_11dup-5_  GTTAGAAAAGGAAGAAGCCCTCAAAGATGGGAAGCCATT-TGCAAGAAAG
                    *** *        *      *** ***  * *    ***  * *  ** *

BRCA1-EX3_11dup-3_  AG-------AAAAAAAAGCAGAAAGGTTTTGCTCTGTTTTTCAGGTTCT-
BRCA1-EX3_11dup-5_  AGAAGGTCCAAGAGGAGGCAGAAGGGATTGGAAATAGAA-AAAGGATGTA
                    **       ** *  * ****** ** ** *   *       *** * *

BRCA1-EX3_11dup-3_  --ATTGAGTTGTTAACTTCT---AACA-------------------AGTT
BRCA1-EX3_11dup-5_  AGAAAGAGTTGATTATTACTCATAAACAGTAATGAAGGAAAAGGAGAGTA
                      *  ****** * * * **   **                     ***

BRCA1-EX3_11dup-3_  ATCTTA------TTTGCTTCATTGCATGAGGCCCATTGTAGTAAGAAGAG
BRCA1-EX3_11dup-5_  ATTCTACAGGAAGATGCTGAGGTGCTTTGAGCCCAGTGAAGTTGGAGG-T
                    **  **        ****    *** *   ***** ** ***  ** *

BRCA1-EX3_11dup-3_  GAATTT
BRCA1-EX3_11dup-5_  AAAGAC
                     **
```

```
CLUSTAL W (1.83) multiple sequence alignment

BRCA1-EX3_11dup-3_-RC  AAATTC--------------------CTCTTCTTACTACAATGGGCCTCA
BRCA1-EX3_11dup-5_     AA-TACATCCAGGAAAAATAAGCTAACTTTGCATATGTGTATAGGAGTTG
                       ** * *                    ** * * **     ** **  *

BRCA1-EX3_11dup-3_-RC  TGC-----AATGA---AG----CAAATAAGATA----ACTTGTTAGAAGT
BRCA1-EX3_11dup-5_     TGTTAGAAAAGGAAGAAGCCCTCAAAGATGGGAAGCCATTTGCAAGAAAG
                       **      ** **   **    **** * *  *    * ***  ****

BRCA1-EX3_11dup-3_-RC  TAACAACTCAATAGA-ACCTGAA--------AAACAGAGCAAAACC-TTT
BRCA1-EX3_11dup-5_     AGAAGGTCCAAGAGGAGGCAGAAGGGATTGGAAATAGA--AAAAGGATGT
                         *     *** **    * ***        *** ***  ****   * *

BRCA1-EX3_11dup-3_-RC  ------CTGCTTTTTTTTCTCCTTAATCCTTAATTCATTCTCTGAACAGC
BRCA1-EX3_11dup-5_     AAGAAAGAGTTGATTATTACTCATAAACAGTAATGAAGGAAAAGGAGAGT
                               * *  ** **   * *** *  ****  *      * * **

BRCA1-EX3_11dup-3_-RC  AAGCCCAACCTAAGCCTTGTGACATG--ATGATCTACTTG--CTGGCTGG
BRCA1-EX3_11dup-5_     AATTCTACAGGAAGA-TGCTGAGGTGCTTTGAGCCCAGTGAAGTTGGAGG
                       **  * *    ***  *  ***  **   *** *    **   * *  **

BRCA1-EX3_11dup-3_-RC  TTTAGGA
BRCA1-EX3_11dup-5_     TAAAGAC
                       *  **
```

```
CLUSTAL W (1.83) multiple sequence alignment

BRCA1-Ex6dup-3_-AluSp_II_  -GCCAGGCGCAGTGGCTCACGCCTGTAATCCCAGCACTTTGGGAGGCCGA
BRCA1-Ex6dup-5_-AluSc5     GGCTGGGTGCGGCGGCTAACACCTGCAATCCCAGCACCTTGGGAGGCTGA
                            **  ** ** * **** ** **** *********** ********* **

BRCA1-Ex6dup-3_-AluSp_II_  GGTGGGCGGATCACCTGAGGTCGGGAGTTCAAGACCAGCCTGACCAACAT
BRCA1-Ex6dup-5_-AluSc5     GGCCAGTGGATCA--TGAGGTCAGGAGATTGAGACCACCCTGGCCAACAT
                           **   * ******  ******* **** *  ****** **** *******

BRCA1-Ex6dup-3_-AluSp_II_  GGAGAAACCCCATCTCTACTAAAAAAAAAAAAAAAAAAATTAGCCGGG-G
BRCA1-Ex6dup-5_-AluSc5     GGTGAAACCCGGTCTCTACTAAAAATA------CAAAAATTAGCTGGGTG
                           ** *******  ************* *       ********** *** *

BRCA1-Ex6dup-3_-AluSp_II_  TGGTGGCTTATGCCTGTAATCCCAGCTACTCAGGAGGCTGAGGCAGGAGA
BRCA1-Ex6dup-5_-AluSc5     TGGTGGCACCTGCCTGTAATCCCAGCTACTCCAGAGGCTGAGGATGGAGA
                           *******   *********************  **********  *****

BRCA1-Ex6dup-3_-AluSp_II_  ATCGCTTGAACCCAGGAAGCAGAGGTTGCAGTGAGCCAAGATCGCACCAT
BRCA1-Ex6dup-5_-AluSc5     ATCACTTGAACCTGAGAGTCGGAGGTTGCAGTGAGCCGAGATTGCGCCAC
                           *** ********   **  * **************** **** ** ***

BRCA1-Ex6dup-3_-AluSp_II_  TGCACTCCAGCCTAGGCAACAAGAGTGAAACTCCATCTCAAAAAAA---A
BRCA1-Ex6dup-5_-AluSc5     TGCACTCCAGCCT-GGCGACA-GAGCGAGACTCCGTCTCAAAAAAAAAAA
                           ************* *** *** *** ** ***** ***********   *

BRCA1-Ex6dup-3_-AluSp_II_  AAAAAAA
BRCA1-Ex6dup-5_-AluSc5     AAAAAAA
                           *******
```

Figure S8

BRCA1

```
CLUSTAL W (1.83) multiple sequence alignment

BRCA1-EX11_12dup-3_-AluSx1_I_    ----------------------GCCAGGCGTGGT----------------
BRCA1-EX11_12dup-5_-AluSx-Y-Sx   CTACTAAAAATACAAAAATTAGGCCAGGTGTGGTGGCTCACGCCTGTAAT
                                                       ****** *****

BRCA1-EX11_12dup-3_-AluSx1_I_    --------------------------------------------------
BRCA1-EX11_12dup-5_-AluSx-Y-Sx   CCCAGCACTTTGGGAGGCCGAGGCGGGAGGATCATGTGGTCAGGAGATCC


BRCA1-EX11_12dup-3_-AluSx1_I_    --------------------------------------------------
BRCA1-EX11_12dup-5_-AluSx-Y-Sx   AGACCATCCTGGCTAACACGGTGAAACACCATTTCTACTAAAAATACAAA


BRCA1-EX11_12dup-3_-AluSx1_I_    ------------------GGCAGGTGCCTGTAATCCCAGCTACTCAGGAG
BRCA1-EX11_12dup-5_-AluSx-Y-Sx   AAATTAGCTGGGCATGGTGGCGGGCGCCTGTAATCCCAGCTACTCAGGAG
                                                   *** ** *************************

BRCA1-EX11_12dup-3_-AluSx1_I_    GCTGAGGCAGGAGAATTGCTTGAACCTGGGAGGCAGAGGTTGCAGTGAGC
BRCA1-EX11_12dup-5_-AluSx-Y-Sx   GCTGAAGCAGAAGAATGGCTTGAACCTGGGAGGCGGAGCTTGCAGTGAAC
                                 ***** **** ***** ***************** *** ********* *

BRCA1-EX11_12dup-3_-AluSx1_I_    TGGGACCGTGCCATTGCACTCCAGCCTGGGCGACAGAGCAAGACTCCGTC
BRCA1-EX11_12dup-5_-AluSx-Y-Sx   CAAGATTGCGCCACTGCACTCCAGCCTGGGCAACAAAGTGAGACTCCGTC
                                    **  * **** ***************** *** **  **********

BRCA1-EX11_12dup-3_-AluSx1_I_    TCAAAACAAAAAAAAA----------------A
BRCA1-EX11_12dup-5_-AluSx-Y-Sx   TCAAAAAAAAAAAAAAAAATTAGTCGGGCATGG
                                 ****** *********
```

```
CLUSTAL W (1.83) multiple sequence alignment

BRCA1-EX11_14dup-3_-AluSp        ---------------------GGCCGGGCATGGTGGCTCAAGCCTGTAAT
BRCA1-EX11_14dup-5_-AluSx-Y-Sx   CTACTAAAAATACAAAAATTAGGCCAGGTGTGGTGGCTCACGCCTGTAAT
                                                      **** **  ********** *********

BRCA1-EX11_14dup-3_-AluSp        CCCAGCACTTTGGGAGGCTGAGGTGGTCAGATCACCTAAGGTCAGGAGAT
BRCA1-EX11_14dup-5_-AluSx-Y-Sx   CCCAGCACTTTGGGAGGCCGAGGCGGGAGGATCATG--TGGTCAGGAGAT
                                 ****************** **** **   *****    ***********

BRCA1-EX11_14dup-3_-AluSp        CAAGACCAGCCTGACCAACATGAAGAAACCCCATCTTTACTAAAAATACA
BRCA1-EX11_14dup-5_-AluSx-Y-Sx   CCAGACCATCCTGGCTAACACGGTGAAACACCATTTCTACTAAAAATACA
                                 * ****** **** * **** *  ***** **** * *************

BRCA1-EX11_14dup-3_-AluSp        C--AATTAGCCGGGCGTGGTGGCGCATGCCTATAATCCCAGCTACTCAGG
BRCA1-EX11_14dup-5_-AluSx-Y-Sx   AAAAATTAGCTGGGCATGGTGGCGGGCGCCTGTAATCCCAGCTACTCAGG
                                    ******* **** ********   **** ******************

BRCA1-EX11_14dup-3_-AluSp        AGGCTGAGGCAGGAGAATTGCTTGAACCCGGGAGGCGGAGGTTGCGGTGA
BRCA1-EX11_14dup-5_-AluSx-Y-Sx   AGGCTGAAGCAGAAGAATGGCTTGAACCTGGGAGGCGGAGCTTGCAGTGA
                                 ******* **** ***** ********* *********** **** ****

BRCA1-EX11_14dup-3_-AluSp        GCCGAGATTGCACCATTGCACTCCAGCCTGGGCAACAAGAGCGAAACTCT
BRCA1-EX11_14dup-5_-AluSx-Y-Sx   ACCAAGATTGCGCCACTGCACTCCAGCCTGGGCAACAA-AGTGAGACTCC
                                  ** ******* *** ********************** ** ** ****

BRCA1-EX11_14dup-3_-AluSp        GTCTCAATAATAAGAAGAAG-------------AA
BRCA1-EX11_14dup-5_-AluSx-Y-Sx   GTCTCAAAAAAAAAAAAAAAATTAGTCGGGCATGG
                                 ******* ** ** ** **
```

```
CLUSTAL W (1.83) multiple sequence alignment

BRCA1-EX11dup-3_-FLAMC-AluSx1-FLAMC  GGCTGGGTATGGTGGCTCATGCCTATAATCCCAGCACTTTGTGAGGCCAA
BRCA1-EX11dup-5_-AluSx-Y-Sx          -GCCAGGCACAGTGACTCACGTCTGTAATCCCAGCACTTTGGGAGGCCGA
                                      **  ** *  *** **** * ** **************** ****** *

BRCA1-EX11dup-3_-FLAMC-AluSx1-FLAMC  GGTTGGCGGATCACCTGAGGTCAGGAGTTTAAGACCAGCCTGGCCAACAT
BRCA1-EX11dup-5_-AluSx-Y-Sx          GGCGGGCAGATCACCTGAGGTCAGGAGTTCAAGACCAGCCTGGCCAACAT
                                     **  *** ********************* ********************

BRCA1-EX11dup-3_-FLAMC-AluSx1-FLAMC  GGTAAAACCCCATCTCTACAAAAAATACAAAAATTAGCCAAGGGGTGGTG
BRCA1-EX11dup-5_-AluSx-Y-Sx          GGTGAAACCCC---------------------------------------
                                     *** *******

BRCA1-EX11dup-3_-FLAMC-AluSx1-FLAMC  GTGGGCACCTATAATCCCATCTTCTTGGGAGGCTAAGGCAGGAGAATCGC
BRCA1-EX11dup-5_-AluSx-Y-Sx          --------------------------------------------------


BRCA1-EX11dup-3_-FLAMC-AluSx1-FLAMC  TTGAACCTGGAGGCAGAGGTTGCAGTGAGCCGAGATCATGCCACTGTATT
BRCA1-EX11dup-5_-AluSx-Y-Sx          --------------------------------------------------


BRCA1-EX11dup-3_-FLAMC-AluSx1-FLAMC  CCAGCCTGGGTAACAGAGCAAGACTCTGTCTCAAAAAAAAAAAAA
BRCA1-EX11dup-5_-AluSx-Y-Sx          ---------------------------GTC--------------T
                                                                ***
```

Figure S8

BRCA1

```
CLUSTAL W (1.83) multiple sequence alignment

BRCA1-EX12_14dup-3_-AluSp               GGCCGGGCATGGTGGCTCAAGCCTGTAATCCCAGCACTTTGGGAGGCTGA
BRCA1-EX12_14dup-5_-FLAMC-AluSx1-FLAMC  GGCTGGGTATGGTGGCTCATGCCTATAATCCCAGCACTTTGTGAGGCCAA
                                        *** *** *********** **** **************** *****  *

BRCA1-EX12_14dup-3_-AluSp               GGTGGTCAGATCACCTAAGGTCAGGAGATCAAGACCAGCCTGACCAACAT
BRCA1-EX12_14dup-5_-FLAMC-AluSx1-FLAMC  GGTTGGCGGATCACCTGAGGTCAGGAGTTTAAGACCAGCCTGGCCAACAT
                                        *** * * ******** ********** * ************ *******

BRCA1-EX12_14dup-3_-AluSp               GAAGAAACCCCATCTTTACTAAAAATACAC-AATTAGCCG-GGCGTGGTG
BRCA1-EX12_14dup-5_-FLAMC-AluSx1-FLAMC  GGTAAAACCCCATCTCTACAAAAAATACAAAAATTAGCCAAGGGGTGGTG
                                        *   *********** *** *********  ********  ** ******

BRCA1-EX12_14dup-3_-AluSp               GCGCATGCCTATAATCCCAGCTACTCAGGAGGCTGAGGCAGGAGAATTGC
BRCA1-EX12_14dup-5_-FLAMC-AluSx1-FLAMC  GTGGGCACCTATAATCCCATCTTCTTGGGAGGCTAAGGCAGGAGAATCGC
                                        * *    ************ ** **  ******* ************ **

BRCA1-EX12_14dup-3_-AluSp               TTGAACCCGGGAGGCGGAGGTTGCGGTGAGCCGAGATTGCACCATTGCAC
BRCA1-EX12_14dup-5_-FLAMC-AluSx1-FLAMC  TTGAACCT-GGAGGCAGAGGTTGCAGTGAGCCGAGATCATGCCACTGTAT
                                        *******  ****** ******** ************    *** ** *

BRCA1-EX12_14dup-3_-AluSp               TCCAGCCTGGGCAACAAGAGCGAAACTCTGTCTCAATAATAAGAAGAAGA
BRCA1-EX12_14dup-5_-FLAMC-AluSx1-FLAMC  TCCAGCCTGGGTAACA-GAGCAAGACTCTGTCTCAAAAAA---AAAAAAA
                                        *********** **** **** * ************ **    ** ** *

BRCA1-EX12_14dup-3_-AluSp               A
BRCA1-EX12_14dup-5_-FLAMC-AluSx1-FLAMC  A
                                        *
```

```
CLUSTAL W (1.83) multiple sequence alignment

BRCA1-Ex16_17dup-3_-AluSz_III_  GGTCGTGTGTGGTGGCTCGTGCCTATAATCCCAGCACTTTGGGAAGCCAA
BRCA1-Ex16_17dup-5_-AluSg       -GCTGGACGCGGTGGCTCGCATCAGTAATCCCAGCACTTTGGGAGCCTGA
                                 *  *   * *********   *  *******************  *  *

BRCA1-Ex16_17dup-3_-AluSz_III_  GGCAGGCAAATCACTTGAGGTCAGGAGTTCGAGACCAGCCTGGCCAACAT
BRCA1-Ex16_17dup-5_-AluSg       GGCGGGCAGATCAC--AAGGTCAGGAGTTCGAGACCAGCCTGGCCAATAT
                                *** **** *****   ****************************** **

BRCA1-Ex16_17dup-3_-AluSz_III_  GGTGAAACCCCATCTCTACTAAAAATACAAAAAACTAGCATGGTGTGGTG
BRCA1-Ex16_17dup-5_-AluSg       GGTAAAACCCAGTCTCTACTAAAAATACATA-AATTGGCTGGGCGTGGTG
                                *** ******  ***************** * ** * **  ** ******

BRCA1-Ex16_17dup-3_-AluSz_III_  GTGTGTGCCTGTAATCCCAGTGCCTGTAATCCCAGCTACTCCAGAGGCTG
BRCA1-Ex16_17dup-5_-AluSg       GTGTGCAC---------------CTGTAATCCCAGCTACTTGGGAGGCTG
                                *****  *               *****************   *******

BRCA1-Ex16_17dup-3_-AluSz_III_  AGGCAGGAGAATCACTTGAAC-CGAAAGGTAGGAGTTACAGTGAGCCAAG
BRCA1-Ex16_17dup-5_-AluSg       ACACAGAAGAATTGCTTGAACTCTGGAGGCTGAGGTTGCAGTCAGCTGAG
                                *  *** *****  ******* *   ***  *  *** **** ***  **

BRCA1-Ex16_17dup-3_-AluSz_III_  ATCGCATCACTGCACTCCAGCCTGAACGACAGAGCAAGACTCCTGTCTCA
BRCA1-Ex16_17dup-5_-AluSg       ATCCTGCCACTGCACTCCAGCCTG-GCGACAGAGTGAGACT-CTGTCTCA
                                ***    *****************  ********  ***** ********

BRCA1-Ex16_17dup-3_-AluSz_III_  AGAAATAATAATGAT---AAAA
BRCA1-Ex16_17dup-5_-AluSg       ACAACAACAAAAGAAAGAACAA
                                * **  *  ** **    * **
```

```
CLUSTAL W (1.83) multiple sequence alignment

BRCA1-Ex16_18dup-3_-AluSq2  AGGCTGGTCGCGGTGGCTCACACCTGTGATCCCAGCACTTTGGGAGCCCA
BRCA1-Ex16_18dup-5_-AluSg   --GCTGGACGCGGTGGCTCGCATCAGTAATCCCAGCACTTTGGGAGCCTG
                              ***** *********** ** * ** ********************

BRCA1-Ex16_18dup-3_-AluSq2  AGGCGGGTGGATCACCTGAGGTCAGGAGTTCGAGACCAGCCTGGCCAACA
BRCA1-Ex16_18dup-5_-AluSg   AGGCGGGCAGATCAC--AAGGTCAGGAGTTCGAGACCAGCCTGGCCAATA
                            *******  ******   ****************************** *

BRCA1-Ex16_18dup-3_-AluSq2  TGGTGAAACCCCATCTCTACTAAAAATACAAAAGTTAACTGGGCGTGGTG
BRCA1-Ex16_18dup-5_-AluSg   TGGTAAAACCCAGTCTCTACTAAAAATACATAAATTGGCTGGGCGTGGTG
                            **** ******  ***************** ** **  ************

BRCA1-Ex16_18dup-3_-AluSq2  GTAGGCACCTGTAATCCTAGCTCAGGATTACTCGGGAGGCTGAGGCTAGA
BRCA1-Ex16_18dup-5_-AluSg   GTGTGCACCTGTAATCCCAGC-------TACTTGGGAGGCTGACACAGAA
                            **  ************* ***       **** **********  *   *

BRCA1-Ex16_18dup-3_-AluSq2  GAATCGGTTGAACCCAGGAGGCGGAGGTTGCAGTGAGCCAAGATTGCGCC
BRCA1-Ex16_18dup-5_-AluSg   GAATTGCTTGAACTCTGGAGGCTGAGGTTGCAGTCAGCTGAGATCCTGCC
                            **** * ****** * ****** *********** ***  ****   ***

BRCA1-Ex16_18dup-3_-AluSq2  ACTGCACTCTAGCCTGGGGACAAGAGGGAGACTTCATCTCAAAAA-----
BRCA1-Ex16_18dup-5_-AluSg   ACTGCACTCCAGCCTGGCGACA-GAGTGAGACTCTGTCTCAACAACAACA
                            ********* ******* **** *** ******   ****** **

BRCA1-Ex16_18dup-3_-AluSq2  --AAAAAAAAAAA
BRCA1-Ex16_18dup-5_-AluSg   AAAGAAAGAACAA
                              * *** ** **
```

Figure S8

BRCA1

```
CLUSTAL W (1.83) multiple sequence alignment

BRCA1-Ex19_20dup-3_-AluSz  GGCTGGGCATGGTGGCTCATGTCTGTAATCCCAGCACTTTGGGAGGCCGA
BRCA1-Ex19_20dup-5_-AluSg  --CCGGGCGCAGTGGCTTATGCCTGTAATCCCAGCACTTTGGAAGGCCAA
                             * ****   ****** *** ******************** ***** *

BRCA1-Ex19_20dup-3_-AluSz  GGCAGGTGGATCACTT-GTCAGGAGTTTGAGACCAGCCTGGCCAACATGG
BRCA1-Ex19_20dup-5_-AluSg  GACAGGCAGATCACGAGGTCAGGAGTTCGAGACCAGCCTGACCAACATGG
                           * ****  ******   ********** ************ *********

BRCA1-Ex19_20dup-3_-AluSz  TGAAACCCCATCTGTACTAAAAAAAAAAAAACAAAAAGTAGCCAGGCCTG
BRCA1-Ex19_20dup-5_-AluSg  TGAAACCCTGTCTCTACTAAAA------ATACAAAAATTAGCCGGGCGTG
                           ********  *** ********      * ******* ***** *** **

BRCA1-Ex19_20dup-3_-AluSz  GTGGAGCATGCCTGTAATGCCAGCTATTTGGGAAGTTGAGGTGTGAGCAT
BRCA1-Ex19_20dup-5_-AluSg  GTGGTGCGCGCCTGTAATCCCAGCTACTCAGGAGGCTAAGGCAGGAGAAT
                           **** **  ********* ******* *  *** * * ***   *** **

BRCA1-Ex19_20dup-3_-AluSz  CGCTTGAACGTGGGAGGCAGAGGTTGCAGTGAGCCAAGATTGCACCACTG
BRCA1-Ex19_20dup-5_-AluSg  CGCTTGAACCCTGGAGGCAGAGGTTGCAGTGAGCCCAGATCGCACCACTA
                           *********   *********************** **** ********

BRCA1-Ex19_20dup-3_-AluSz  CACTCCAGACTGGGTGACAGAGTGAGACTTTGTCTAAAAAAAAAAAAAAA
BRCA1-Ex19_20dup-5_-AluSg  CGCTCCAGCCTAGGTGACAGAGAGAGACTCCGTCTCAAAAAAAAAAAA--
                           * ****** ** ********** ******  **** ************

BRCA1-Ex19_20dup-3_-AluSz  GAGAGAGAGAGAAAA
BRCA1-Ex19_20dup-5_-AluSg  -----------AAAA
                                      ****
```

```
CLUSTAL W (1.83) multiple sequence alignment

BRCA1-Ex21_3_UTRdup-3_-MIR1_Amn  --CTTTGCA----GGTTCA------------AAGCACT------AGCCCA
BRCA1-Ex21_3_UTRdup-5_-AluSz     GGCTGGGCATGGTGGCTCATGTCTGTAATCCCAGCACTTTGGGAGGCCGA
                                   **  ***    ** ***             ******       *** *

BRCA1-Ex21_3_UTRdup-3_-MIR1_Amn  GCCAT---TATCTCT-----------------------------------
BRCA1-Ex21_3_UTRdup-5_-AluSz     GGCAGGTGGATCACTTGTCAGGAGTTTGAGACCAGCCTGGCCAACATGGT
                                 * **     *** **

BRCA1-Ex21_3_UTRdup-3_-MIR1_Amn  -----CCCA-CTTCACTAGGATAAA-----------ATTGCTA-------
BRCA1-Ex21_3_UTRdup-5_-AluSz     GAAACCCCATCTGTACTAAAAAAAAAAAAACAAAAAGTAGCCAGGCCTGG
                                      **** **  ****  * ***            * ** *

BRCA1-Ex21_3_UTRdup-3_-MIR1_Amn  ---------------ACCCCA-CTTTAT---------AGGTG--------
BRCA1-Ex21_3_UTRdup-5_-AluSz     TGGAGCATGCCTGTAATGCCAGCTATTTGGGAAGTTGAGGTGTGAGCATC
                                                *  *** ** * *         *****

BRCA1-Ex21_3_UTRdup-3_-MIR1_Amn  -CTAAAACA-----GGTCCAGGGCCTTGT---CAAAGGTC-----ACT-C
BRCA1-Ex21_3_UTRdup-5_-AluSz     GCTTGAACGTGGGAGGCAGAGGTTGCAGTGAGCCAAGATTGCACCACTGC
                                  **  ***      **   ***     **   * *** *      *** *

BRCA1-Ex21_3_UTRdup-3_-MIR1_Amn  AGT---GAGCTGGTGGCAGA------CCTGG-----AAATA------ACT
BRCA1-Ex21_3_UTRdup-5_-AluSz     ACTCCAGACTGGGTGACAGAGTGAGACTTTGTCTAAAAAAAAAAAAAAAG
                                 * *   **   **** ****      * * *     *** *      *

BRCA1-Ex21_3_UTRdup-3_-MIR1_Amn  AGCCTAGGAGTCTC
BRCA1-Ex21_3_UTRdup-5_-AluSz     AGAGAGAGAGAAAA
                                 **     ***
```

```
CLUSTAL W (1.83) multiple sequence alignment

BRCA1-Ex21_3_UTRdup-3_-MIR1_Amn-RC  G-----------AGACTC-----------C------------------TA
BRCA1-Ex21_3_UTRdup-5_-AluSz        GGCTGGGCATGGTGGCTCATGTCTGTAATCCCAGCACTTTGGGAGGCCGA
                                    *            * ***           *                   *

BRCA1-Ex21_3_UTRdup-3_-MIR1_Amn-RC  GGCTAG----TTATT-TCCAG--GTCTGCCACCAGCTC-----ACTGAGT
BRCA1-Ex21_3_UTRdup-5_-AluSz        GGCAGGTGGATCACTTGTCAGGAGTTTGAGACCAGCCTGGCCAACATGGT
                                    ***  *    * * *   ***  ** **  ******       **   **

BRCA1-Ex21_3_UTRdup-3_-MIR1_Amn-RC  GA--CC---TTTG----------------ACAA----------GGCCCT-
BRCA1-Ex21_3_UTRdup-5_-AluSz        GAAACCCCATCTGTACTAAAAAAAAAAAAACAAAAAGTAGCCAGG-CCTG
                                    **  **   * **                ****          ** ***

BRCA1-Ex21_3_UTRdup-3_-MIR1_Amn-RC  --GGA-----CCTGTTTTAGCACCTAT----AAAGT---GGGGTTAGCAA
BRCA1-Ex21_3_UTRdup-5_-AluSz        GTGGAGCATGCCTGTAATGCCAGCTATTTGGGAAGTTGAGGTGTGAGCAT
                                      ***     ***** *  ** ****     ****   ** ** ****

BRCA1-Ex21_3_UTRdup-3_-MIR1_Amn-RC  --TTTTATCCTAG-----TGAAGTGGGAG-------AGATA------AT-
BRCA1-Ex21_3_UTRdup-5_-AluSz        CGCTTGAACGTGGGAGGCAGAGGTTGCAGTGAGCCAAGATTGCACCACTG
                                       ** * * * *      ** ** * **       ****        *

BRCA1-Ex21_3_UTRdup-3_-MIR1_Amn-RC  -------GGCTGGGCT----AGTG---CTTTG----AA-----------C
BRCA1-Ex21_3_UTRdup-5_-AluSz        CACTCCAGACTGGGTGACAGAGTGAGACTTTGTCTAAAAAAAAAAAAAAA
                                           * *****      ****   *****    **

BRCA1-Ex21_3_UTRdup-3_-MIR1_Amn-RC  -------CTGCAAAG
BRCA1-Ex21_3_UTRdup-5_-AluSz        GAGAGAGAGAGAAAA
                                               ***
```

Figure S8 *BRCA2*

```
CLUSTAL W (1.83) multiple sequence alignment

BRCA2-Ex3dup-3_-AluY  TTTTTTTTTTGTTTTTTTTTTTTTTTGAGATGGAGTCTC----TGTCACCC
BRCA2-Ex3dup-5_-AluY  TTTAATTTTT-TTTTTTTTTTTTTTGGAGATGAAATCTCGCTTTGTCGCCC
                      ***  ***** ************* ****** * ****    **** ***

BRCA2-Ex3dup-3_-AluY  AGGCTGTAGTGCAGTGGTGCGATCTCAGCTCACTGCAAGCTCCGCCTCCC
BRCA2-Ex3dup-5_-AluY  AGGCTGGAGTGCAGTGGCGTGATCTTGGCTCACTGCAAGCTCCGCCTCCC
                      ****** ********** * *****  ***********************

BRCA2-Ex3dup-3_-AluY  GGGTTCACGCCATTCTCCTGCCTCAGCCTCCCAAGTAGCTGGGACTACAG
BRCA2-Ex3dup-5_-AluY  AGGTTCACGCCATTCTCCTGCCTTAGCCTCCTGAGTAGCTGGGACTACAG
                       ********************** *******  *****************

BRCA2-Ex3dup-3_-AluY  GCGCCCGCCACCATGCCCGGCTAATTT-TTTGTATTTTTAGTAGAGACGG
BRCA2-Ex3dup-5_-AluY  GCACCCGCCACCACACCTGGCTGATTTTTTTGTATTTTTAGTAGAGACGG
                      ** **********  ** **** **** **********************

BRCA2-Ex3dup-3_-AluY  GGTTTCACCGTGTTAGCTAGGATGGTCTCGATTTCCTGACCTCGTGATCC
BRCA2-Ex3dup-5_-AluY  GGTTTCACTGTGTTAGCCAGGATGGTCTTGATCTCCTGACCTCGTGATCC
                      ******** ******** ********** *** *****************

BRCA2-Ex3dup-3_-AluY  GCCCGCCTCAGCCTCCCAGACTGCTGGGATTACAGGCGTGAACCACTGTG
BRCA2-Ex3dup-5_-AluY  GCTCGCCTCGGCCTCCCAAAGTGCTGGGATTACAGGCGTGAGCCATCATG
                      ** ****** ******** * ******************** ***    **

BRCA2-Ex3dup-3_-AluY  CCCGGCC
BRCA2-Ex3dup-5_-AluY  CCCGGCC
                      *******
```

```
CLUSTAL W (1.83) multiple sequence alignment

BRCA2-Ex4_10dup_part._-3_       GCCCAAA--CACTAC----CTTTTTAAC--------TTAGTGAAA-AATA
BRCA2-Ex4_10dup_part._-5_-AluY  TTTTTTTTTTGTTTTTTTTTTTTTTTGAGATGGAGTCTCTGTCACCCAGGC
                                           *       ***** *          *  ** *    *

BRCA2-Ex4_10dup_part._-3_       TTTAGTGAA----TGTGATTG-ATGGTACTTTAATTT-TG----------
BRCA2-Ex4_10dup_part._-5_-AluY  TGTAGTGCAGTGGTGCGATCTCAGCTCACTGCAAGCTCCGCCTCCCGGGT
                                * ***** *    ** ***   *    ***  **  *  *

BRCA2-Ex4_10dup_part._-3_       -----TCACTTT-GTGTT-------TTTATGT--TTAGGTTTATT-----
BRCA2-Ex4_10dup_part._-5_-AluY  TCACGCCATTCTCCTGCCTCAGCCTCCCAAGTAGCTGGGACTACAGGCGC
                                      ** * *  **            * **   * **  **

BRCA2-Ex4_10dup_part._-3_       ----------GC------ATTCTTCTGT-------GAAAAGAAGCTG-TT
BRCA2-Ex4_10dup_part._-5_-AluY  CCGCCACCATGCCCGGCTAATTTTTGTATTTTTAGTAGAGACGGGGTTT
                                          **      * * ** ***       * * *** *  * **

BRCA2-Ex4_10dup_part._-3_       CAC----------AGAATGATTCTGAAGAACCAACTTTGTC---------
BRCA2-Ex4_10dup_part._-5_-AluY  CACCGTGTTAGCTAGGATGGTCTCGATTTCCTGACCTCGTGATCCGCCCG
                                ***          ** *** *   **    *  ** * **

BRCA2-Ex4_10dup_part._-3_       -CTTAACTAGC---TCTTTTGGGA---CAATTCTGAGGAAATGTTC-TAG
BRCA2-Ex4_10dup_part._-5_-AluY  CCTCAGCCTCCCAGACTGCTGGGATTACAGGCGTGAACCACTGTGCCCGG
                                 ** * *    *    **  *****   **     ***   * *** *   *

BRCA2-Ex4_10dup_part._-3_       AA
BRCA2-Ex4_10dup_part._-5_-AluY  CC
```

```
CLUSTAL W (1.83) multiple sequence alignment

BRCA2-Ex4_10dup_part._-3_-RC    TT-----CTAGAACATT----TCCTCAGAA-------TTGTCCCAAAAG-
BRCA2-Ex4_10dup_part._-5_-AluY  TTTTTTTTTTGTTTTTTTTTTTTTTTGAGATGGAGTCTCTGTCACCCAGGC
                                **       * *    **    *  * ***         **** *  * *

BRCA2-Ex4_10dup_part._-3_-RC    -------------AGCTAGTTAAGGACA----AAGTTGGTTCTT-CAGAA
BRCA2-Ex4_10dup_part._-5_-AluY  TGTAGTGCAGTGGTGCGATCTCAGCTCACTGCAAGCTCCGCCTCCCGGGT
                                              ** *  * **  **    *** *    **  * *

BRCA2-Ex4_10dup_part._-3_-RC    T-----CATTCTG-TG-AACAGCTTCTT-----------TTCACAGAAGA
BRCA2-Ex4_10dup_part._-5_-AluY  TCACGCCATTCTCCTGCCTCAGCCTCCCAAGTAGCTGGGACTACAGGCGC
                                *     ******  **   **** **                ****  *

BRCA2-Ex4_10dup_part._-3_-RC    ATGCAAT-AAAC----CTAAACAT----------A-AAAACACAAAGTGA
BRCA2-Ex4_10dup_part._-5_-AluY  CCGCCACCATGCCCGGCTAATTTTTGTATTTTTAGTAGAGACGGGGTTT
                                  ** *  *  *    ****   *          *  * * **   **

BRCA2-Ex4_10dup_part._-3_-RC    CAA--AATTA----AAGTACCATCAATCACA-----------TTCA----
BRCA2-Ex4_10dup_part._-5_-AluY  CACCGTGTTAGCTAGGATGGTCTCGATTTCCTGACCTCGTGATCCGCCCG
                                **    ***       *    ** **  *             * *

BRCA2-Ex4_10dup_part._-3_-RC    -CTAAATATTTTTCAC---TAAG-TTAAAAAGGT-------AGTGTTTGG
BRCA2-Ex4_10dup_part._-5_-AluY  CCTCAGCCTCCCAGACTGCTGGGATTACAGGCGTGAACCACTGTGCCCGG
                                 ** *   *     **   *  * *** *   **        ***   **

BRCA2-Ex4_10dup_part._-3_-RC    GC
BRCA2-Ex4_10dup_part._-5_-AluY  CC
                                 *
```

```
CLUSTAL W (1.83) multiple sequence alignment

BRCA2-Ex11_12dup-3_-AluSz  GGCCAGGCACAGTGGCTTACACCTGTAATCCAGCACATTGGGAGGCTGAG
BRCA2-Ex11_12dup-5_        --------ACACCTCCTAAAGCCTTGCAT---GGACATTT----------
                                   ***    ** *  ***    **    * *****

BRCA2-Ex11_12dup-3_-AluSz  GTGGGTAGATCACTTGAGGCCAGGAGTTCGAGACTAGCCTGGCCAACATG
BRCA2-Ex11_12dup-5_        ---------TTACTTA----------TT--AAATTAT-----ACAAA---
                                    * ****           **  * * **       ***

BRCA2-Ex11_12dup-3_-AluSz  GTAAAATCCCATCTCTACTGAAAATACAAAAATTAGCCAGGCGTGGTGGC
BRCA2-Ex11_12dup-5_        -TTTATTCC---CTGTAATAAAGCATCAAAA---AGCAAAGTAC-CTGTT
                            *  * ***   ** ** * **    *****   *** * *     **

BRCA2-Ex11_12dup-3_-AluSz  ACACGCCTGTAATCCCAGCTACTCCAGAGGCTGAGGCACAAGAACCGCTT
BRCA2-Ex11_12dup-5_        ATA----TATTATCTCAGC-----------ATGA--CATG-GAAATGCCT
                           * *    * * *** ****           ***  **   ***  ** *

BRCA2-Ex11_12dup-3_-AluSz  GAACCTTTGAGGTGGAGGTTGTGGTAAAATTGCACC-ACTGCACTTCAGC
BRCA2-Ex11_12dup-5_        --ACCT-TGAA-------TTATGGTTTAATCTTACCCTCTTAGCCTCTGT
                             **** ***       ** ****  ***   ***  **   * ** *

BRCA2-Ex11_12dup-3_-AluSz  CTGGGTGACAGAGTGAGACTCTGTCTCAAAAAAAAAAAAAAAAAAAAAAA
BRCA2-Ex11_12dup-5_        A-GAATTTTAAATAAGAAT-TGTTTCTATT----------ACTAGTACT
                             *  *     * * *** * *** ** *            *  *  *
```

```
CLUSTAL W (1.83) multiple sequence alignment

BRCA2-Ex11_12dup-3_-AluSz-RC  TTTTTTTTTTTTTTTTTTTTTTTTTGAGACAGAGTCTCACTCTGTCACCCAG
BRCA2-Ex11_12dup-5_           ACACCTCCTAA--AGCCTTGCATG-GAC--ATTTTTACT--------TA-
                                   *  *        **   ** ***  * * * ***         *

BRCA2-Ex11_12dup-3_-AluSz-RC  GCTGAAGTGCAGTGGTGCAATTTTACCACAACCTCCACCTCAAAGGTTCA
BRCA2-Ex11_12dup-5_           -TTAAA------TTATACAAATTTATT---CCCTGTA--ATAAAGCATCA
                                * **      *  * *** ****      ***  *     ****  ***

BRCA2-Ex11_12dup-3_-AluSz-RC  AGCGGTTCTTGTGCCTCAGCCTCTGGAGTAGCTGGGATTACAGGCGTGTG
BRCA2-Ex11_12dup-5_           AAAAGC-----------------AAAGTACCTGT---TATATA-TTATC
                              *   *                    **** ***    ** *    * *

BRCA2-Ex11_12dup-3_-AluSz-RC  CCACCACGCCTGGCTAATTTTTGTATTTTCAGTAGAGATGGGATTTTACC
BRCA2-Ex11_12dup-5_           TCAGCATGACATGGAAATGC--CTACCTTGAATTATGGTTTAATCTTACC
                               ** ** * *  *  ***     **  ** * *   * *   ** *****

BRCA2-Ex11_12dup-3_-AluSz-RC  ATGTTGGCCAGGCTAGTCTCGAACTCCTGGCCTCAAGTGATCTACCCACC
BRCA2-Ex11_12dup-5_           CTCTTAGCCTC-----TGTAGAATTT------------------------
                               * ** ***      * * *** *

BRCA2-Ex11_12dup-3_-AluSz-RC  TCAGCCTCCCAATGTGCTGGATTACAGGTGTAAGCCACTGTGCCTGGCC
BRCA2-Ex11_12dup-5_           ----------------TTAAATAAGAATTGTT-TCTATTACTAG-TACT
                                              *  ** * *  ***   * * *        *
```

Figure S8

BRCA2

```
CLUSTAL W (1.83) multiple sequence alignment

BRCA2-EX11_17dup-3_-AluSg  GTTTTTTTTTGGTTTTTTTTGTTTTTTTTTTAGATGGAGTCTTGCTCTG
BRCA2-EX11_17dup-5_        --T-------AAGTG---------TATAACTTAGTCAGCT------TCTG
                             *         **          * *   ****   *        ****

BRCA2-EX11_17dup-3_-AluSg  TTGCCCAGGCTAGAGTGCAGTGGTGCGATCTCGGCTCACTGCAACCTCCA
BRCA2-EX11_17dup-5_        GTTACTG--GAACAGTACAG-------GTCACTGTTAA--ACAAT-TAAA
                            *  *      * *** ***        **  * * * *   ***  *  *

BRCA2-EX11_17dup-3_-AluSg  CCTCCCAGGTTCGAGGAATTCTCCTGCCTCAGCCTCCCGAGTAGCTGGGA
BRCA2-EX11_17dup-5_        CC----ACTTTTA--TAATAATC-TAA--CA-CCTCC-------------
                           **    *  **     ***  ** *    ** *****

BRCA2-EX11_17dup-3_-AluSg  CTACAGGCGCCTGCCACCACCCCTGGCTAATTTTTTATTTTTAGTAGAGA
BRCA2-EX11_17dup-5_        -TAAAG---CCTTGC-------ATGGACA-TT-TTTACT--TATTAAA--
                            ** **   ***  *        ***  * ** **** *  ** ** *

BRCA2-EX11_17dup-3_-AluSg  CGGGGTTTCACCATCTTGGCCAGGCTGGTCTCCAACTCCTGACCTTGTGA
BRCA2-EX11_17dup-5_        -----TTATACAAATTTATT---------------------CCCTGTAA
                                ** ** *  **                         ** *** *

BRCA2-EX11_17dup-3_-AluSg  TCCACCCGCCTCGGCCTCCCAAAGTGTTGGGATTACAGGCGTGAGCCACA
BRCA2-EX11_17dup-5_        TAAA---GCATCAAA-AAGCAAAGTACCTGTTATATAT---TATCTCAGC
                           *  *   ** **       ******    *   ** *    *    **

BRCA2-EX11_17dup-3_-AluSg  GCGCCCGGCC
BRCA2-EX11_17dup-5_        ATGACATGGA
                             * *  *
```

```
CLUSTAL W (1.83) multiple sequence alignment

BRCA2-EX11_17dup-3_-AluSg-RC  GGCCGGGCGCTGTGGCTCACGCCTGTAATCCCAACACTTTGGGAGGCCGA
BRCA2-EX11_17dup-5_           ---TAAG-----TG---------TATA--------ACTTAGTCAGCTTCT
                                    *     **         * **        **** *  **

BRCA2-EX11_17dup-3_-AluSg-RC  GGCGGGTGGATCACAAGGTCAGGAGTTGGAGACCAGCCTGGCCAAGATGG
BRCA2-EX11_17dup-5_           GGTTACTGGAAC--------AGTA----CAGGTCA--CTGTTA--AACAA
                              **    **** *        ** *     **  **  ***      *

BRCA2-EX11_17dup-3_-AluSg-RC  TGAAACCCCGTCTCTACTAAAAATAAAAAATTAGCCAGGGGTGGTGGCAG
BRCA2-EX11_17dup-5_           TTAAACCACTTTTA---TAATAAT------CTAA-------------CA-
                              * ***** * * *    *** ***       **              **

BRCA2-EX11_17dup-3_-AluSg-RC  GCGCCTGTAGTCCCAGCTACTCGGGAGGCTGAGGCA-GGAGAATTCCTCG
BRCA2-EX11_17dup-5_           CCTCCTAAAGCC-----------------T--TGCATGGACA-TTTTT--
                               * ***  ** *                 *   *** *** * **  *

BRCA2-EX11_17dup-3_-AluSg-RC  AACCTGGGAGGTGGAGGTTGCAGTGAGCCGAGATCGCACCACTGCACTCT
BRCA2-EX11_17dup-5_           -ACTTATTA------AATTAT------ACAA-ATTTATTCCCTGTAATAA
                               ** *   *        **         * * **     * *** * *

BRCA2-EX11_17dup-3_-AluSg-RC  AGCCTGGGCAACAGAGCAAGACTCCATCTAAAAAAAAAACAAAAAAAACC
BRCA2-EX11_17dup-5_           AGCAT---CAA-AAAGCAAAGTACCTGTT-ATATATTATCTCA-----GC
                              *** *   *** * *****    **   * * * *  * *  *      *

BRCA2-EX11_17dup-3_-AluSg-RC  AAAAAAAAAC
BRCA2-EX11_17dup-5_           ATG-ACATGGA
                              *   * *
```

```
CLUSTAL W (1.83) multiple sequence alignment

BRCA2-Ex12dup-3_-AluSc8   TTTATTTATTTATTTATTTATTTTGATACAGAGTCTCACTCTGTCGCCCA
BRCA2-Ex12dup-5_-AluSq10  T--------------------TCTG-------------------------
                          *                    * **

BRCA2-Ex12dup-3_-AluSc8   GGCTGGAGTACAGTGGCACGATCTTGGCTCACTGCAACCTCTGCCTCCTA
BRCA2-Ex12dup-5_-AluSq10  ------------------------------------ACCT----------
                                                              ****

BRCA2-Ex12dup-3_-AluSc8   GGTTCAAGCGACTCTCCTGCTTCAGCCTCCCAAGTAGCCGGGATTACAGG
BRCA2-Ex12dup-5_-AluSq10  ----------------------C------------------------AGG
                                                *                        ***

BRCA2-Ex12dup-3_-AluSc8   CGCGTGCCACCATGCCCGGCTAATTTTTTGTATTTTTACTAGAGAAGGGG
BRCA2-Ex12dup-5_-AluSq10  --------------------------------------------------


BRCA2-Ex12dup-3_-AluSc8   TTTCACCGTGTTAGCCAGGATGGTCTGGATCTCCTGACCTCGTGATCCGC
BRCA2-Ex12dup-5_-AluSq10  ------------------------------------------TGATC---
                                                                    *****

BRCA2-Ex12dup-3_-AluSc8   CCACCTTGGCCTCCCAAGGTGCTGGGATTACAGACGTGAGCCACTGTACC
BRCA2-Ex12dup-5_-AluSq10  -CACCT--GCCTCTCAAAGTGCTGGGATTACAGACATGAGCCACTGTG--
                           *****  ***** *** ***************** ***********

BRCA2-Ex12dup-3_-AluSc8   AGGCCT
BRCA2-Ex12dup-5_-AluSq10  ----CC
                              *
```

```
CLUSTAL W (1.83) multiple sequence alignment

BRCA2-Ex13_23dup-3_  CAGCCTCCAGTAGCACAAGGTCTGCAGGGGCAACTGTACAGGCAGACAAG
BRCA2-Ex13_23dup-5_  ----CTTCATGATCT----GGCTGCTTTGCTATTTTTCCAGTCTGACCTT
                         ** **  * *     * ****   *  *  * * *** * ***

BRCA2-Ex13_23dup-3_  AACCAGGTTT--GTTGCAACAAGATGGCAATGAGAGCACCTGCAGGAAAG
BRCA2-Ex13_23dup-5_  C-TAATGTTCCCCTTGCT-C-TCCTTGCTCCAGGCACACTTGTGTC-T--
                         * ***    ****  *    * **     *  *** **

BRCA2-Ex13_23dup-3_  GGTGACGGTAGTGGAGATCTTACTGAGTTCCAGAGGCCCCATTGAAAGGA
BRCA2-Ex13_23dup-5_  ----AGGCCAATCGACATATTTGTTTGTCTGTTTCCTTCCACTGAAA-TA
                         * *  * * ** ** **  *  **          *** *****  *

BRCA2-Ex13_23dup-3_  TTCGAGGAGATGAAGAGGTAGGAGGAGATGGTGCCCAGAAAGGC------
BRCA2-Ex13_23dup-5_  TACATGCAAAAACAAAATTTGTTACCGTGTTCCCCAGCAAAACAATGTC
                     * *  * * *   * *  *  *      ** * *****  **  *

BRCA2-Ex13_23dup-3_  ---CAC--------ATCCA
BRCA2-Ex13_23dup-5_  TGGCACCTGGTAGGAATTC
                        ***        *
```

```
CLUSTAL W (1.83) multiple sequence alignment

BRCA2-Ex13_23dup-3_-RC  -T---GGATGTGGCCTTTCTGGGCACCATCTCCTCCTACCTCTTCATCTC
BRCA2-Ex13_23dup-5_     CTTCATGATCTGGCTGCTTTGCTATTTTTCCAGTCTGACCTTCTAATGTT
                         *    *** ****   * **       **   **  ****  * ** *

BRCA2-Ex13_23dup-3_-RC  CTCGAATCCTTTCAATGGGGCCTCTGGAACTCAGTAAGATCTCCAC-TAC
BRCA2-Ex13_23dup-5_     CCCC-TTGCTCTCCTTG---CTCCAGGCAC--ACTTGTGTCTAGGCCAAT
                        * *   * ** **  **   *  * ** **  * *    ***   *  *

BRCA2-Ex13_23dup-3_-RC  CGTCACCCTTTCCTGCAGG--TGCTCTCATTGCCATCTTGTTGCAACAAA
BRCA2-Ex13_23dup-5_     CGACATATTTGTTTGTCTGTTTCCTTCCACTGAAATATACATGCAA-AAA
                        ** **   **    **   *  * **  ** **  ** *   ***** ***

BRCA2-Ex13_23dup-3_-RC  CCTGGTTCTTGTCTGCCTGTACAGTTGCCCCTGCAGA-CCTTGT---GCT
BRCA2-Ex13_23dup-5_     CA-AAATTTTGTTACCGTGTT------CCCCAGCAAAACAATGTCTGGCA
                        *     * ****   * ***       **** *** * *  ***   **

BRCA2-Ex13_23dup-3_-RC  ACTGG-AG--GCTG
BRCA2-Ex13_23dup-5_     CCTGGTAGGAATTC
                         **** **    *
```

Figure S8

BRCA2

```
CLUSTAL W (1.83) multiple sequence alignment

BRCA2-Ex14_17dup-3_-AluSz  GGCTGGGCACAGTGGCTCACACCTATAATCCCAGCACTTTGAGAGGCCAA
BRCA2-Ex14_17dup-5_-AluSx  GGCCGGGCGTGGTGGCTCATGCCTGTAATCCCAGAACTTTGGGAGACTAA
                           *** ****   ********  *** ********* ****** *** * **

BRCA2-Ex14_17dup-3_-AluSz  GGTGAATGGATCACTTGAGGTCAGGAGTTCAAGACCAGCCTGGCCAACAT
BRCA2-Ex14_17dup-5_-AluSx  GGCGGGCGGATCACCTGAGGTCAGGAGTTCAAGACCAGCCTGGCCAACAT
                           ** *   ******* ***********************************

BRCA2-Ex14_17dup-3_-AluSz  GGTGAAACCCCGTCTCTACTAAAAATATAAAA------------------
BRCA2-Ex14_17dup-5_-AluSx  AGTGAAACCCTGTCTCTACTAAAAATACAAAATAAATAAATAAATAAATA
                            ********* **************** ****

BRCA2-Ex14_17dup-3_-AluSz  CTTAGCCAGGCATGATGGCATGCTCCTGTAGTCCCAGCTACTGTGGAGGC
BRCA2-Ex14_17dup-5_-AluSx  AATAGCTGAGCGTGGTGGCAGGCACCTGTAATCCCAGCTGCTTGGGAGGC
                             ****   ** ** ***** ** ****** ******** **  ******

BRCA2-Ex14_17dup-3_-AluSz  TGAGGTGGGAGAATCACTTGAACCTGGGACGTAGAGGTTGCAGTGAGCCA
BRCA2-Ex14_17dup-5_-AluSx  TGAGGCAGGAGAATCGTTTGAACCCGGGAGGCAGAGGTTGCAGTGAGCCG
                           *****  ********  ******* **** * *****************

BRCA2-Ex14_17dup-3_-AluSz  AGGTCGCACCACTGCACTCCAGCCTGGGCGACAA-AGTGA--GACCCTGT
BRCA2-Ex14_17dup-5_-AluSx  AGATCGCGCCATTGCACTCCAGCCTGGGCAACAAGAGTGAAAAACTCTGT
                           ** **** *** ***************** **** *****   ** ****

BRCA2-Ex14_17dup-3_-AluSz  CTCAAAGAAAAAAAAAAA
BRCA2-Ex14_17dup-5_-AluSx  CTCAAAAAGAGATAAAAA
                           ****** * * * *****
```

```
CLUSTAL W (1.83) multiple sequence alignment

BRCA2-Ex19dup-3_  -TATA---TAGATGTA----ACATGTTCTATAGGACATGTAATTTACATG
BRCA2-Ex19dup-5_  ATTTCTGCTAACAGTACTCGGCCTGCTCGCTGGTATACCAAACTTGGATT
                   * *    **   ***     * ** **  * * * *   ** **  **

BRCA2-Ex19dup-3_  TCCTATAGAACTATAAGTTACATGTCCTATAGAACTT---ACAGTTCTAT
BRCA2-Ex19dup-5_  CTTTCCTGA-CCCTAGAC--CTTTTCCTCT-GCCCTTATCATCGCTTTTC
                     *   ** *  **     * * **** * *  ***   *  * * *

BRCA2-Ex19dup-3_  AGTTATCTGCAGAAATATTGCTCCTTATGCTTTATTTGCTTAAAATTATC
BRCA2-Ex19dup-5_  AGTGATG-GAGGAAATGTTGGTTGTGTTGATGTAATTATTCAAAG--AGC
                  *** **  *  ***** *** *  *  ** * ** **  * ***   * *

BRCA2-Ex19dup-3_  ACTAGATCATACTATTTTCATAAATAAATGAATATGAAATCATTCACAGG
BRCA2-Ex19dup-5_  A-TACCCTATACAGGTATGATGTATTCTTGAAACTT--ACCATATATTT-
                  * **    ****   * * ** **   ****  *   * ***  *

BRCA2-Ex19dup-3_  CATAC--CTCAGA
BRCA2-Ex19dup-5_  CTTTCTTTTGATA
                  * * *   * * *
```

```
CLUSTAL W (1.83) multiple sequence alignment

BRCA2-Ex19dup-3_-RC  -TCTGAGGTATGCCTGTGAATGATTTCATATTCATTTATTTATGAAAATA
BRCA2-Ex19dup-5_     ATTTCTGCTAA--CAGTACTCG---GCCTGCTCGCTGGTATACCAAACTT
                      * *  * **   * **    *    * *  **  *  * **  *** *

BRCA2-Ex19dup-3_-RC  GTATGATCTAGTGATAATTTTAAGC-AAATAAAGCATAAGGAGCAATATT
BRCA2-Ex19dup-5_     GGATTCTTTCCTGAC---CCTAGACCTTTTCCTCTGCCCTTATCATCGC-
                     * **  * *  ***      ** *    *           * **

BRCA2-Ex19dup-3_-RC  TCTGCAGATAACTATAGAACTGTAAGTTCTATAGGACATGTAACTTATAG
BRCA2-Ex19dup-5_     TTTTCAG-TGATGGAGGAAATGTTGGTTGTGTTGATGTAATTATTCAAAG
                     * * *** * *     *** ***  *** * * *      * * * * **

BRCA2-Ex19dup-3_-RC  TTCTATAGGACATGTAAATTA--------CATGTCCTATAGAACATGTTA
BRCA2-Ex19dup-5_     AGC-ATACCCTATACAGGTATGATGTATTCTTGAAACTTACCATATATTT
                       * ***    **  *  *          * **     **  * ** **

BRCA2-Ex19dup-3_-RC  CA---TCTATATA
BRCA2-Ex19dup-5_     CTTTCTTTTGATA
                     *    * *  ***
```

Figure S8

CDH1

```
CLUSTAL W (1.83) multiple sequence alignment

CDH1-Ex3_16dup-3_-MIRc   GAGACTACTGCAGAGTAGCTGAAAGAGCACAGGGAAGTCAGTAGTCAGGA
CDH1-Ex3_16dup-5_        --GGCCAT-G-TGACTAGGT----------AGGGAAGG--GTGTTCTAGA
                           * * *  *  ** *** *          *******   **  **  **

CDH1-Ex3_16dup-3_-MIRc   AACTTGGATATGAATTTTGTTTCTGTCACCTTTGTGACTGCCCCAAATTA
CDH1-Ex3_16dup-5_        C----GGAGGGGAG-GGCGTTCTAG--ATGGAGGGAACAGC--AAAGACA
                              ***   **     ***   *  *     *  ** **   **   *

CDH1-Ex3_16dup-3_-MIRc   CACGTTAAATGAGTGAACA--TGGATAAGACATTTCAGTCGCTCTCAGTC
CDH1-Ex3_16dup-5_        CACCCCTGAC-ACTGAGCAGAAGGGCAAG----GTCAGTGA---TCAAGG
                         ***     *  * *** **   **  ***     *****     ***

CDH1-Ex3_16dup-3_-MIRc   TCCATTTTCTCATTTATAATGTAGGCATC-AAGCTTTTGGCATAAGGACC
CDH1-Ex3_16dup-5_        GGCAGGGGCTGGC--AGAAG---GGCAGAGGGGTGGCAGCATGTGGAGG
                           **    **     * **    ****     * *    ****  ***

CDH1-Ex3_16dup-3_-MIRc   AATTAAGATAATTCATGTTAAAAGATTTTGTGAAATATATACCCCATACA
CDH1-Ex3_16dup-5_        GCCTTGT---ACCCATCAC-CAGGACTTTGTC-----------TTTCACT
                            *      *  ***     * ** *****                **

CDH1-Ex3_16dup-3_-MIRc   AA
CDH1-Ex3_16dup-5_        TG
```

```
CLUSTAL W (1.83) multiple sequence alignment

CDH1-Ex3_16dup-3_-MIRc-RC  TTTGTATGGGGTATATATTTCACAAAATCTTTTAACATGAATTATCTTAA
CDH1-Ex3_16dup-5_          -GGCCATGTGA-CTAGGTAGGGAAGGGTGTTCTA-GACGGAGGG------
                                *** *   **  *     *   * ** **  * * *

CDH1-Ex3_16dup-3_-MIRc-RC  TTGGTCCTTATGCCAAAAGCTTGATGCCTACATTATAAATGAGAAAAT--
CDH1-Ex3_16dup-5_          GAGGGCGTT---CTA---GATGGA-GGGAACAGCA---AAGACACACCCC
                             ** * **   * *   * * ** *   ***  *   * ** * *

CDH1-Ex3_16dup-3_-MIRc-RC  GGAGACTGAGAGCGACTGAAATGTCTTATCCATGTTCACTCATTTAACGT
CDH1-Ex3_16dup-5_          TGACACTGA--GC---AGAA-GG------GCAAGGTCAGTGAT-CAAGGG
                            ** *****  **    ***  *       ** * *** * **  ** *

CDH1-Ex3_16dup-3_-MIRc-RC  GTAATTTGGGGCAGTCACAAAGGTGACAGAAACAAAATTCATATCCAAGT
CDH1-Ex3_16dup-5_          GCA----GGGGCTGGCAGAAGGGC-AGAGGGGGTGGCAGCATGTGGAGGG
                           * *    ***** * ** ** **  * **          *** *  * *

CDH1-Ex3_16dup-3_-MIRc-RC  TTCCTGACTACTGACTTCCCTGTGCTCTTTCAGCTACTCTGCAGTAGTCT
CDH1-Ex3_16dup-5_          -CCTTG--TACCCATCA-CCAGGACTTTGTCT-------TTCA----CTT
                             * **  ***  *    ** *  ** * **        * **      *

CDH1-Ex3_16dup-3_-MIRc-RC  C
CDH1-Ex3_16dup-5_          G
```

```
CLUSTAL W (1.83) multiple sequence alignment

CDH1-IN2dup-a-3_  CCACTTATGAGTGAGAACATACAATATTTGGTTTTCCATTCCTGAGTTAC
CDH1-IN2dup-a-5_  ------TTGGGTGAGG--------------CTTTTC--TTCCAAA-ATGC
                         ** *****                *****  ****  *  * *

CDH1-IN2dup-a-3_  TTCACTTAGAATACTAATCTCCAGTTCCATTCTTTTTTTTTTTTTAACAC
CDH1-IN2dup-a-5_  -TCACCTTGTGT-CT--------GTTACACATTCCCTTCCAAGGTAGCCC
                   **** * *  * **        *** **   *   **      ** * *

CDH1-IN2dup-a-3_  CACTTATGAGTGAGAACATACAATATTTGGTTTTCCATTCCTGAGTTACT
CDH1-IN2dup-a-5_  CAGATACGCCAGAG--GCTG-AAGCTCTCGTAT-----CTCCCAGAAGCT
                  **  ** *   ***    *  **  * * ** *       *  **   **

CDH1-IN2dup-a-3_  TCACTTAGAATACTAATCTCCAGTTCCATTCTTTTTTTTTTTTTAACACC
CDH1-IN2dup-a-5_  GCCC---AAACCCTAAAATCC----CC-------------GAGAGGCACC
                   * *    **  ****  ***    **                   ****

CDH1-IN2dup-a-3_  TGTCTCTAATCCCAGTTTCTCAGGAGGGTGAGGCGGGAGCAGGCCCCTCC
CDH1-IN2dup-a-5_  TGTCCC-AATCCAA-T-CCTT-GCTGGG--------------TCTCTTTC
                  **** * ***** * *  **  *  ***               * * * *

CDH1-IN2dup-a-3_  TACCCTCCTGTTCGGTCATTTTAGCAGATGACTTTCACCCTCATGCCT
CDH1-IN2dup-a-5_  TTTCTT-----TCTTTCTT-----------TCTTTCTTTC---TTTCT
                  *  * *     **  ** *            *****   *   *  **
```

```
CLUSTAL W (1.83) multiple sequence alignment

CDH1-IN2dup-a-3_-RC   AGGCATGAGGGTGAAAGTCATCTGCTAAAATGACCGAACAGGAGGGTAGG
CDH1-IN2dup-a-5_      ------TTGGGTGAGGCTTTTCTTCCAAAATGCTC-ACCT----------
                              ******   *  *** * ******  * * *

CDH1-IN2dup-a-3_-RC   AGGGGCCTGCTCCCGCCTCACCCTCCTGAGAAACTGGGATTAGAGACAGG
CDH1-IN2dup-a-5_      -TGTGTCTGTTAC-ACATTCCCTTCC-AAG---GTAGCCCCAGATAC--G
                        * * *** * *  * *  ** ***  **    * *    *** **  *

CDH1-IN2dup-a-3_-RC   TGTTAAAAAAAAAAAAAGAATGGAACTGGAGATTAGTATTCTAAGTGAAG
CDH1-IN2dup-a-5_      --C----------CAGAGGCTGAAGCT-----CTCGTAT-CTCCCAGAAG
                                    * **  ** * **      * **** **    ****

CDH1-IN2dup-a-3_-RC   TAACTCAGGAATGGAAAACCAAATATTGTATGTTCTCACTCATAAGTG-G
CDH1-IN2dup-a-5_      CTGCCCA--------AACCCTAAAATC-------C---CCGAGAGGCACC
                         * **        ** ** ** **        *   *  * * *

CDH1-IN2dup-a-3_-RC   TGTTAAAAAAAAAAAAAGAATGGAACTGGAGATTAGTATTCTAAGTGAAG
CDH1-IN2dup-a-5_      TGTCCCAATCC---AATCCT---TGCTGGGTCT---CTTTCTTT------
                      ***   **      **         ****   *     ****

CDH1-IN2dup-a-3_-RC   TAACTCAGGAATGGAAAACCAAATATTGTATGTTCTCACTCATAAGTGG
CDH1-IN2dup-a-5_      ---CTT----------------TCTTTCTTT-CTTTCTTTCT---TTCT
                         **                    ** * *  * **  **     *
```

```
CLUSTAL W (1.83) multiple sequence alignment

CDH1-IN2dup-b-3_-AluSz  TTTTTTTTTTTTTTTTGAGATGGAGTCTTGCTC-TGTCGCCCAGGCTGGA
CDH1-IN2dup-b-5_-AluSp  TTT---TTTTTTTTTTGAGATGGAGCTTTGCTCTTGTTGCCCAGGCTGGA
                        ***   *******************  ****** *** ************

CDH1-IN2dup-b-3_-AluSz  GGGCAGTGGCGCAATCTTGACTCACTGCAACCTCCGCCTCCCAGGTTCAA
CDH1-IN2dup-b-5_-AluSp  GTGCAATGGCACGCTTTTGGCTCACTGCAACCTCCGCCTCCCGGGTTCAA
                        * *** **** *  * *** ********************** *******

CDH1-IN2dup-b-3_-AluSz  GCAATTCTCCTGCCTCAGCCTCCCGAGTAGCTGGGATTACAGGCATGCGC
CDH1-IN2dup-b-5_-AluSp  G---------TGCCCCAGACTCCCAAGTAGCTGGGATTACAGGCATGCGT
                        *         **** *** ***** ************************

CDH1-IN2dup-b-3_-AluSz  CACCACGCCTGGCTAATTTTTGTATTTTTGGTAGAGACAGAGTTTCACCA
CDH1-IN2dup-b-5_-AluSp  CACCACACCCAGCTAATT-TTGCATTTTTAGTAGAGACAAGGTTTCTCCA
                        ****** **  ******* *** ****** *********  ***** ***

CDH1-IN2dup-b-3_-AluSz  TGTGGACCAGGCTGGTCTTGAACTCCTGACCTCAAGTGTTCCACCCA---
CDH1-IN2dup-b-5_-AluSp  CGTTGATCAGGCTGGTCTCGAAGTCCTGACCTCAGGTGATCTGTCCGCCT
                         ** ** *********** *** *********** *** **   **

CDH1-IN2dup-b-3_-AluSz  ---CCTCCCAAAGGGCTGGGATTATAGGCGTGAGCCAGTACGCCCTGC
CDH1-IN2dup-b-5_-AluSp  TGGCCTCCCGAAGTGCTGGGATTACAGGCGTGAGCCACTGCGCCCGGC
                           ****** *** ********** ************ * ***** **
```

Figure S8

CHEK2

```
CLUSTAL W (1.83) multiple sequence alignment

CHEK2-5_UTR_Ex1dup-3_-MER51a  TATTACCAGGTGGAAGGTTCTGATTGGAGTTGTCCAGGTTTTTGGCACGT
CHEK2-5_UTR_Ex1dup-5_         C-TTTTAGGGTTAACTTCTCTGCTTTG-------GAAGCAGCTGGCCAGT
                                **    ***  *    **** ** *        * *    ****  **

CHEK2-5_UTR_Ex1dup-3_-MER51a  TGAACAAATAATTGAACATCATGCATGAACAAGCAATGAAAGCATAGATT
CHEK2-5_UTR_Ex1dup-5_         TTA-CA----CTCC-TC-CCTGGGCTGTACAAA-AATACTTCCTTTGAAG
                              * * **     *    *  *  *  ** ****  ***     * * **

CHEK2-5_UTR_Ex1dup-3_-MER51a  TATTGAAGTGGAAGGCAATAGAAGCTAAAGTACATGTCACAGGATGGGAG
CHEK2-5_UTR_Ex1dup-5_         TGTT--AGGAGA-AGCGGTGAAGGGTAAA--ACCTGGTGAAGAATAAAAC
                              * **  **  **  **  *  * * ****  ** **    ** **   *

CHEK2-5_UTR_Ex1dup-3_-MER51a  CAGGCCTGAGCAAGTGGCTGCAGAGCCCTTGTAGCAAAGTCTTCTGGGGT
CHEK2-5_UTR_Ex1dup-5_         CTGGAGTGGGG-CTTGAATGCATTTCTCTTCCAGG-GAGTTTGCTGGGTG
                              * **  ** *    **  ****   * ***  **   *** * *****

CHEK2-5_UTR_Ex1dup-3_-MER51a  TCAAGTACCCTCTAGAGGTCTCCCACTGTTTACATCCTATGTAAATGAAG
CHEK2-5_UTR_Ex1dup-5_         AC---TGAGCCCCACAGC-CTTCCT-----CGTAACCTATAGAGCTGA-G
                               *   *   * * * **  ** **         * *****  *  *** *

CHEK2-5_UTR_Ex1dup-3_-MER51a  TGCTGGCTCACAGCCCATTAGAGGCCAAAGTGAATTGGAGCTC
CHEK2-5_UTR_Ex1dup-5_         ---TGGTTCTCCTCA----------CATAGTT-------ACAG
                                 *** ** *  *           ** ***         *
```

```
CLUSTAL W (1.83) multiple sequence alignment

CHEK2-5_UTR_Ex1dup-3_-MER51a-RC  GAGCTCCAATTCACTTTGGCCTCTAATGGGCTGTGAGCCAGCACTTCA-T
CHEK2-5_UTR_Ex1dup-5_            CTTTTAGGGTTAACTT------CTC-TGCTTTG-GAAGCAGCTGGCCAGT
                                     *    ** ****      **  **   ** **  ****    ** *

CHEK2-5_UTR_Ex1dup-3_-MER51a-RC  TTACATAGGATGTAAACAGTGGGAGACCTCTAG-AGGGTACTTGAACCCC
CHEK2-5_UTR_Ex1dup-5_            TTACACT------CCTCCCTGGG----CTGTACAAAAATACTT---CCTT
                                 *****           *  ****    ** **  *   *****   **

CHEK2-5_UTR_Ex1dup-3_-MER51a-RC  AGAAGACTTTGCTACAAGGGCTCTGCAGCCACTTGCTCAGGCCTGCTCCC
CHEK2-5_UTR_Ex1dup-5_            TGAAGTGTTAG-GAGAAGCG--GTGAAG------GGTAAAACCTGGTG-A
                                  ****  ** *  * *** *   ** **      * * *  **** *

CHEK2-5_UTR_Ex1dup-3_-MER51a-RC  ATCCTGTGACATGTACTTTAGCTTCTATTGCCTTCCACTTCAATAAATCT
CHEK2-5_UTR_Ex1dup-5_            AGAATAAAACCTGGAGTGGGGCTTG-AATGCATTTCTCTTCCAGGGAGT-
                                 *   *   ** ** * *   ****  * *** ** * **** *   *

CHEK2-5_UTR_Ex1dup-3_-MER51a-RC  ATGCTTTCATTGCTTGTTCATGCATGATGTTCAATTATTTGTTCAACGTG
CHEK2-5_UTR_Ex1dup-5_            ---------TTGCTGGGTGA---CTGAGCCCCACAGC---CTTCCTCGT-
                                          ***** * * *    ***    **        ***  ***

CHEK2-5_UTR_Ex1dup-3_-MER51a-RC  CCAAAAACCTGGACAACTCCAATCAGAACCTTCCACCTGGTAA-TA
CHEK2-5_UTR_Ex1dup-5_            -----AACCTATAGAGCTG--AGTGGTTCTCCTCACATAGTTACAG
                                      *****  * * **   *   *  *    *** * ** *
```

```
CLUSTAL W (1.83) multiple sequence alignment

CHEK2-Ex2_14dup-3_-AluSx        GGCCAGCCGCGGTGGCTCATGCCTGTAATCCCAGCACTTTGGGAAGCCAA
CHEK2-Ex2_14dup-5_-AluSx-Sp-Sx  GGCCAGGCGTGGTGGCTCATGCCTGTAATCCCAGCACTTTGGGAGGCCGA
                                ****** ** ********************************** *** *

CHEK2-Ex2_14dup-3_-AluSx        CGTGGGTGGATCACCTGAGGTCAGGAGTTCGAGACCAGCCTGGCTAACAT
CHEK2-Ex2_14dup-5_-AluSx-Sp-Sx  GATGGGCGGATCACCTGAGATCGGGAGTTCGAGACCAGCCTGACCAACAT
                                  **** ************ ** ******************* * *****

CHEK2-Ex2_14dup-3_-AluSx        TGTGAAACCCCGTCTCTACTGAAAACACAAAAATTAGCCGGGTGTGGTGG
CHEK2-Ex2_14dup-5_-AluSx-Sp-Sx  GGAGAAACCCTGTCTCTACTAAAAATACAA-AATTAGCTGGGCATGGTGG
                                 * ******* ********* **** **** ******* ***  ******

CHEK2-Ex2_14dup-3_-AluSx        CACATGCCTGTAGTATCAGCTACTCGGGAGGTTGAGACAGAAGAATCTCT
CHEK2-Ex2_14dup-5_-AluSx-Sp-Sx  CGGGCTCCTGTAATCCCAGCTACTCAGGAGGCTGAGGCAGGAGAATCGGT
                                *     ****** *  ********* ***** **** *** ******  *

CHEK2-Ex2_14dup-3_-AluSx        TGAAACCAGGAGGTGGAGGTTGCAGTGAGCCGA-ATAGCGCCACTGTACT
CHEK2-Ex2_14dup-5_-AluSx-Sp-Sx  TGAACCTGGGAGGCAGAGGTTGCAGTGAACCGAGATCTCGCCATTGCACT
                                **** *  *****  ************* **** **  ***** ** ***

CHEK2-Ex2_14dup-3_-AluSx        CCAGTCTGAGTGACA-GAGTGAGACTCCATCTCA----------------
CHEK2-Ex2_14dup-5_-AluSx-Sp-Sx  CCAGCCTGGGCAACAAGAGCGAAACTCCATCTCAAAAAAAAAAGAAAAAA
                                **** *** *  *** *** ** ***********

CHEK2-Ex2_14dup-3_-AluSx        -AA
CHEK2-Ex2_14dup-5_-AluSx-Sp-Sx  AAA
                                 **
```

```
CLUSTAL W (1.83) multiple sequence alignment

CHEK2-Ex2_3dup-a-3_-AluSp   -----AAAGATGAGTAAGTTGCTGGGCGTGGTGGCTCACACCTGTAATCC
CHEK2-Ex2_3dup-a-5_-AluSc8  TATAACAAGCTGAGCGGGAGGCCGGGTGTGGTGGCGCATGCCTGTTATCC
                                  *** ****   *  ** *** ******** **  ***** ****

CHEK2-Ex2_3dup-a-3_-AluSp   CAGCACTTTGGGAGGCTGAGGCGGATGGATCACCTGAGGTCAGGAGTTCG
CHEK2-Ex2_3dup-a-5_-AluSc8  CAGCACTTTGGGAGGCCGAGGCGGGCGGATCAC--AAGTTCAGGAGATCG
                            **************** *******  *******   ** ******* ***

CHEK2-Ex2_3dup-a-3_-AluSp   AGACCAGCCTGACCCACATGGAGAAACCCTGTCTCTACTAAAAATACAA-
CHEK2-Ex2_3dup-a-5_-AluSc8  AGACCATCCTGTCTAACATGGCGAAACCCCGTCTCTACTAAAATTACAAA
                            ****** **** *  ****** ******* ************* *****

CHEK2-Ex2_3dup-a-3_-AluSp   -AATAAGCCGGGCATGGTGGTGCATGCCTGTAATCCCAGCTATTCGGGAG
CHEK2-Ex2_3dup-a-5_-AluSc8  AAATTAGCTGGGCATGGTGGCACGTGCCTGTAGTCCCAGCCACTCGGGAG
                             *** *** ***********  * ******** ******* * *******

CHEK2-Ex2_3dup-a-3_-AluSp   GCTGAGGCAGGAGAATCGCTTGAACCCGGGAGGCAGAGGTTGCAGTGAGC
CHEK2-Ex2_3dup-a-5_-AluSc8  GCTGAGGCAGGAGAATCGCTTGAACCTGGGAGGTGGAGGTTGCAGTGAGC
                            ************************** ******  ***************

CHEK2-Ex2_3dup-a-3_-AluSp   CAAGATCGTGCCATTGCACTCTAGCCTGGGCAACAAGAGTGAAACTCTGT
CHEK2-Ex2_3dup-a-5_-AluSc8  CGAGATCGCGCCACCGCACTCCAGCCTGGGCGACA-GAGCGAGACTCCAT
                            * ****** ****  ****** ********* *** *** ** ****  *

CHEK2-Ex2_3dup-a-3_-AluSp   CTCAAAACAAAAAAAAAG
CHEK2-Ex2_3dup-a-5_-AluSc8  CTCAAA----GAAAGAAA
                            ******     *** **
```

Figure S8

CHEK2

```
CLUSTAL W (1.83) multiple sequence alignment

CHEK2-Ex2_3dup-b-3_          GATGCCA-----------------------CTGGAACATA----------
CHEK2-Ex2_3dup-b-5_-AluSc8   TATAACAAGCTGAGCGGGAGGCCGGGTGTGGTGGCGCATGCCTGTTATCC
                              **  **                        ***  ***

CHEK2-Ex2_3dup-b-3_          CAGCGTTATGAATAG--------------CTACAAGCATAG---A--GGG
CHEK2-Ex2_3dup-b-5_-AluSc8   CAGCACTTTGGGAGGCCGAGGCGGGCGGATCACAAGTTCAGGAGATCGAG
                             ****  * **    *                *****   **   *  * *

CHEK2-Ex2_3dup-b-3_          ACACTTGTGCCTGG-GTGGCCAG--CAGGTGTCTGTCA-----CCAGACA
CHEK2-Ex2_3dup-b-5_-AluSc8   ACCATCCTGTCTAACATGGCGAAACCCCGTCTCTACTAAAATTACAAAAA
                             **  *  ** **    **** *   *  ** ***   *      ** * *

CHEK2-Ex2_3dup-b-3_          AGTGCCTGCCTCTTGTG-TACCAGCCTGAA-TGAAATATACTCAGGTGGC
CHEK2-Ex2_3dup-b-5_-AluSc8   ATTAGCTGGGCATGGTGGCACGTGCCTGTAGTCCCAGCCACTCGGGAGGC
                             * *  ***    * ***  **  ***** * *   *   **** ** ***

CHEK2-Ex2_3dup-b-3_          TCAG-------A----TTTGTGCCAGAGAC-TGG----------------
CHEK2-Ex2_3dup-b-5_-AluSc8   TGAGGCAGGAGAATCGCTTGAACCTGGGAGGTGGAGGTTGCAGTGAGCCG
                             * **       *     ***  ** * **  ***

CHEK2-Ex2_3dup-b-3_          --ATCC--TTAGGTTATTCTAGG---GAGGTCAAAACCTG--------TC
CHEK2-Ex2_3dup-b-5_-AluSc8   AGATCGCGCCACCGCACTCCAGCCTGGGCGACAGAGCGAGACTCCATCTC
                               ***     *    * ** **    *  * ** * *  *        **

CHEK2-Ex2_3dup-b-3_          A------CCTG
CHEK2-Ex2_3dup-b-5_-AluSc8   AAAGAAAGAAA
                             *
```

```
CLUSTAL W (1.83) multiple sequence alignment

CHEK2-Ex2_3dup-b-3_-RC       -----CA-GGTGA--------CAGGTTTT---GA--CCTCCCTAGAAT--
CHEK2-Ex2_3dup-b-5_-AluSc8   TATAACAAGCTGAGCGGGAGGCCGGGTGTGGTGGCGCATGCCTGTTATCC
                                  ** * ***        * ** * *   *   * * ***   **

CHEK2-Ex2_3dup-b-3_-RC       ---AACCTAAGGATCCAGTCTCTGG-----CACAAATCT--------GA-
CHEK2-Ex2_3dup-b-5_-AluSc8   CAGCACTTTGGGAGGCCGAGGCGGGCGGATCACAAGTTCAGGAGATCGAG
                                 ** *  ***  * *   * **     ***** *          **

CHEK2-Ex2_3dup-b-3_-RC       GCCA-CCTGAGTA-TATTTC---ATTCAGG-----CTG---GTACACAAG
CHEK2-Ex2_3dup-b-5_-AluSc8   ACCATCCTGTCTAACATGGCGAAACCCCGTCTCTACTAAAATTACAAAAA
                              *** ****  **  **  *   *  * *      **     **** **

CHEK2-Ex2_3dup-b-3_-RC       A-------GGCA----GGCACTTGTCTG--GTGACAGACACCTGCTGGCC
CHEK2-Ex2_3dup-b-5_-AluSc8   ATTAGCTGGGCATGGTGGCACGTGCCTGTAGTCCCAGCCACTCG--GGAG
                             *       ****    ***** ** ***  **  *** ***  *  **

CHEK2-Ex2_3dup-b-3_-RC       ACCCAGGCACAAGTGTCCCTCTATGC-------------TTGTAGCTATT
CHEK2-Ex2_3dup-b-5_-AluSc8   GCTGAGGCAGGAGAATCGCTTGAACCTGGGAGGTGGAGGTTGCAGTGAGC
                              *  *****  **  ** **  *  *             *** **  *

CHEK2-Ex2_3dup-b-3_-RC       C-ATAACGC-----------------------------------TGTATG
CHEK2-Ex2_3dup-b-5_-AluSc8   CGAGATCGCGCCACCGCACTCCAGCCTGGGCGACAGAGCGAGACTCCATC
                             * * * ***                                   *  **

CHEK2-Ex2_3dup-b-3_-RC       TTCCAGTGGCATC
CHEK2-Ex2_3dup-b-5_-AluSc8   TCAAAGAAAGAAA
                             *   **    *
```

```
CLUSTAL W (1.83) multiple sequence alignment

CHEK2-Ex5_6dup-3_-AluY  G----GGCGCGGTGGCTCACGCCTGTAATCCCAGCACTTTGGGAAGCCGG
CHEK2-Ex5_6dup-5_-AluY  GGCCGAGTGCGGTGGCTCACGCCTGTAATCCCAGCACTTTGGGAGGCCGA
                        *     * ************************************ ****

CHEK2-Ex5_6dup-3_-AluY  GGCGGGTGGATCATGAGGTCAGGAGATCAAGACCATCCTGGCTAACACGG
CHEK2-Ex5_6dup-5_-AluY  GATGGGAGGATCACGAGGTCAGGAGATCAAGACCATCCTGGCTAACACAG
                        *  *** ****** ********************************** *

CHEK2-Ex5_6dup-3_-AluY  TGAAATCCCGTCTCTACTAAAAATACAAAAAAAAATTAGCCGGGCGTGGT
CHEK2-Ex5_6dup-5_-AluY  TGAAACCCC-CCTCTACTAAAAATACAAA--AAGATTAGCCGGGCGTGGT
                        ***** ***  ******************  ** ****************

CHEK2-Ex5_6dup-3_-AluY  GGCGGGTGCGTGTAGTCCCAGCTACTGGAGAGGCTGAGGCAGGAGAATGG
CHEK2-Ex5_6dup-5_-AluY  GGCGGGTACCTGTAGTCCCAGCTACTCGGGAGGCTGAGGCA---GAATGG
                        ******* * **************** * ************   ******

CHEK2-Ex5_6dup-3_-AluY  CGTGAACCCGGGAGGCGGAGCTTGCAGTGAGCCGAGATTGTGCCACTGCA
CHEK2-Ex5_6dup-5_-AluY  CGTGAACCCGGGAGGCGGAGCTTGCGGTGAGCCGAGATCACGCCACTGCA
                        ************************* ************   *********

CHEK2-Ex5_6dup-3_-AluY  CTCCAGCCTGGGCAACAGAGCGAGACTCCGTCTCACAAAAAAAAAAAAAA
CHEK2-Ex5_6dup-5_-AluY  CTCCAGCCTGGGCGACTGAGCGAGACTCCATCTCAAAAA--AAA------
                        ************* ** ************ ***** ***  ***

CHEK2-Ex5_6dup-3_-AluY  AAAAAAAAGATAAA
CHEK2-Ex5_6dup-5_-AluY  -AAAAAAAGA-AAA
                         ********* ***
```

Figure S8

CHEK2

```
CLUSTAL W (1.83) multiple sequence alignment

PALB2-Ex11dup-a-3_-AluY     GGCCAGGCGCGGTGGCTCACGCCTGTAATCCCAGCACTTTGGGAGGCTAA
PALB2-Ex11dup-a-5_-AluYa5   GGCCAGGCGCGGTGGCTCACGCCTGTAATCCCAGCACTTTGGGAGGCCGA
                            ***********************************************  *

PALB2-Ex11dup-a-3_-AluY     GGCGGGCGGATCATGAGGTCAGGAGATCGAGACCATCCTGGCTAACACAG
PALB2-Ex11dup-a-5_-AluYa5   GGCGGGCGGATCACGAGGTCAGGAGATCAAGACCATCCCGGCTAAAACGG
                            ************* ************** ********* ****** ** *

PALB2-Ex11dup-a-3_-AluY     TGAAACCCCG-CTCTACTAAAAGTACAAAAAATTAGCCGGGCGTGGTGGC
PALB2-Ex11dup-a-5_-AluYa5   TGAAACCCCGTCTCTACTAAAAATACAAAAAATTAGCCGGGCGTAGTGGC
                            ********** *********** ********************* *****

PALB2-Ex11dup-a-3_-AluY     GGGCGCCTGTAGTTCCAGCTACTCGGGAGGCTGAGGCAGGAGAATGGCGT
PALB2-Ex11dup-a-5_-AluYa5   GGGCGCCTGTAGTCCCAGCTACTCGGGAGGCTGAGGCAGGAGAATGGCGT
                            ************* ************************************

PALB2-Ex11dup-a-3_-AluY     GAACCCAGGAGGCAGAGCTTGCAGTGAGCAGACATCGCACCACTGCACGC
PALB2-Ex11dup-a-5_-AluYa5   GAACCCGGGAGGCGGAGCTTGCAGTGAGCCGAGATCCCGCCACTGCACTC
                            ****** ****** *************** ** *** * ********* *

PALB2-Ex11dup-a-3_-AluY     CAGCCTGGGCGACAGTGCAAGACTCTGTCTCAAAAA------------AA
PALB2-Ex11dup-a-5_-AluYa5   CAGCCTGGGCCACAGAGCGAGACTCCGTCTCAAAAAAAAAAAAAAAAAAA
                            ********** **** ** ****** **********            **

PALB2-Ex11dup-a-3_-AluY     AAAA
PALB2-Ex11dup-a-5_-AluYa5   AAAA
                            ****
```

```
CLUSTAL W (1.83) multiple sequence alignment

PALB2-Ex11dup-b-3_-AluSx     ---------------------------------AACGCTTTGGGAGGCCA
PALB2-Ex11dup-ba-5_-AluSz6   GGCCGGGCACGGGTGGCTCATGCCTATAATCCCAGCACTTTGGGAGGCCA
                                                              * * *************

PALB2-Ex11dup-b-3_-AluSx     AAGTGGGTGGAACACCTGAGGTAAGGAGTTTGAGACCAGCCTGACCAATA
PALB2-Ex11dup-ba-5_-AluSz6   AGGCAGGCAGATCACTTGAGGCCAAGTGTTCGAGACCAGCCTGGCCAACA
                             * *  **  ** *** *****  * * *** ************ **** *

PALB2-Ex11dup-b-3_-AluSx     TGGTGAAAGCCCACCTCTACTAAAAATACAAAAATTAGGTGGGCGTGGTG
PALB2-Ex11dup-ba-5_-AluSz6   AGGCAAAACCCCAGCTCTACTAAAAATACAAAAATTAGCCAGGCGTGGTG
                              **  *** **** ************************   *********

PALB2-Ex11dup-b-3_-AluSx     GCGCATGCCTGTAATCCCAGCTACTTGGGAGACTGAGGAAAGAGAATTGC
PALB2-Ex11dup-ba-5_-AluSz6   GTGCACACCTGTAATTCCAGCTACTCAGGAGGCTGAGGTAGGAGAATCGC
                             * ***  ******** *********  **** ****** * ****** **

PALB2-Ex11dup-b-3_-AluSx     TTGAACCCAGGAGGCGGAGGTTGCAGTGAGCCGAGATTGCACGATTGCAC
PALB2-Ex11dup-ba-5_-AluSz6   TTGAACCCAGGAGGCGGAGGTTGCAGTGAGTCAAGATTGCGCCACTGCAC
                             ****************************** * ******* * * *****

PALB2-Ex11dup-b-3_-AluSx     TCCAGCCTGGGCGACAGAGCGAGACTCCGTCTCAAAAAACAAAAAGAAAA
PALB2-Ex11dup-ba-5_-AluSz6   TCCAGCCTGGGCGACAGAGCAAGATTCTGTCTCAAAAAA---AAATAATA
                             ******************** *** ** ***********   *** ** *

PALB2-Ex11dup-b-3_-AluSx     AAAAAAA
PALB2-Ex11dup-ba-5_-AluSz6   ATTTTTT
                             *
```

```
CLUSTAL W (1.83) multiple sequence alignment

PALB2-Ex13_3_UTRdup-3_        ACTCCAGCCTGGGCAACACAGTGAGACTCTGTCTGAAAAAAAAAAAAAAA
PALB2-Ex13_3_UTRdup-5_-AluY   ----CA----GGGTCTTGCTTTGTTGCCCAGGCTG---------------
                                  **    ***     *  **   * * * ***

PALB2-Ex13_3_UTRdup-3_        AGAGACCAGAGGCAGGAGCATGAGAGGGTAGTAGGATCAAGTATTTTGGG
PALB2-Ex13_3_UTRdup-5_-AluY   -GAGTGCAGTGGCACGATCT---CAGCTCACTGCAACTCTGCCTCCCGGG
                               ***  *** **** ** *     **   * *   *    *  *   ***

PALB2-Ex13_3_UTRdup-3_        GCTACGCTACAGCCAAGGTATCAAACTAGCTGCTGCTGGTCATT--GTTC
PALB2-Ex13_3_UTRdup-5_-AluY   TTCACACCAT------CTTCCCATCTCAGCTTCC-CAAGTAGCTGGGACT
                                 ** * *         *  **    **** *  *  **   *  *

PALB2-Ex13_3_UTRdup-3_        TCAGGATCCTACTAAATAACCCTAAATAGCACTTACTGTGTTAAGTGCCA
PALB2-Ex13_3_UTRdup-5_-AluY   ACAGGTACATGCCA--CCACGCT-----TGGCTAATTTTTTTT--TTTTT
                               ****  * * * *    ** **        ** * * * **   *

PALB2-Ex13_3_UTRdup-3_        GC
PALB2-Ex13_3_UTRdup-5_-AluY   TA
```

```
CLUSTAL W (1.83) multiple sequence alignment

PALB2-Ex13_3_UTRdup-3_        GCTGGCACTTAACACAGTAAGTGCTATTTAGGGTTATTTAGTAGGATCCT
PALB2-Ex13_3_UTRdup-5_-AluY   -CAGGGTCTTG-CTTTGTT-GCCCAGGCTGGAGTGCAGTGGCACGATC-T
                               * **  ***  *   **  *  *    * * **    * * * **** *

PALB2-Ex13_3_UTRdup-3_        GAGAACAATGA-CCAGCAGCAGCTAGTTTGATACCTTGGCTGTAGCGTAG
PALB2-Ex13_3_UTRdup-5_-AluY   CAGCTCACTGCAACTCTGCCTCCCGGGTTCACACCATCTTCCCATCTCAG
                               **  ** **   *     *  *  * ** * *** *      * *  **

PALB2-Ex13_3_UTRdup-3_        CC-CCAAAATACTTGATCCTACTACCCTCTCATGCTCCTGCCTCTGGTCT
PALB2-Ex13_3_UTRdup-5_-AluY   CTTCCCAAGTAGCTGG---GACTACAGGTACATGCCACCACGCTTGG-CT
                              *  ** ** **  **     *****     *****  *  *   *** **

PALB2-Ex13_3_UTRdup-3_        CTTTTTTTTTTTTTTTTCAGACAGAGTCTCACTGTGTTGCCCAGGCTGGA
PALB2-Ex13_3_UTRdup-5_-AluY   AATTTTTTTTTTT--TT---------------------------------
                                ***********  **

PALB2-Ex13_3_UTRdup-3_        GT
PALB2-Ex13_3_UTRdup-5_-AluY   TA
```
