# Supplementary material for: DNA breakpoint assay reveals a majority of gross duplications occur in tandem reducing VUS classifications in breast cancer predisposition genes
Source: Genet Med. 2018 Jul 28;21(3):683–93. doi: 10.1038/s41436-018-0092-7 (PMC6752314; doi:10.1038/s41436-018-0092-7)
Supplement: Supplementary file 12 — Supplementary Table S1 [file 41436_2018_92_MOESM12_ESM.pdf]

Table S1: Breakpoint Identification for 147 Probands

Gene	Proband Number	Genomic coordinates	Breakpoints	Reported Nomenclature[b] (coding exons)	Tandem Finding
ATM (NM_000051)	1	-	-	EX6_3'UTRdup	Low quality sample
	2	-	-	EX16_60dup	Lack of probe coverage
	3	-	-	EX16_60dup	Lack of probe coverage
	4	-	-	EX16_60dup	Lack of probe coverage
	5	chr11:108147908-chr11:108156805	c.3285-2309_3994-1522dup	EX22_25dup	Tandem duplication
	6	-	-	EX61_3'UTRdup	Pseudogene interference
	7	-	-	EX61_3'UTRdup	Pseudogene interference
	8	-	-	EX61_3'UTRdup	Pseudogene interference
	9	-	-	EX61_3'UTRdup	Pseudogene interference
	10	-	-	EX61_3'UTRdup	Pseudogene interference
	11	-	-	EX61_3'UTRdup	Pseudogene interference
	12	-	-	EX61_3'UTRdup	Pseudogene interference
	13	-	-	EX61_3'UTRdup	Pseudogene interference
	14	-	-	EX61_3'UTRdup	Pseudogene interference
	15	-	-	EX61_3'UTRdup	Pseudogene interference
	16	-	-	EX61_3'UTRdup	Pseudogene interference
	17	-	-	EX61_3'UTRdup	Pseudogene interference
	18	-	-	EX61_3'UTRdup	Pseudogene interference
	19	-	-	EX61_3'UTRdup	Pseudogene interference
BRCA1 (NM_007294)	20	chr17:41276456-chr17:41320885	c.-232-43385_-19-324dup	5'UTRdup	Tandem duplication
	21	chr17:41271776-chr17:41308690	c.-232-31190_81-3980dup	5'UTR_EX1dup	Tandem duplication
	22	chr17:41271776-chr17:41308690	c.-232-31190_81-3980dup	5'UTR_EX1dup	Tandem duplication
	23[c]	chr17:41271776-chr17:41308690	c.-232-31190_81-3980dup	5'UTR_EX1dup	Tandem duplication
	24	-	-	5'UTR_EX1dup	Low quality sample
	25	chr17:41249296-chr17:41320077	c.-232-42577_558dup	5'UTR_EX6dup	Tandem duplication
	26	chr17:41249296-chr17:41320077	c.-232-42577_558dup	5'UTR_EX6dup	Tandem duplication
	27	-	-	5'UTR_EX9dup	Unknown
	28[c]	chr17:41243444-chr17:41309441	c.-232-31941_4096+8	5'UTR_EX9dup	Tandem duplication
	29	chr17:41202532-chr17:41310341	c.-232-32841_5332+548dup	5'UTR_EX19dup	Tandem duplication
	30	chr17:41200315-chr17:41975558	c.-232-698058_5407-595dup	5'UTR_EX20dup	Tandem duplication
	31	chr17:41200315-chr17:41975558	c.-232-698058_5407-595dup	5'UTR_EX20dup	Tandem duplication
	32	chr17:41267115-chr17:41269593	c.81-1797_134+628dup	EX2dup	Tandem duplication
	33	chr17:41231983-chr17:41265230	c.134+2513_4357+2438dup	EX3_11dup	Tandem duplication
	34	chr17:41231983-chr17:41265228	c.134+2515_4357+2438dup	EX3_11dup	Tandem duplication
	35	chr17:41251088-chr17:41254410	c.441+1729_547+704dup	EX6dup	Tandem duplication
	36	chr17:41227772-chr17:41236203	c.4186-1611_4484+733dup	EX11_12dup	Tandem duplication
	37	chr17:41227695-chr17:41236204	c.4186-1612_4484+810dup	EX11_12dup	Tandem duplication
	38	chr17:41222203-chr17:41236327	c.4186-1735_4986+742dup	EX11_14dup	Tandem duplication
	39	chr17:41230265-chr17:41236424	c.4186-1832_4358-1634dup	EX11dup	Tandem duplication
	40	chr17:41230265-chr17:41236424	c.4186-1832_4358-1634dup	EX11dup	Tandem duplication
	41	chr17:41230265-chr17:41236424	c.4186-1832_4358-1634dup	EX11dup	Tandem duplication
	42	chr17:41230265-chr17:41236424	c.4186-1832_4358-1634dup	EX11dup	Tandem duplication
	43	chr17:41230265-chr17:41236424	c.4186-1832_4358-1634dup	EX11dup	Tandem duplication
	44	chr17:41230265-chr17:41236424	c.4186-1832_4358-1634dup	EX11dup	Tandem duplication
	45	chr17:41230265-chr17:41236424	c.4186-1832_4358-1634dup	EX11dup	Tandem duplication
	46	chr17:41230265-chr17:41236424	c.4186-1832_4358-1634dup	EX11dup	Tandem duplication
	47	chr17:41230265-chr17:41236424	c.4186-1832_4358-1634dup	EX11dup	Tandem duplication
	48	chr17:41230265-chr17:41236424	c.4186-1832_4358-1634dup	EX11dup	Tandem duplication
	49	chr17:41230265-chr17:41236424	c.4186-1832_4358-1634dup	EX11dup	Tandem duplication
	50[a]	chr17:41230265-chr17:41236424	c.4186-1832_4358-1634dup	EX11dup	Tandem duplication
	51[a]	chr17:41230265-chr17:41236424	c.4186-1832_4358-1634dup	EX11dup	Tandem duplication
	52	chr17:41230265-chr17:41236424	c.4186-1832_4358-1634dup	EX11dup	Tandem duplication
	53	chr17:41230265-chr17:41236424	c.4186-1832_4358-1634dup	EX11dup	Tandem duplication
	54	chr17:41230265-chr17:41236424	c.4186-1832_4358-1634dup	EX11dup	Tandem duplication
	55	chr17:41230265-chr17:41236424	c.4186-1832_4358-1634dup	EX11dup	Tandem duplication
	56	chr17:41230265-chr17:41236424	c.4186-1832_4358-1634dup	EX11dup	Tandem duplication
	57	chr17:41222209-chr17:41230377	c.4358-1746_4986+736dup	EX12_14dup	Tandem duplication
	58	-	-	EX12_14dup	Unknown
	59	-	-	EX12_15dup	Unknown
	60	chr17:41212780-chr17:41218700	c.5074+925_5193+2570dup	EX16_17dup	Tandem duplication
	61[a]	chr17:41212780-chr17:41218700	c.5074+925_5193+2570dup	EX16_17dup	Tandem duplication
	62	chr17:41212780-chr17:41218700	c.5074+925_5193+2570dup	EX16_17dup	Tandem duplication
	63	chr17:41212780-chr17:41218700	c.5074+925_5193+2570dup	EX16_17dup	Tandem duplication
	64[a]	chr17:41206970-chr17:41218658	c.5074+967_5277+2099dup	EX16_18dup	Tandem duplication
	65	chr17:41206970-chr17:41218658	c.5074+967_5277+2099dup	EX16_18dup	Tandem duplication
	66	chr17:41206970-chr17:41218660	c.5074+965_5277+2099dup	EX16_18dup	Tandem duplication
	67[a]	chr17:41200793-chr17:41203933	c.5278-799_5406+345dup	EX19_20dup	Tandem duplication
	68	chr17:41200793-chr17:41203933	c.5278-799_5406+345dup	EX19_20dup	Tandem duplication
	69	chr17:41190441-chr17:41200561	c.5406+577_*1383+5871dup	EX21_3'UTRdup	Tandem duplication
BRCA2 (NM_000059)	70	-	-	5'UTR_3'UTRdup	Lack of probe coverage
	71	chr13:32897384-chr13:32899700	c.317-182_425+3799dup	EX3dup	Tandem duplication
	72	chr13:32899730-chr13:32913272	c.425+409_4780dup	EX4_10dup(partial)	Tandem duplication
	73	chr13:32918108-chr13:32923380	c.6842-587_7007+2347dup	EX11_12dup	Tandem duplication
	74	chr13:32918108-chr13:32923321	c.6842-587_7007+2288dup	EX11_12dup	Tandem duplication
	75	chr13:32918023-chr13:32943027	c.6842-672_8332-1512dup	EX11_17dup	Tandem duplication
	76	chr13:32918880-chr13:32926230	c.6937+90_7008-2768dup	EX12dup	Tandem duplication
	77	chr13:32927733-chr13:32958444	c.7008-1265_9256+4162dup	EX13_23dup	Tandem duplication
	78	chr13:32927733-chr13:32958444	c.7008-1265_9256+4162dup	EX13_23dup	Tandem duplication
	79	chr13:32929782-chr13:32940707	c.7435+357_8331+3037dup	EX14_17dup	Tandem duplication
	80	chr13:32929782-chr13:32940803	c.7435+357_8331+3133dup	EX14_17dup	Tandem duplication
	81	chr13:32944639-chr13:32945710	c.8432_8632+473dup	EX19dup	Tandem duplication

CDH1 (NM_004360)	82	-	-	5'UTR_3'UTRdup	Lack of probe coverage
	83	-	-	5'UTR_3'UTRdup	Lack of probe coverage
	84	-	-	5'UTR_3'UTRdup	Lack of probe coverage
	85	-	-	EX3_3'UTRdup	Low quality sample
	86	chr16:68795734-chr16:68924281	c.163+23420_*2042+54837dup	EX3_3'UTRdup	Tandem duplication
	87	chr16:68777120-chr16:68818706	c.163+4806_164-16867dup	IN2dup (a)	Tandem duplication
	88	chr16:68777120-chr16:68818706	c.163+4806_164-16867dup	IN2dup (a)	Tandem duplication
	89	chr16:68802010-chr16:68826292	c.163+29696_164-9281dup	IN2dup (b)	Tandem duplication
	90	chr16:68802010-chr16:68826292	c.163+29696_164-9281dup	IN2dup (b)	Tandem duplication
	91	chr16:68802010-chr16:68826292	c.163+29696_164-9281dup	IN2dup (b)	Tandem duplication
	92	chr16:68802010-chr16:68826292	c.163+29696_164-9281dup	IN2dup (b)	Tandem duplication
	93	chr16:68802010-chr16:68826292	c.163+29696_164-9281dup	IN2dup (b)	Tandem duplication
	94	chr16:68802010-chr16:68826292	c.163+29696_164-9281dup	IN2dup (b)	Tandem duplication
	95	chr16:68802010-chr16:68826292	c.163+29696_164-9281dup	IN2dup (b)	Tandem duplication
	96	chr16:68802010-chr16:68826292	c.163+29696_164-9281dup	IN2dup (b)	Tandem duplication
	97	chr16:68802010-chr16:68826292	c.163+29696_164-9281dup	IN2dup (b)	Tandem duplication
	98	chr16:68802010-chr16:68826292	c.163+29696_164-9281dup	IN2dup (b)	Tandem duplication
	99	-	-	3'UTRdup	Unknown
CHEK2 (NM_007194)	100	chr22:29130189-chr22:29314266	c.-72-176444_319+202dup	5'UTR_EX1dup	Tandem duplication
	101	-	-	5'UTR_3'UTRdup	Lack of probe coverage
	102	chr22:29056775-chr22:29128522	c.319+1869_*154+26956dup	EX2_3'UTRdup	Tandem duplication
	103	chr22:29056775-chr22:29128522	c.319+1869_*154+26956dup	EX2_3'UTRdup	Tandem duplication
	104	chr22:29056775-chr22:29128522	c.319+1869_*154+26956dup	EX2_3'UTRdup	Tandem duplication
	105	chr22:29056775-chr22:29128522	c.319+1869_*154+26956dup	EX2_3'UTRdup	Tandem duplication
	106	chr22:29056775-chr22:29128522	c.319+1869_*154+26956dup	EX2_3'UTRdup	Tandem duplication
	107	chr22:29056775-chr22:29128522	c.319+1869_*154+26956dup	EX2_3'UTRdup	Tandem duplication
	108	chr22:29056775-chr22:29128522	c.319+1869_*154+26956dup	EX2_3'UTRdup	Tandem duplication
	109	chr22:29056775-chr22:29128522	c.319+1869_*154+26956dup	EX2_3'UTRdup	Tandem duplication
	110	chr22:29056775-chr22:29128522	c.319+1869_*154+26956dup	EX2_3'UTRdup	Tandem duplication
	111	chr22:29056775-chr22:29128522	c.319+1869_*154+26956dup	EX2_3'UTRdup	Tandem duplication
	112	chr22:29056775-chr22:29128522	c.319+1869_*154+26956dup	EX2_3'UTRdup	Tandem duplication
	113	chr22:29056775-chr22:29128522	c.319+1869_*154+26956dup	EX2_3'UTRdup	Tandem duplication
	114	chr22:29056775-chr22:29128522	c.319+1869_*154+26956dup	EX2_3'UTRdup	Tandem duplication
	115	chr22:29056775-chr22:29128522	c.319+1869_*154+26956dup	EX2_3'UTRdup	Tandem duplication
	116	chr22:29056775-chr22:29128522	c.319+1869_*154+26956dup	EX2_3'UTRdup	Tandem duplication
	117	chr22:29056775-chr22:29128522	c.319+1869_*154+26956dup	EX2_3'UTRdup	Tandem duplication
	118	chr22:29056775-chr22:29128522	c.319+1869_*154+26956dup	EX2_3'UTRdup	Tandem duplication
	119	chr22:29056775-chr22:29128522	c.319+1869_*154+26956dup	EX2_3'UTRdup	Tandem duplication
	120	chr22:29056775-chr22:29128522	c.319+1869_*154+26956dup	EX2_3'UTRdup	Tandem duplication
	121	chr22:29056775-chr22:29128522	c.319+1869_*154+26956dup	EX2_3'UTRdup	Tandem duplication
	122	chr22:29056775-chr22:29128522	c.319+1869_*154+26956dup	EX2_3'UTRdup	Tandem duplication
	123	chr22:29056775-chr22:29128522	c.319+1869_*154+26956dup	EX2_3'UTRdup	Tandem duplication
	124	-	-	EX2_3'UTRdup	Low quality sample
	125	chr22:29056775-chr22:29128522	c.319+1869_*154+26956dup	EX2_3'UTRdup	Tandem duplication
	126	chr22:29056775-chr22:29128522	c.319+1869_*154+26956dup	EX2_3'UTRdup	Tandem duplication
	127	chr22:29056775-chr22:29128522	c.319+1869_*154+26956dup	EX2_3'UTRdup	Tandem duplication
	128	chr22:29056775-chr22:29128522	c.319+1869_*154+26956dup	EX2_3'UTRdup	Tandem duplication
	129	chr22:29056775-chr22:29128522	c.319+1869_*154+26956dup	EX2_3'UTRdup	Tandem duplication
	130	chr22:29056775-chr22:29128522	c.319+1869_*154+26956dup	EX2_3'UTRdup	Tandem duplication
	131	chr22:29056775-chr22:29128522	c.319+1869_*154+26956dup	EX2_3'UTRdup	Tandem duplication
	132	chr22:29056775-chr22:29128522	c.319+1869_*154+26956dup	EX2_3'UTRdup	Tandem duplication
	133	chr22:29056775-chr22:29128522	c.319+1869_*154+26956dup	EX2_3'UTRdup	Tandem duplication
	134	chr22:29056775-chr22:29128522	c.319+1869_*154+26956dup	EX2_3'UTRdup	Tandem duplication
	135	chr22:29056775-chr22:29128522	c.319+1869_*154+26956dup	EX2_3'UTRdup	Tandem duplication
	136	chr22:29056775-chr22:29128522	c.319+1869_*154+26956dup	EX2_3'UTRdup	Tandem duplication
	137	chr22:29120649-chr22:29123157	c.320-1802_592+316dup	EX2_3dup (a)	Tandem duplication
	138	chr22:29120649-chr22:29123150	c.320-1795_592+316dup	EX2_3dup (a)	Tandem duplication
	139	chr22:29116685-chr22:29123404	c.320-2049_593-1212dup	EX2_3dup (b)	Tandem duplication
	140	-	-	EX4dup	Low quality sample
	141	chr22:29104029-chr22:29109587	c.684-1582_846+1965dup	EX5_6dup	Tandem duplication
	142	-	-	EX7dup	Lack of probe coverage
	143	-	-	EX7dup	Lack of probe coverage
PALB2 (NM_024675)	144	chr16:23621089-chr16:23626223	c.3114-811_3202-1756dup	EX11dup (a)	Tandem duplication
	145[a]	chr16:23621089-chr16:23626223	c.3114-811_3202-1756dup	EX11dup (a)	Tandem duplication
	146	chr16:23624145-chr16:23631203	c.3113+1480_3201+1180dup	EX11dup (b)	Tandem duplication
	147[c]	chr16:23601913-chr16:23617570	c.3350+1615_*297+12570	EX13_3'UTRdup	Tandem duplication

[a] Multiple family members were tested and found to have the same breakpoints
[b] Alterations with (a) or (b) after their reported nomenclature indicate disparate breakpoints
[c] Indicates that breakpoints for this individual were estimated due to low NGS read-depth and actual breakpoints could differ by 200-300nt
